# The Agency, Resources, and Institutional Structures for Sanitation-related Empowerment (ARISE) Scales: Development and validation of measures of women’s empowerment in urban sanitation for low- and middle-income countries

**DOI:** 10.1016/j.worlddev.2023.106183

**Published:** 2023-04

**Authors:** Sheela S. Sinharoy, Shauna McManus, Amelia Conrad, Madeleine Patrick, Bethany A. Caruso

**Affiliations:** a Hubert Department of Global Health, Rollins School of Public Health, Emory University, Atlanta, GA, United States of America; b Department of Biostatistics and Bioinformatics, Rollins School of Public Health, Emory University, Atlanta, GA, United States of America; c Gangarosa Department of Environmental Health, Rollins School of Public Health, Emory University, Atlanta, GA, United States of America

**Keywords:** Gender, Reliability, Scale validation, Psychometrics, India, Uganda

## Abstract

Sustainable Development Goal 6 aims to ensure access to water and sanitation for all, and target 6.2 emphasizes “paying special attention to the needs of women and girls”. Research documenting how water, sanitation and hygiene (WASH) conditions impact women’s and girls’ lives is growing. However, no rigorously validated survey instruments exist for measuring empowerment within the WASH sector. The objective of our study was to develop and validate survey instruments to measure sub-domains of women’s empowerment in relation to sanitation in urban areas of low- and middle-income countries. We followed a multi-phased, theory-informed approach that included factor analysis and item response theory methods, as well as reliability and validity testing, to analyze cross-sectional data collected from women in two cities: Tiruchirappalli, India (N = 996) and Kampala, Uganda (N = 1,024). Through rigorous evaluation of conceptually grounded question (item) sets, we identify a set of valid, comprehensive scales. The Agency, Resources, and Institutional Structures for Sanitation-related Empowerment (ARISE) scales represent 16 sub-domains of sanitation-related empowerment, each of which can be used alone or in combination with others, as needed. The ARISE scales are the only set of psychometrically validated metrics for the measurement of women’s empowerment in WASH. In addition to the scales, we provide six indices to assess women’s direct experiences with sub-domains of sanitation-related empowerment, as well as validated item sets related to menstruation, which are available as optional add-on measures for those who menstruate. The ARISE scales and associated survey modules respond to an established need for an increased focus on empowerment in WASH. We provide researchers and implementers with tools to measure sub-constructs of empowerment in a valid and reliable way, to generate data for better targeting, design, implementation, and evaluation of strategies to improve women’s empowerment in the context of urban sanitation at the program and policy level.

## Highlights

• We developed quantitative survey instruments to measure sub-domains of women’s empowerment in relation to urban sanitation.
• We deployed the instruments in Tiruchirappalli, India and Kampala, Uganda and assessed reliability, validity, and measurement properties.
• The instruments demonstrate clear dimensionality, strong psychometric properties, good internal consistency, and multiple forms of validity.
• The Agency, Resources, and Institutional Structures for Sanitation-related Empowerment (ARISE) scales are valid and reliable instruments.
• We are continuing to refine six of the 16 scales and are testing all scales in new settings across South Asia and Sub-Saharan Africa.

## 1 Introduction

Water, sanitation and hygiene (WASH) programs in low- and middle-income countries (LMICs) have historically targeted women as instrumental in the achievement of program objectives, though there is growing recognition of the role of WASH in positively improving the life outcomes of women (Amebelu et al., 2021, Fisher et al., 2017). Some WASH programs also incorporate gender-sensitive approaches, with the aim of benefiting and empowering women. While attention to gender in WASH is not new (Fisher, et al., 2017), there has been a particular growth in WASH research that engages empowerment and related domains since 2015 (Caruso, et al., 2022). This growth may have been propelled by Sustainable Development Goal 6 (SDG6), which aims to ensure access to water and sanitation for all, and in which Target 6.2 includes language on “paying special attention to the needs of women and girls.” Even if lacking an explicit gender focus, programs that transfer information and other resources to women may contribute to women’s empowerment but may not measure this outcome. A lack of data on empowerment, in turn, limits the potential of WASH programs and policies to fully understand how they may be impacting health and development outcomes related to women.

To measure empowerment, a clear conceptualization and definition of empowerment is a necessary first step (Richardson, 2018b). Globally, the most common definition of empowerment is that of Kabeer, which is “the expansion in people’s ability to make strategic life choices in a context where this ability was previously denied to them” (Kabeer, 1999). Kabeer further conceptualized empowerment as having three dimensions: *resources*, or the human, material, and social pre-conditions to exercising choice; *agency*, which is “the ability to define one’s goals and act upon them,” and *achievements*, which are the possible outcomes of exercising agency (Kabeer, 1999). More recently, a framework developed by van Eerdewijk et al. and adopted by the Bill & Melinda Gates Foundation (BMGF) for their work across sectors conceptualizes empowerment slightly differently, including the domains of resources, agency, and institutional structures (each with several sub-domains) and specifically noting empowerment as both a process and an outcome (van Eerdewijk, et al., 2017). However, empowerment has been defined and conceptualized in many ways over time and across development sectors (Narayan-Parker, 2005).

Definitions and conceptualizations of empowerment have been operationalized through a number of measurement instruments. For example, in the agriculture sector, the Women’s Empowerment in Agriculture Index (WEAI) aims to measure agency in the agricultural context and has twelve indicators covering three sub-constructs of agency: intrinsic (power within), instrumental (power to), and collective agency (power with) (Malapit, et al., 2019). Measures inspired by the WEAI have been developed in other sectors, including the Women’s Empowerment in Livestock Index (WELI), the Women’s Empowerment in Nutrition Index (WENI), and Empowerment in WASH Index (EWI) (Dickin et al., 2021, Galiè et al., 2019, Narayanan et al., 2019). At the same time, other researchers have developed their own measures and indicators related to empowerment. A systematic review of women's empowerment and child nutrition found over 200 empowerment indicators across the studies included in the review. The authors of the review noted that even when the same dimension of empowerment was assessed, differences in measurement tools inhibited comparisons between studies (Santoso, et al., 2019).

Beyond the proliferation of instruments limiting comparability, another challenge is the lack of rigorous validation of tools used to measure empowerment. In the context of measurement instruments, validity is defined as “a judgment or statistical estimate based on accumulated evidence of how well scores on a test or instrument measure what they are supposed to measure” (Price, 2016). Demonstrating validity is essential for instruments that are intended to measure latent constructs and sub-constructs, which cannot be observed or measured directly. Best practices in validation include a series of steps, many involving advanced statistical analysis methods (Boateng, Neilands, Frongillo, Melgar-Quiñonez, & Young, 2018). Each validation step contributes a different type of evidence, including substantive (e.g. local relevance of the construct); structural (e.g., dimensionality of the construct); and external (e.g., associations with other scales that measure related constructs) evidence (Flake, Pek, & Hehman, 2017). All three types of evidence are needed for comprehensive scale evaluation (Flake, et al., 2017). While some instruments that aim to measure empowerment have undergone scale evaluation to this gold standard level, many others have not (Yount, et al., 2019). For this reason, researchers have urged more rigorous approaches to the quantitative measurement of empowerment (Richardson, 2018a, Richardson, 2018b, Yount et al., 2018). Specific recommendations include combining theory with analytic approaches such as factor analysis that are appropriate for complex, multidimensional constructs (Richardson, 2018b).

While sectors like nutrition have long included a focus on women’s empowerment (as evidenced by the large volume of measures identified in the systematic review described above), a similar focus has been lacking in the WASH sector (Caruso et al., 2022, Caruso and Sinharoy, 2019, Sinharoy and Caruso, 2019). Evidence from a systematic review conducted by our group indicates that WASH research has a very limited engagement with the concept of empowerment (Caruso et al., 2022). Specifically, of 257 articles included in the systematic review, all discussed empowerment or one of the sub-domains of empowerment to some extent, but only 17 (7 %) provided a definition of empowerment and/or examined how study populations conceptualized empowerment in their own contexts (Caruso et al., 2022). The lack of integration of definitions and theory in these WASH studies suggests that improved conceptualization and measurement of empowerment is needed in WASH. Further, empowerment should be measured both as an outcome and as a mediator and a mechanism, as some WASH programs seek to achieve health outcomes via interventions that enable individual- and household-level change through empowerment.

Several specific needs exist related to the measurement of women’s empowerment in WASH. First, there is a need for rigorously validated tools to measure empowerment. Validated tools are required to enable the design, targeting, monitoring, and evaluation of programs that seek to enhance empowerment. To our knowledge, the EWI is the only tool that has been developed to measure empowerment in WASH, and it has been pilot tested but has not yet been rigorously validated (Dickin, et al., 2021). Second, there is a need for tools that have been validated across settings and contexts. Certainly, due to contextual differences in empowerment, site-specific tools can be useful (Desai, Chen, Reddy, & McLaughlin, 2022). However, highly contextualized tools may be limited in their scalability, generalizability, and cross-cultural equivalence. Tools that have been validated across settings are needed for comparative analysis and global monitoring (Desai, et al., 2022). Third, there is a need for tools with demonstrated internal consistency (meaning that items are highly correlated) and temporal stability (DeVellis, 2017c). Finally, there is a need for tools that consider multiple domains and sub-domains of empowerment to allow practitioners and researchers to comprehensively address the multi-dimensional facets of empowerment.

**Fig. 1:**
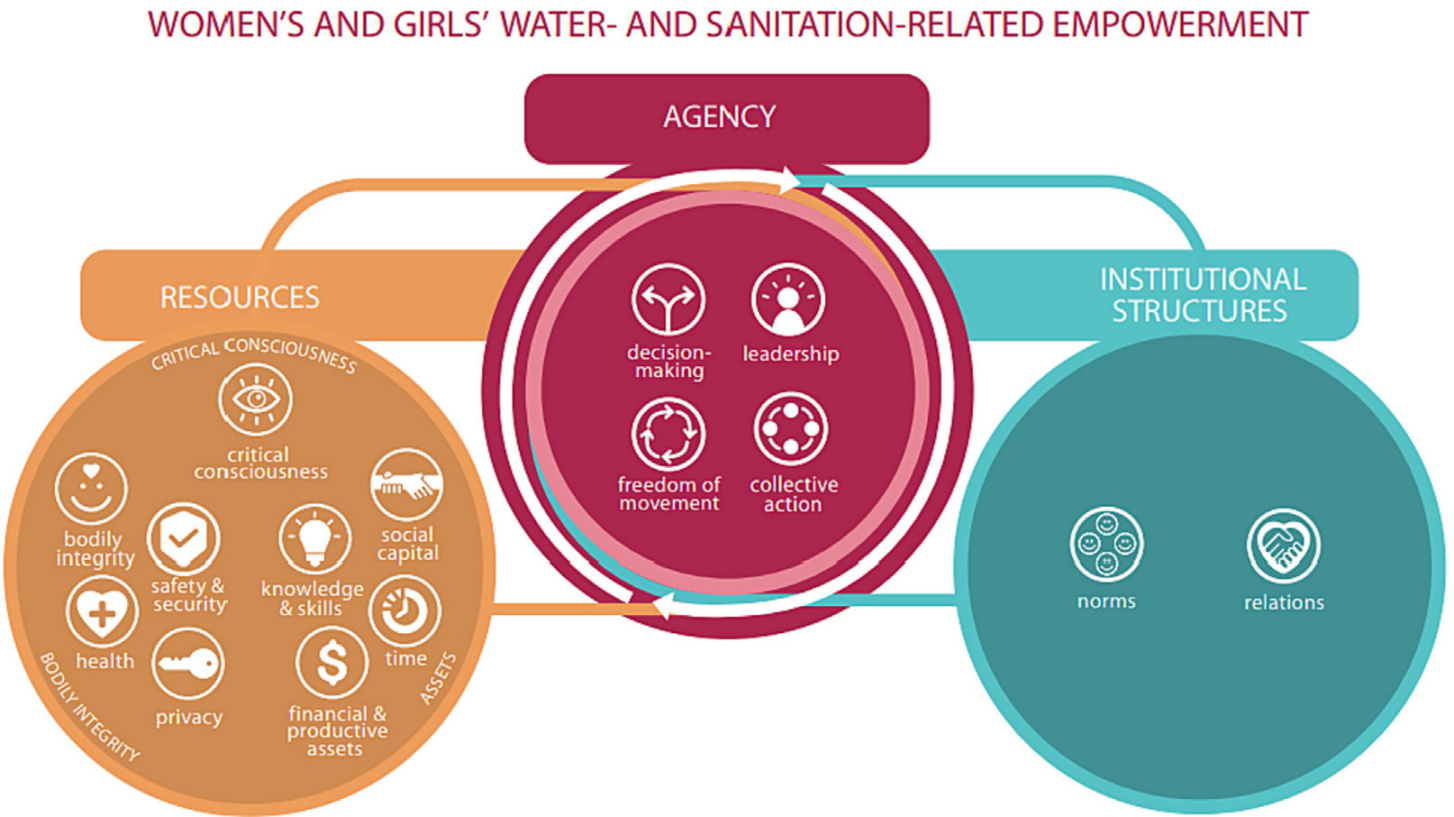
Conceptual framework of women’s sanitation-related empowerment.

To address prevailing measurement limitations, the objective of this study was to leverage an existing framework for the development and validation of instruments to measure women’s empowerment in the context of urban sanitation. We used the conceptual framework that was originally developed by van Eerdewijk et al. (van Eerdewijk, et al., 2017) and subsequently adapted to be sanitation-specific based on our systematic review (Figure 1) (Caruso et al., 2022). The framework includes three domains and 15 sub-domains of empowerment, which are distinct but interrelated. Sanitation-specific definitions for each sub-domain of empowerment have been provided in the study protocol and in Table 1 (Sinharoy, Conrad, Patrick, McManus, & Caruso, 2022). We developed survey instruments and collected data in two LMIC settings: Tiruchirappalli, India and Kampala, Uganda. We then employed rigorous analytic methods to assess the measurement properties of survey questions (item sets) that we used to operationalize each sub-domain of empowerment from our framework. Here we report the validation of the Agency, Resources, and Institutional Structures for sanitation-related Empowerment (ARISE) survey instruments, using data from Tiruchirappalli, India and Kampala, Uganda.

**Table 1:** Sanitation-specific definitions for sub-domains of empowerment, by domain (Sinharoy, Conrad, Patrick, McManus, & Caruso, 2022).

Sub-domain	Sanitation-Specific Definition
Resources	
Bodily Integrity	Women’s control over their bodies and ability to access and use their preferred sanitation location.
Health	Women’s complete physical, mental, and social well-being as affected by sanitation options and conditions; not merely the absence of disease or infirmity.^12^
Safety and Security	Women’s freedom from acts or threats of violence (physical or sexual), coercion, harassment, or force when accessing and using sanitation locations or engaging in sanitation-related decision-making processes in the public sphere.
Privacy	Women’s ability to maintain desired levels of privacy when accessing and utilizing sanitation locations.
Critical Consciousness	Women’s ability to identify and question how inequalities in power operate in their lives in relation to sanitation access and decision-making processes, and to assert and affirm their self-efficacy inside and outside of the household as it relates to sanitation.
Financial and Productive Assets	Women’s control over economic resources and long-term stocks of value such as land, for the purposes of meeting individual and household sanitation needs.
Time	Women’s control over their time and labor spent on sanitation-related tasks and activities.
Social Capital	Women’s relations and social networks that provide tangible and intangible value and support, including those that enable them to complete sanitation-related tasks and activities.
Knowledge and Skills	Women’s knowledge and skills related to sanitation (e.g. operation and maintenance of sanitation facilities) and their abilities to apply those knowledge and skills.
Agency	
Decision-Making	Women influence and make decisions about sanitation inside and outside the home.
Leadership	Women assume leadership positions, effectively participate, and support women’s leadership in informal and formal sanitation initiatives and organizations.
Collective Action	Women gain solidarity and take action collectively on sanitation-related issues.
Freedom of movement	Women have the autonomy to move freely to access sanitation facilities, collect water for sanitation-related needs, and/or attend forums on sanitation issues, and women have freedom of movement despite sanitation circumstances.
Institutional Structures	
Norms	Collectively held expectations and beliefs of how women and men should behave and interact inside and outside the household, specifically with regard to (a) the division of labor; (b) decision-making; (c) leadership; (d) collective action; and (e) freedom of movement.
Relations	The interactions and relations – including conflicts, support, hostility, and communication – with key actors that shape women’s sanitation-related experiences.

## 2 Methods

### 2.1 Study design

Details on study design have been described in the study protocol (Sinharoy, Conrad, Patrick, McManus, & Caruso, 2022). Briefly, the study involves three phases: item development; scale development and initial validation; and scale evaluation and further validation. Phase 1 included domain specification, a systematic review of peer-reviewed literature, a landscape analysis of peer-reviewed and grey literature, item generation, face validity and content validity assessment (through cognitive interviews, key informant interviews, and expert review), and item refinement. Phase 2 involved a second round of face validity and content validity assessment (through cognitive interviews), followed by survey implementation in two cities (Tiruchirappalli, India and Kampala, Uganda) and data analysis. Phase 3 will involve a final round of face validity and content validity assessment, followed by survey implementation in six additional cities (Narsapur and Warangal, India; Lusaka, Zambia; and Dakar, Senegal) and statistical analysis for further validation. This paper reports on the results of the quantitative data analysis from Phase 2.

### 2.2 Participants and procedures

Data were collected in two cities, Tiruchirappalli, India and Kampala, Uganda. These cities were selected purposively in conjunction with the funder (BMGF) from cities participating in the BMGF-funded Citywide Inclusive Sanitation (CWIS) program. We purposively selected 23 neighborhoods in Tiruchirappalli and 10 parishes in Kampala for survey administration in coordination with CWIS implementing partners and local government officials, with a focus on low- to middle-income neighborhoods. We then used random sampling procedures to select households within each neighborhood or parish and targeted an adult woman within each selected household. Inclusion criteria for the surveys were being a woman aged 18 or older who spoke Tamil (in India) or English or Luganda (in Uganda), who was mentally competent, was a full-time resident of the household (not a visitor) and had no hearing or speech impediments that would prevent comprehension or participation. Additional details have been described in the study protocol (Sinharoy, Conrad, Patrick, McManus, & Caruso, 2022).

We conducted surveys with 996 women in Tiruchirappalli and 1,024 women in Kampala from December 2019-January 2020. To assess test–retest reliability, we re-surveyed 73 participants in Kampala and 85 participants in Tiruchirappalli who agreed to respond to the same survey a second time within four weeks. Survey instruments were translated and independently back-translated into Tamil (in India) and Luganda (in Uganda). Surveys were programmed on tablets with Ona software. To mitigate potential effects of participant fatigue due to the length of the survey, the three survey sections pertaining to each domain of empowerment (resources, agency, institutional structures) were programmed to be administered in random order.

Enumerators, who were all women and fluent in the local language(s), participated in five days of training covering details of the survey, research ethics, and logistics. A pilot test was carried out on the first day of survey implementation in each site, with enumerators piloting the survey with one participant each and spot checks conducted by field-based supervisors. Data collection was supervised by at least one city coordinator and/or field supervisor per city.

### 2.3 Data collection instruments

The survey instrument included sections on demographics, water and sanitation access and behaviors, menstruation, each sub-domain of empowerment, and measures to assess validity of the scales.

The sections on empowerment included 15 scales, designed to measure 15 sub-domains within the three domains of empowerment in the conceptual framework: decision-making, leadership, collective action, and freedom of movement within agency; bodily integrity, safety and security, health, privacy, critical consciousness, financial and productive assets, time, knowledge and skills, and social capital within resources; and norms and relations within institutional structures (see Table 1 for definitions). All scales had ordinal, Likert-type response options.

The instruments also included measures to assess construct, criterion, and known groups validity of the 15 scales. These included six newly created indices to be used for construct validation. Indices were designed to measure women’s own experiences with household-level decision-making, community-level decision-making, leadership, collective action, and freedom of movement; given the sensitivity of asking about women’s direct experiences of violence, the index related to safety and security was designed to measure women’s awareness of other women’s experiences of sanitation-related violence while accessing sanitation. We were not able to assess validity for the Leadership scale or for the ‘awareness of inequalities related to sanitation’ sub-construct of Critical Consciousness due to a lack of existing appropriate validation measures. Measures that were included for assessment of validity are shown in Supplemental Table A.

### 2.4 Statistical analysis

**Fig. 2:**
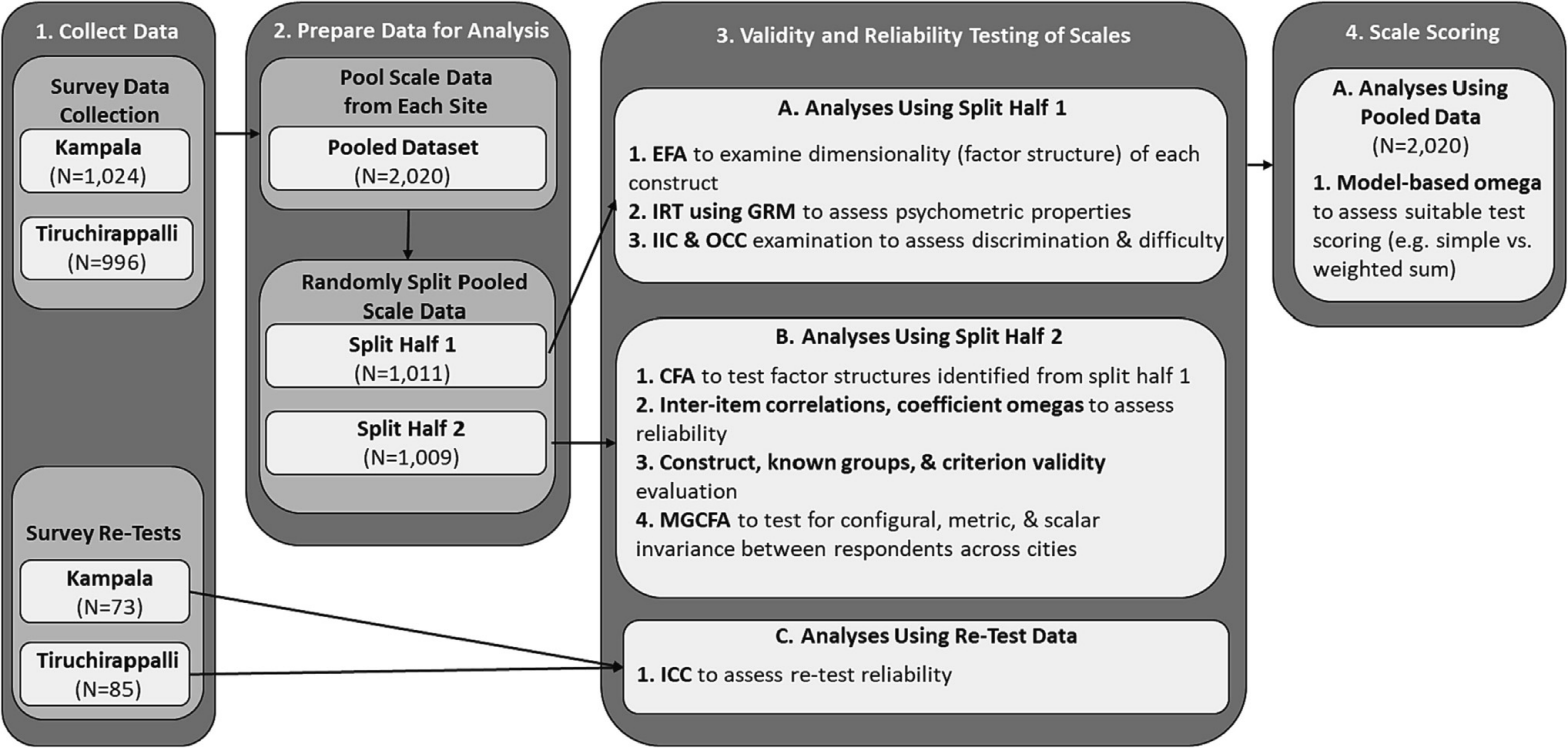
Multi-step analysis plan for assessment of reliability and validity of the ARISE scales.

Statistical analyses followed a sequenced, multi-step, a priori analysis plan, summarized below in Figure 2, to evaluate the measurement properties of the ARISE scales (Sinharoy, Conrad, Patrick, McManus, & Caruso, 2022). As shown in Figure 2, following data collection (Step 1) and data preparation and management (Step 2), we began with exploratory factor analysis (EFA) (Step 3.A.1), which is recommended as the first step for determining whether a set of survey items is tapping one or more theoretically meaningful latent constructs as intended (Bandalos and Finney, 2010, DeVellis, 2017a). EFA also facilitates the identification of individual survey items that are performing better or worse, for item reduction (DeVellis, 2017a). While EFA is often followed immediately by confirmatory factor analysis (CFA), we next used item response theory (IRT) approaches (Steps 3.A.2–3) to further test item performance, to ensure that we retained only those items that best measured the latent construct of interest (from both a theoretical and empirical perspective) (Boateng, et al., 2018). We then used CFA (Step 3.B.A) on the reduced item sets (scales) to test the factor structure (DeVellis, 2017a). After determining the final set of survey items to be retained for each scale, we proceeded to test the internal consistency (reliability) and the construct, known groups, and criterion validity of each scale (Steps 3.B.2–3) (DeVellis, 2017c, DeVellis, 2017d). Separately, we also assessed test–retest reliability (Step 3.C.1), or the ability of our instrument to measure the same constructs comparably over time (DeVellis, 2017c). We then tested for measurement invariance, to assess whether responses to survey items are comparable across populations. The final analysis for test scoring (Step 4) was done to determine whether the scale scores can be calculated using a simple sum score or whether a weighted score would be needed (McDonald, 2013). Each step is described in more detail below.

We began with pooled data from the two sites (N = 2020). We calculated univariate statistics on variables related to demographic characteristics, water and sanitation access and behaviors, and for indices and scales, by site. We assessed item-level distributions and extent of missingness, then estimated polychoric correlations for items within each scale. Using the pooled data for all 15 scales, we created two random-split half samples for use in subsequent analyses.

Using EFA with the first random split-half sample (N = 1011), we examined the dimensionality of the constructs being measured. We ran sequential EFA models separately for each scale, with the number of factors extracted per scale being determined based on analytical (e.g. parallel analysis, scree plot) and theoretical considerations. Each EFA model used means- and variance‐adjusted weighted least squares estimators and quartimin oblique rotation (Bandalos and Finney, 2010, DeVellis, 2017a). We made decisions to keep or remove items based on theoretical and statistical considerations. Specifically, in cases where variables captured an important aspect of the construct being measured, we followed recommendations to consider retaining those variables, and we further assessed their performance in subsequent analyses (Bandalos and Finney, 2010, Flora and Flake, 2017). Statistical criteria for removing items were pattern coefficients <|0.300|, high multidimensionality (i.e. cross-loadings (>|0.300|) on two or more factors with a difference between loadings of < 0.20), or significant negative pattern coefficients (Bandalos & Finney, 2010). We also removed items from the scales if they loaded alone on a factor; in some cases, we retained these as standalone items elsewhere in the survey instrument. We assessed model fit based on the following indices: Root Mean Squared Error of Approximation (RMSEA), Comparative Fit Index (CFI), Tucker-Lewis Index (TLI), and Standardized Root Mean Squared Residual (SRMR). RMSEA < 0.08, CFI > 0.95, TLI > 0.95, and SRMR < 0.06 are considered good fit, with RMSEA taking precedence over SRMR due to its better accuracy with ordinal data (Hooper et al., 2008, Shi et al., 2020).

Following EFA, we used IRT approaches to further assess the psychometric properties of the items that had been retained (Toland, 2014). The IRT analysis was carried out on the same random split-half sample from the EFA. We used graded response models (GRM), which are a type of IRT model for polytomous data, specifically for items with ordinal response options (Toland, 2014). We evaluated the assumptions of local independence and functional form, and assessed model-data fit graphically and statistically (Foster et al., 2017, Toland, 2014). We calculated discrimination and difficulty parameter estimates and visually examined item information curves (IIC) and option characteristic curves (OCC), also known in GRMs as item response category characteristic curves, to assess item performance (Desjardins and Bulut, 2018, DeVellis, 2017b, Finch and French, 2015, Toland, 2014). Very difficult items contribute little information on individuals with low levels of the latent trait being measured by each scale, while very easy items contribute little information about individuals with higher levels of the latent trait. Therefore, items that had low discrimination parameters or contributed low information were considered for removal. Since items on menstruation would not be applicable to all women, we split the menstruation-related items from the scales after assessing item-level properties and conducted subsequent analyses separately.

We used CFA on the remaining random split-half sample (N = 1009) to test the factor structures that were identified through the above process (Bandalos & Finney, 2010). For the scales, the factor loadings for all items in each factor were unconstrained and freely estimated, and we allowed for correlations between factors. In contrast, for menstruation-related factors, the factor loadings for the first indicator in each factor were fixed to 1.0, with the highest loading factors from the EFA specified as the marker variable. Factor indicators were fixed in order to ensure model identification with a small number of indicators in the menstruation-related factors. We used the same criteria and fit indices described above to make decisions to keep or remove items and to assess model fit.

To assess scale reliability, we used the same confirmatory random split-half sample and examined inter-item correlations and calculated coefficient omega as a measure of internal consistency (Hayes and Coutts, 2020, Trizano-Hermosilla and Alvarado, 2016). While consensus is lacking on thresholds for values of omega, we determined that values greater than or equal to 0.70 would be acceptable (Kelley and Pornprasertmanit, 2016, Kline, 2015). We then assessed test–retest reliability using data from the sub-sample of respondents who completed the survey twice within a four-week period (N = 158). We calculated intraclass correlation coefficients (ICCs) of scored scales with two-way mixed effect models of absolute agreement of the mean of k items (Koo & Li, 2016). We used the following reference values as thresholds for test–retest reliability: < 0.40, Poor; 0.50–0.75, Fair to Good; > 0.75, Excellent (Fleiss, 2011).

Construct validity (including known-groups validity) and criterion validity were evaluated for all scales where internal indices or external validated instruments were available (Supplemental Table A). Construct validity relates to whether a scale demonstrates an empirical relationship with another variable as would be hypothesized based on theory, whereas criterion validity requires empirical associations between a measure and another variable regardless of theory (DeVellis, 2017d). Specifically, we assessed construct and known groups validity for the scales for which we were able to identify and include an existing index and/or survey questions that were relevant to the sub-domain. We assessed criterion validity for the scales for which we were able to find and include an existing published/validated external scale. All validity analyses were done using the confirmatory random split-half sample. We assessed construct validity and external criterion validity using nonparametric Spearman rank correlations and generalized linear regression. We used t-tests and ANOVA to test for known-groups validity and examined inter-item and item-scale correlations.

We tested for measurement invariance at both the item and group level, again using the confirmatory random split-half sample. We used multiple-group CFA (MGCFA) to test for configural, metric, and scalar invariance between respondents in India and Uganda (Dimitrov, 2014). For each model, we assessed invariance based on changes in CFI greater than Δ0.01, RMSEA greater than Δ0.015, and SRMR greater than Δ0.03, with CFI taking precedence over RMSEA and SRMR as a criterion (Chen, 2007, Putnick and Bornstein, 2016). We then used IRT to test for uniform and non-uniform differential item functioning (DIF) and compared results from the MGCFA and IRT analyses (Edwards and Edelen, 2009, Finch and French, 2015).

To determine test scores, we calculated model-based omega in the pooled sample, to assess whether the formula test score should be calculated as a simple sum, a weighted sum, or some other calculation from item scores (e.g. a nonlinear function of items) (McDonald, 2013). Finally, we calculated Pearson correlations between scored scales to confirm that the 16 scales were distinct and not redundant.

Descriptive statistics were calculated using SAS v9.4 (SAS Institute, Cary NC, USA). All other analyses were done using MPlus v8.4 (Muthén & Muthén, Los Angeles CA, USA) and R v4.0 (R Core Team, Vienna, Austria).

### 2.5 Ethics

All participants provided oral (India) or written (Uganda) consent to enumerators in their local language using a standardized script. Participants in Uganda received UGX 10,000 (∼2.71 USD) in accordance with local policies and ethical requirements. Study activities were reviewed and approved by Internal Review Boards (IRBs) at Emory University (USA; IRB 00110271), Azim Premji University (India; Ref. No. 2019/SOD/Faculty/5.1), and Makerere University (Uganda; Ref. No. 2019–038). The funder was involved in identifying the conceptual framework and selecting cities for data collection. The funder had no role in data collection, data analysis, data interpretation, or writing of the manuscript.

## 3 Results

### 3.1 Descriptive statistics

Sociodemographic characteristics of the study population in each site are shown in Table 1. The average age in Kampala was 32 years and in Tiruchirappalli was 41 years. The majority of respondents in both cities were married and had completed primary school; other characteristics differed by city (Table 1). Descriptive statistics related to sanitation locations used by respondents for urination, defecation, and menstrual hygiene are shown in Supplemental Tables B, C, and D, respectively. Descriptive statistics for responses to individual items related to empowerment, including items related to menstruation, are shown by empowerment domain in Supplemental Tables E, F, and G.

### 3.2 EFA results

EFA results for each scale indicated models with a range of two to eight factors, as shown in Table 2. A total of 66 items were dropped based on results of the EFA. Items were dropped due to low pattern coefficients, low communality (i.e., correlation with other items), high multidimensionality, and/or poor conceptual fit with other items or with the underlying theoretical construct. A full list of individual items that were dropped and reasons for dropping is provided by domain in Supplemental Table H. Fit was acceptable for the final EFA models (Table 2).

**Table 2:** Sociodemographic characteristics of study participants in Kampala, Uganda and Tiruchirappalli, India.

**Characteristics**	**Kampala** (N = 1024)	**Tiruchirappalli** (N = 996)
**Age,** mean (sd)	31.82 (10.66)	40.81 (15.03)
**Marital status**		
Single, never married	181 (17.7 %)	105 (10.5 %)
Married	463 (45.2 %)	743 (74.6 %)
Unmarried, living with a partner	221 (21.6 %)	3 (0.3 %)
Widowed	52 (5.1 %)	122 (12.2 %)
Divorced/separated	105 (10.3 %)	23 (2.3 %)
**Education**		
Less than primary	187 (19.5 %)	90 (10.7 %)
Completed primary	728 (75.8 %)	642 (76.2 %)
Completed secondary	40 (4.2 %)	77 (9.1 %)
Higher than secondary	5 (0.5 %)	33 (3.9 %)
**Household composition**		
Household size, mean (sd)	4.48 (2.2)	4.28 (2.95)
Respondent has child(ren) < 5 years old	619 (60.4 %)	245 (24.6 %)
**Place of Birth**		
In this city	166 (16.2 %)	738 (74.1 %)
Elsewhere in this country	849 (82.9 %)	250 (25.1 %)
Outside this country	9 (0.9 %)	8 (0.8 %)
**Type of housing**		
Single family home	266 (26.0 %)	541 (54.3 %)
Apartment	62 (6.1 %)	251 (25.2 %)
Compound with shared living spaces	664 (64.8 %)	118 (11.8 %)
Other	12 (1.2 %)	6 (0.6 %)
**Income generating activities**		
Earns an income	618 (60.4 %)	273 (27.4 %)
Does not earn an income	406 (39.6 %)	721 (72.4 %)
**Socioeconomic Status: Wealth Quintiles**		
Lowest	248 (24.2 %)	165 (16.6 %)
Second	104 (10.2 %)	219 (22.0 %)
Middle	235 (22.9 %)	221 (22.2 %)
Fourth	207 (20.2 %)	180 (18.1 %)
Highest	230 (22.5 %)	211 (21.2 %)

### 3.3 IRT results

We conducted IRT analyses using all items that had been retained in the final EFA models. A total of 12 items were dropped based on results of the IRT analysis, typically because they contributed low information or had low or negative discrimination (Supplemental Table H). All other items across all scales had adequate discrimination and contributed higher levels of information. Item information curves for each scale can be found in Supplemental Figures A-P.

### 3.4 CFA results

Following EFA and IRT, we carried out CFA for all scales. An additional 28 items were dropped following CFA due to low loadings, substantial correlations with covariance and covariances of other items, to improve model fit statistics, and for conceptual and theoretical reasons (Supplemental Table H). As described above, we conducted separate CFA analyses for menstruation-related factors that were removed from the scales but that are available as optional add-ons. Fit statistics for the CFA models with menstruation-related factors demonstrated good fit; results are shown by sub-domain in Supplemental Table I.

A further decision was made to split the Critical Consciousness scale into two scales representing self-efficacy and awareness of inequalities related to sanitation. Our operational definition of Critical Consciousness had two components (Table 1), which were reflected in empirical evidence indicating that the scale was measuring two distinct concepts. Specifically, in both EFA and CFA, items representing self-efficacy loaded cleanly onto two factors and items representing awareness of inequalities related to sanitation loaded cleanly onto two separate factors. In CFA, the self-efficacy factors were correlated to each other, and the awareness of inequalities factors were correlated to each other, but factors of each conceptual area were not highly correlated to factors of the other conceptual area (r < 0.20). This decision brought the total number of scales to 16.

Based on the modification indices and discussions within the team about potential relationships between items in the hypothesized scale model structures, we added residual covariances between items to 11 of the 16 scales. Fit statistics for the final CFA models demonstrated good overall fit and are shown in Table 3.

**Table 3:** Results from final exploratory factor analysis (EFA) models for each scale.

**Scale Name**	**# of Factors**	**Range of Pattern Coefficients**	**RMSEA (90 % CI)**	**SRMR**	**CFI**	**TLI**
**Resources**						
Health	6	0.39–0.958	0.022 (0.012–0.030)	0.015	0.998	0.996
Bodily integrity	4	0.492–0.951	0.064 (0.058–0.070)	0.046	0.987	0.977
Safety and security	6	0.37–0.959	0.054 (0.049–0.059)	0.022	0.993	0.987
Privacy	2	0.659–0.984	0.072 (0.063–0.081)	0.038	0.985	0.976
Financial and productive assets	5	0.306–0.975	0.077 (0.069–0.085)	0.032	0.990	0.976
Social capital	4	0.477–0.858	0.123 (0.116–0.131)	0.036	0.954	0.905
Time	3	0.504–1.001	0.081 (0.070–0.092)	0.017	0.993	0.986
Knowledge	5	0.482–0.861	0.089 (0.081–0.098)	0.022	0.977	0.939
Critical consciousness	5	−0.309–0.945	0.092 (0.085–0.099)	0.040	0.969	0.931
**Agency**						
Leadership	2	0.596–0.999	0.038 (0.016–0.061)	0.033	0.999	0.997
Decision making	6	0.301–0.961	0.094 (0.088–0.100)	0.015	0.986	0.968
Collective action	3	0.563–1.006	0.180 (0.164–0.195)	0.023	0.986	0.959
Freedom of movement	2	0.715–0.986	0.147 (0.132–0.162)	0.139	0.987	0.971
**Institutional structures**						
Norms	8	−0.64–0.95	0.059 (0.054–0.064)	0.020	0.986	0.969
Relations	5	0.311–0.963	0.114 (0.109–0.120)	0.036	0.967	0.938

Through the EFA, IRT, and CFA process, we determined that some items should be revised for clarity and conceptual alignment with the constructs being measured. For example, to capture the overall sanitation experience, we revised questions that asked about sanitation access “while at home” and “while away from home” to focus on sanitation access in general. We revised five items in two scales (Leadership and Social Capital). In addition, we identified a need to add new items to fill conceptual gaps. Therefore, we developed 13 new items across four scales (Health, Bodily Integrity, Safety and Security, and Privacy), which are currently being tested as part of ongoing scale evaluation.

### 3.5 Reliability

To assess internal consistency, we examined inter-item correlations and calculated the reliability coefficient, coefficient omega (*ω*), for each factor identified from the CFA. As described above, we used a threshold of ≥ 0.70 as acceptable for *ω*. Of the 49 factors identified across the 16 scales, 42 had values of *ω* > 0.70 and seven had values of *ω* < 0.70. Of the latter category, six factors had moderate values>0.50 and<0.70. The remaining factor had a value of 0.45.

Test-retest reliability was analyzed for the sub-sample of surveys that were conducted twice with the same individuals in Kampala (N = 73) and Tiruchirappalli (N = 85). The analysis indicated that all scales had fair to good test–retest reliability, with the exception of Norms, Leadership, and the Critical Consciousness sub-scale measuring the awareness of inequalities, which had poor test–retest reliability. We then re-did the analysis, stratified by country, to identify whether the scales with poor test–retest reliability performed worse in one country than in the other. On average, results were better in India than in Uganda. Only the Safety and Security scale had poor test–retest reliability in India, while two scales (Health and Time) had excellent reliability, and the remaining scales had fair to good reliability. Conversely, there are several particularly problematic results in the Uganda sample, with only 6 scales (Health, Bodily Integrity, Safety and Security, Financial and Productive Assets, Social Capital, and Knowledge) having fair to good reliability, and the rest all having poor reliability (Table 4).

**Table 4:** Model fit statistics from confirmatory factor analysis (CFA).

**Scale Name**	**# of Factors**	**Range of Pattern Coefficients**	**RMSEA (90 % CI)**	**SRMR**	**CFI**	**TLI**
**Resources**						
Health	5	0.809–0.929	0.051 (0.045–0.057)	0.040	0.985	0.981
Bodily integrity	2	0.776–0.981	0.060 (0.047–0.073)	0.040	0.998	0.997
Safety and security	5	0.756–0.956	0.058 (0.053–0.063)	0.037	0.991	0.989
Privacy	1	0.737–0.909	0.038 (0.000–0.083)	0.006	1.000	0.998
Financial and productive assets	3	0.389–1.063	0.053 (0.038–0.069)	0.013	0.998	0.995
Social capital	2	0.538–0.899	0.048 (0.035–0.062)	0.017	0.995	0.993
Time	2	0.835–0.921	0.048 (0.027–0.071)	0.007	0.999	0.998
Knowledge	4	0.666–0.911	0.058 (0.047–0.069)	0.017	0.991	0.985
Critical consciousness (Scale 1)	2	0.480–1.166	0.023 (0.000–0.051)	0.009	1.000	0.999
Critical consciousness (Scale 2)	2	0.074–3.590	0.066 (0.046–0.088)	0.023	0.995	0.990
**Agency**						
Leadership	2	0.617–0.978	0.036 (0.018–0.053)	0.079	0.998	0.997
Decision making	5	0.752–0.941	0.059 (0.051–0.067)	0.018	0.995	0.993
Collective action	3	0.756–0.929	0.053 (0.037–0.069)	0.010	0.998	0.996
Freedom of movement	2	0.742–1.005	0.021 (0.000–0.056)	0.016	1.000	1.000
**Institutional structures**						
Norms	6	0.434–1.000	0.055 (0.050–0.059)	0.033	0.982	0.977
Relations	3	0.589–0.965	0.053 (0.044–0.062)	0.026	0.996	0.995

### 3.6 Validity

After assessing reliability, (Table 5) we assessed construct, criterion, and known-groups validity. For all analyses, we reverse-scored the scales for Health, Safety and Security, Privacy, Time, and Freedom of Movement, such that a higher score would indicate a higher level of empowerment in that sub-domain. Results of the validity assessments are shown in Supplementary Table J.

**Table 5:** Intraclass correlation coefficients for test–retest assessment of scored scales, for both countries combined and each country separately.

**Scale Name**	**Combined**	**India**	**Uganda**
**Resources**			
Health	0.641	0.748	0.495
Bodily integrity	0.616	0.544	0.552
Safety and security	0.488	0.382	0.519
Privacy	0.466	0.570	0.299
Financial and productive assets	0.661	0.636	0.593
Social capital	0.562	0.494	0.490
Time	0.562	0.767	0.236
Knowledge	0.620	0.649	0.603
Critical consciousness (Scale 1)	0.477	0.515	0.300
Critical consciousness (Scale 2)	0.368	0.518	0.161
**Agency**			
Leadership	0.346	0.482	0.038
Decision making	0.568	0.533	0.360
Collective action	0.596	0.570	0.357
Freedom of movement	0.457	0.565	0.215
**Institutional Structures**			
Norms	0.244	0.462	−0.568
Relations	0.403	0.440	0.236

### 3.7 Construct validity

We assessed construct validity for seven of the 16 scales. For six of the seven scales, we hypothesized a positive relationship, in which higher scale scores would be significantly correlated with higher scores on the measure being used for validation. The exception was for the Knowledge and Skills sub-domain, in which we hypothesized a negative correlation between the scale score and the three validation questions (because a higher score on the survey questions being used for validation would indicate less exposure to media and information). For all seven sub-domains, results indicated correlations in the expected directions.

### 3.8 Criterion validity

We assessed criterion validity for nine of the 16 scales. For seven of the nine scales, we hypothesized a positive relationship, in which higher scale scores would be significantly correlated with higher scores on the measure being used for validation. The two exceptions were for the Financial/Productive Assets and Norms sub-domains, in which we hypothesized a negative correlation between the scale score and the validation questions (because a higher score on the survey questions being used for validation would indicate less control over money and more restrictive gender norms, respectively). Results indicated significant correlations in the expected directions, except for the Leadership scale, for which the correlation was negative and not statistically significant.

### 3.9 Known groups validity

We assessed known groups validity for seven of the 16 scales. Unlike for construct and criterion validity, the known groups validity assessment involved testing for differential means in scale scores across response groups. All tests indicated significant differential means across response groups, as hypothesized. Additional details are provided in Supplementary Table J.

### 3.10 Measurement invariance

We tested invariance of measurement characteristics (including structure, loadings, and intercepts) of the factor model across the samples from India and Uganda using MGCFA. The configural invariance model had good fit, indicating that the factor structures (i.e. the number of factors and pattern of indicator-factor loadings) were invariant across countries, meaning that the items of the scales measure the same constructs in each group.

The assessment of metric invariance suggested a lack of equality of factor loadings for Privacy, Financial and Productive Assets, Social Capital, Knowledge, and both Critical Consciousness scales, as indicated by changes in both CFI and RMSEA that were larger than the recommended thresholds of Δ0.01 and Δ0.015, respectively. Therefore, the metric invariance model was rejected for these scales, indicating that the items in those scales may have different relationships to the underlying latent constructs being measured in each group. When comparing these scales across India and Uganda, the directionality of results can be compared with confidence; further comparisons of the magnitude of results should be done with caution. The Freedom of Movement, Relations, Time, Decision-Making, and Collective Action scales had changes in RMSEA that were beyond the recommended range, but changes in CFI remained within the acceptable range. Given that CFI is the main criterion for assessing invariance, we did not reject the metric invariance model for these scales. None of the scales had a change in SRMR that was larger than the recommended threshold of Δ0.03.

Having rejected the metric invariance model for Privacy, Financial and Productive Assets, Social Capital, Knowledge, and both Critical Consciousness scales, we also rejected the scalar invariance model for those scales. In addition, the Health, Safety and Security, Norms, Relations, Time, Decision-Making, and Collective Action scales had changes in SRMR that were larger than the recommended threshold for scalar invariance of Δ0.01. However, again, because CFI is the main criterion for invariance tests, we did not reject the scalar invariance model for these scales.

We also used IRT approaches to assess differential item functioning at the item level. The results for configural invariance were the same as in the MGCFA and indicated equivalent form across groups for all 16 scales. Results for metric invariance were also the same as in the MGCFA for Social Capital, Knowledge, and the first Critical Consciousness scale, indicating a lack of invariance in factor loadings for these three scales. In addition, the IRT methods suggested a lack of metric invariance for the Freedom of Movement, Leadership, Relations, and Time scales.

### 3.11 Scoring

Bifactor confirmatory factor analysis models were fit for each scale, apart from the single-factor Privacy scale as bifactor models require at least 2 underlying factors; all models had acceptable model fit. Scoring was examined using coefficient omega (*ω*) and the corresponding hierarchical coefficient omega (*ω_H_*) from the bifactor model (Rodriguez, Reise, & Haviland, 2016). As the *ω_H_*/*ω* ratio approaches 1, a total domain is favored, and *ω_H_* values greater than ω values provide further support for total scores rather than domain scores. Low values (<0.7) on both *ω* and *ω_H_* would indicate a need for empirically weighted scores. All scales demonstrated high *ω_H_* in bifactor models. The *ω_H_*/*ω* ratio approached 1 for all scales (range 0.96–1.01), supporting the use of unit-weighted total scores for all scales. These results indicate that all scales can be scored using a simple sum of responses to all items in the scale, and a weighted score is not needed. Results of the analyses for scoring are shown in Supplementary Table K.

Finally, correlations between scored scales were low to moderate, with the maximum observed correlation being 0.65. These results indicate that, while related, all scales were distinct. Higher correlations were seen between conceptually similar scales. A full correlation matrix is provided in Supplementary Table L.

## 4 Discussion

### 4.1 Summary and interpretation

This study aimed to develop and validate survey instruments to measure women’s empowerment in the context of urban sanitation. We used a rigorous, mixed-methods approach to develop and psychometrically evaluate item sets that were both conceptually grounded and contextually relevant. This process allowed us to identify a set of valid, comprehensive scales representing 16 sub-domains of sanitation-related empowerment, which can be used alone or in combination.

While other studies have proposed instruments to measure empowerment in WASH, ours is the first and only study, to our knowledge, to develop and empirically validate such an instrument using gold standard approaches. Specifically, other studies have not used factor analysis or IRT approaches for validation (Dickin, et al., 2021). These methods are recommended for the validation of measures of latent constructs such as empowerment because they allow researchers to establish, with a high degree of confidence, that the instrument in question is measuring the construct that it intends to measure (Boateng et al., 2018, Richardson, 2018b). Therefore, the results of our study provide unique contributions to the literature in the form of the first and only set of rigorously validated metrics for the measurement of sub-domains of sanitation-related empowerment. Our study also provides a rigorous example that others may follow when developing scales, including those that measure empowerment in WASH or other sectors, as well as other complex latent constructs.

### 4.2 Implications for research and practice

Our survey instruments can be used to inform the design, targeting, and evaluation of urban sanitation programs in several ways. For example, the scales can be used, alone or in combination, to inform program design by assessing baseline levels of empowerment by sub-domain and identifying specific sub-domains that may be strengthened through further intervention. Using individual scales alone will allow for targeted attention to specific sub-domains of interest, while using all scales together will allow for the comprehensive measurement of the multi-dimensional facets of empowerment. The scales can also be used throughout program implementation to allow implementers and researchers to examine pathways of change and/or bottlenecks preventing change during formal program evaluations. Researchers and practitioners may also use the item sets related to menstruation, which are available as optional measures for women who menstruate. We recommend that researchers and practitioners using the scales and optional menstruation factors in new locations conduct a CFA to test the factor structure and assess reliability and construct and criterion validity, in line with best practices (Bandalos and Finney, 2010, Boateng et al., 2018). When a full validation is not possible, we recommend assessing content validity of the scales by conducting cognitive interviews prior to full deployment.

Survey development and validation, when done rigorously, is a complex, multi-phased, and resource-intensive process (Boateng, et al., 2018). As noted above, this paper represents the second of three phases. Our group is continuing to refine and validate the ARISE scales by collecting data in additional cities, including in Warangal and Narsapur (India), Lusaka (Zambia), Meherpur and Saidpur (Bangladesh), and Dakar (Senegal), as well as collecting a second round of data in Kampala and Tiruchirappalli (Sinharoy, Conrad, Patrick, McManus, & Caruso, 2022). Ten of the sixteen scales (Financial and Productive Assets, Time, Knowledge, both Critical Consciousness scales, Collective Action, Decision-Making, Freedom of Movement, Norms, and Relations) have remained the same across Phase 2 and Phase 3. These ten scales, which are available in Supplementary Tables M−O, will require less testing, but all scales will undergo some assessment of reliability, validity, and measurement properties in these new settings. We have also developed short forms for the five scales that had >10 items (Norms, Relations, Safety and Security, Health, and Decision-Making) and are currently testing these in two cities. The short forms will offer more options for program implementers and researchers for program monitoring. Specifically, having shorter scales will allow for more frequent assessments with less burden, which in turn will allow for better targeting and more agile program implementation. Given the iterative nature of this process, we recommend that researchers embarking on scale development and validation studies of any latent construct plan for multiple rounds of data collection, to allow for careful testing and refinement of scales, and to ensure that the final product is as useful as possible.

## 5 Limitations

Although we employed a rigorous approach during the development, testing, and validation of our survey instruments, our study has limitations. First, the data used for the analyses presented here are limited to those generated in two cities. The validity of our results beyond these settings is unknown, though our survey instruments are currently being deployed in new locations for further validation. Second, we observed that one of the 49 factors had low reliability (internal consistency) as assessed by *ω*, and three scales (Norms, Leadership, and the ‘awareness of inequalities’ sub-scale of Critical Consciousness) had poor temporal (test–retest) reliability. We hypothesize that respondent fatigue may have contributed to some test–retest reliability values being lower than anticipated. We also note that participation in retests was voluntary and may reflect some selection bias. However, the analyses described here have resulted in all scales being shortened, with some being reduced in length by almost 50 %, and we plan to assess test–retest reliability again in the current phase of data collection and analysis. Third, we did not assess construct, external criterion, or known groups validity for the Leadership scale or for the ‘awareness of inequalities’ sub-scale of Critical Consciousness. Fourth, while the scales demonstrated configural invariance, the metric and scalar invariance models were rejected for six of the 16 scales (Privacy, Financial and Productive Assets, Social Capital, Knowledge and Skills, and both Critical Consciousness scales) based on MGCFA analyses. Metric invariance was also rejected for four other scales (Freedom of Movement, Leadership, Relations, and Time) based on IRT analyses. Despite these limitations, all scales demonstrated clear dimensionality, strong psychometric properties, and internal consistency, as well as construct, external criterion, and/or known groups validity.

## 6 Conclusion

In sum, through the analysis of data collected in Tiruchirappalli, India and Kampala, Uganda, we rigorously validated 16 scales to measure sub-domains of women’s sanitation-related empowerment, demonstrating through empirical evidence that each scale measures the latent constructs that it is intended to measure. The measurement scales generated through our study complement each other yet offer their own unique contributions for the comprehensive measurement of empowerment constructs and sub-constructs. As part of an iterative and ongoing scale evaluation process, several scales are being revised, and all scales are undergoing further evaluation in additional locations. Further psychometric testing of the ARISE scales is recommended, including in other settings and populations, to ensure their relevance and comparability across contexts. Given the critical importance of women’s empowerment for health and development, ongoing rigorous validation of instruments to measure empowerment is urgently needed. Such instruments can guide the development community’s agenda by contributing data for program design and evaluation as well as for policy recommendations regarding women’s empowerment and well-being.

## CRediT authorship contribution statement

**Sheela S. Sinharoy:** Conceptualization, Methodology, Resources, Writing – original draft, Supervision, Project administration, Writing – review & editing, Funding acquisition. **Shauna McManus:** Methodology, Formal analysis, Data curation, Writing – original draft, Writing – review & editing, Visualization. **Amelia Conrad:** Investigation, Writing – review & editing, Supervision. **Madeleine Patrick:** Investigation, Writing – review & editing, Supervision. **Bethany A. Caruso:** Conceptualization, Methodology, Resources, Writing – review & editing, Supervision, Project administration, Funding acquisition.

## Declaration of Competing Interest

The authors declare that they have no known competing financial interests or personal relationships that could have appeared to influence the work reported in this paper.

## Data Availability

Data will be made available on request.
