# Supplementary material for: The Agency, Resources, and Institutional Structures for Sanitation-related Empowerment (ARISE) Scales: Development and validation of measures of women’s empowerment in urban sanitation for low- and middle-income countries
Source: World Dev. 2023 Apr;164:106183. doi: 10.1016/j.worlddev.2023.106183 (PMC9918868; doi:10.1016/j.worlddev.2023.106183)
Supplement: Supplementary data 1 [file mmc1.docx]

**Supplemental Table A: Measures used for validation**

| **Scale Name** | **Questions / Measures for Validity** | **Type of Validity Assessed** | | |
| --- | --- | --- | --- | --- |
|  |  | **Construct** | **Criterion** | **Known Groups** |
| **Resources** |  |  |  |  |
| Bodily integrity | Survey questions on privacy of sanitation location: Is it possible for someone to see you while you are using this sanitation location? Is this sanitation location lockable from the inside? Is there sufficient room inside this sanitation location? Is this sanitation location located in a private place? |  |  | X |
| Health | WHO 5 Well-being Index (Topp, Østergaard, Søndergaard, & Bech, 2015) |  | X |  |
|  | Patient Health Questionnaire - 4 (Kroenke, Spitzer, Williams, & Löwe, 2009) |  | X |  |
|  | One item from the Patient-Reported Outcomes Measurement Information System (PROMIS) global health subscale (Pilkonis et al., 2014): In general, how would you rate your physical health? (Response options: Excellent, Very Good, Good, Fair, Poor) |  |  | X |
| Safety and security | Index of women’s awareness of other women’s experiences of sanitation-related violence while accessing sanitation: I know of a woman in this community who has been sexually assaulted by a man or boy when she went to a sanitation location. I know of a woman in this community who has been physically harmed by a man or boy when going to a sanitation location. I know a woman who has had someone expose himself or spy/peep on her when she went to a sanitation location. I know of a woman who has had someone make sexual comments or say obscene things to her when she went to a sanitation location. I know a woman who has experienced harassment, such as being called by rude names, yelling, or shaming, when she went to a sanitation location. | X |  |  |
| Privacy | Survey questions on privacy of sanitation location: Is it possible for someone to see you while you are using this sanitation location? Is this sanitation location lockable from the inside? Is there sufficient room inside this sanitation location? Is this sanitation location located in a private place? |  |  | X |
| Financial and   productive assets | Survey questions on asset ownership | X |  |  |
|  | One question from SEEP Network Assessment Tool for Microfinance Practitioners (Cohen, 2000): When you want or need to buy things like food or clothing for yourself or your family, which of the following answers best describes your situation?  You have your own money so you can usually buy what you need  You occasionally have to get the money from your husband or someone else in the household  You always have to get the money from your husband or someone else in the household |  | X | X |
| Social capital | Eight items from the 12-item Multidimensional Scale of Perceived Social Support (Response options: Completely disagree, Mildly disagree, Neither agree nor disagree, Mildly agree, Completely agree) (Dahlem, Zimet, & Walker, 1991) 1. My family really tries to help me. 2. I get the emotional help and support I need from my family. 3. My friends really try to help me. 4. I can count on my friends when things go wrong. 5. I can talk about my problems with my family. 6. I have friends with whom I can share my joys and sorrows. 7. My family is willing to help me make decisions. 8. I can talk about my problems with my friends. |  | X |  |
| Time | Survey questions on time spent on tasks related to sanitation: How many times a week do you have to go to the water source to collect water for sanitation needs? How long does it take for you to collect water? How many minutes do you need to walk to your sanitation location? | X |  |  |
| Knowledge | One item from the Demographic and Health Survey (DHS8) (ICF, 2021) on completed schooling:  Did you ever attend school? |  |  | X |
|  | Four items from the Demographic and Health Survey (DHS8) on access and use of media (ICF, 2021): Do you read a newspaper or magazine at least once a week, less than once a week, or not at all? Do you listen to the radio at least once a week, less than once a week, or not at all? Do you watch television at least once a week, less than once a week, or not at all?  Have you ever used the internet? | X |  | X |
| Critical consciousness   - Scale 1 | General Self-Efficacy - 6 Scale (Romppel et al., 2013) |  | X |  |
| Critical consciousness   - Scale 2 | None |  |  |  |
| **Agency** |  |  |  |  |
| Leadership | Index of women's assumption of and participation in formal and informal leadership positions:  Are you involved in any community group that addresses sanitation issues, such as construction, repairing, maintaining, or promoting the use of toilets or latrines? What does this group do? Do men and women participate in this group or only women? Do you have an elected or appointed leadership position, such as president, treasurer, or secretary, in any sanitation-focused committee or group in which you are involved? Do you feel you have influence over the decisions made by this group? Do you feel free to express your own opinion in the meetings and discussions of this group? Have you ever been pressured to support a position you did not agree with because a family member told you to? Have you ever supported a position you did not agree with because someone pressured you to? Do you serve as an informal leader for sanitation-related issues in this community, such as sanitation education, latrine promotion, or solving disputes? Do women in your community listen to your opinions and input on sanitation-related issues? Do men in your community listen to your opinions and input on sanitation-related issues? Do local leaders listen to your opinions and input on sanitation-related issues? | X |  |  |
|  | Socio-political Control Scale (Peterson et al., 2006)  Leadership competence factor (Response options: Strongly disagree, Disagree, Neither disagree nor agree, Agree, Strongly agree; Number 3 reverse scored for analysis): 1. I am often a leader in groups. 2. I would prefer to be a leader rather than a follower. 3. I would rather someone else took over the leadership role when I’m involved in a group project. |  | X |  |
| Decision making | Index documenting women influencing and making decision about sanitation in the home: I have been present when members of my household have discussed sanitation-related issues I have spoken up about sanitation-related issues in my household. Members of my household have listened to my opinions about sanitation issues. Members of my household have sought my input when making sanitation-related decisions.  I have influenced major decisions about sanitation in my household, such as construction or large repair projects I have influenced decisions about latrine/toilet repairs or enhancements in my household, like new floor tiles, doors, locks, or lights. I have influenced decisions about how my household cleans and maintains the sanitation environment/facility I have independently made major decisions about household sanitation, such as construction or large repair projects I have independently made decisions about household latrine/toilet repairs or enhancements, like new floor tiles, doors, locks, or lights. I have independently made decisions about small sanitation-related purchases, like soap, toilet paper, etc. I have independently made decisions about how my household cleans and maintains the sanitation environment/facility. | X |  |  |
|  | Index documenting women influencing and making decisions about sanitation outside of the home: I have attended a community meeting about sanitation issues. I have spoken up in a community meeting about sanitation issues. I have been listened to in a community meeting about sanitation issues. Local leaders, NGOs, or government officials have sought my input when making sanitation-related decisions. I have influenced decisions about sanitation for my community. By influencing a decision, we mean that your voice and opinion are considered and affect the final decision that is taken. I have made decisions about sanitation for my community. | X |  |  |
|  | Three items from the Demographic and Health Surveys (Response options: Respondent; Husband; Respondent and husband jointly; Someone else; Other) (ICF, 2021): 1. Who usually makes decisions about health care for yourself? 2. Who usually makes decisions about making major household purchases? 3. Who usually makes decisions about visits to your family or relatives? |  |  | X |
|  | Survey questions on general decision making (Response options: No, not at all comfortable; Yes, but with a great deal of difficulty; Yes, but with a little difficulty; Yes, fairly comfortable; Yes, very comfortable): 1. Do you feel comfortable expressing your opinion at a community meeting where only women are present? 2. Do you feel comfortable expressing your opinion at a community meeting where both men and women are present? | X |  |  |
| Collective action | Index of women's participation in collective action around sanitation: In the past year, have you gathered with members of your community to discuss sanitation-related problems and possible solutions? Was this group composed of both men and women or women only? In the past year, have you gathered with neighbors or others in your plot or compound to discuss sanitation-related problems and possible solutions? Was this group composed of both men and women or women only? In the past year, have you organized with members of your community to demand, construct, fund, or acquire land for latrines? Was this group composed of both men and women or women only? In the past year, have you organized with neighbors or others in your plot or compound to demand, construct, fund, or acquire land for latrines? Was this group composed of both men and women or women only? In the past year, have you joined with members of your community to make improvements to a sanitation location, such as toilets and latrines? Was this group composed of both men and women or women only? In the past year, have you joined with neighbors or others in your plot or compound to make improvements to a sanitation location, such as toilets and latrines? Was this group composed of both men and women or women only? In the past year, have you joined with members of your community to conduct trainings or awareness sessions related to sanitation? Was this group composed of both men and women or women only? In the past year, have you joined with members of your community to petition local leaders to make sanitation improvements? Was this group composed of both men and women or women only? In the past year, have you joined with members of your community to influence media to bring awareness and attention to sanitation problems in the community? Was this group composed of both men and women or women only? In the past year, have you joined with members of yoru community to participate in a protest/movement about sanitation issues? Was this group composed of both men and women or women only? | X |  |  |
|  | Community Capacity Instrument, Connections dimension (Response options: Strongly disagree, Disagree, Neither disagree nor agree, Agree, Strongly agree; Numbers 2 and 5 reverse scored for analysis): 1. Most people in the community can be trusted 2. I don't feel welcome to join local groups and activities 3. Residents are friendly and inclusive of newcomers 5. I have little in common with most people who live here  4. This neighborhood is a close-knit community |  | X |  |
| Freedom of movement | Index of women's freedom of movement for sanitation: If I need to use a sanitation location when at home during the day, I can go: If I need to use a sanitation location when away from home during the day, I can go: If I need to use a sanitation location at night, I can go to my household's daytime sanitation location: If I need to access water for sanitation-related needs, such as water for flushing, washing feces-soiled children's clothing, or managing my menstruation, I can go to the place where my household collects water: If I wanted to go to a sanitation-focused public gathering, community meeting, or training outside of my neighborhood, I could go: If I wanted to go to a sanitation-focused public gathering, community meeting, or training near my home, I could go: | X |  |  |
|  | India Human Development Survey - 2 Mobility measure, first subscale (Response options: no, must inform, yes) Please tell us whether you have to ask permission of your husband or a senior family member to go… to the local health center? to the home of relatives or friends [in the village / neighborhood]? a short distance by train or bus? |  | X | X |
| **Institutional structures** |  |  |  |  |
| Norms | Four items from the Gender Equity scale (Response options: Strongly disagree, Disagree, Agree, Strongly agree; Numbers 7 and 9 reverse scored for analysis) (Kostick, Schensul, Singh, Pelto, & Saggurti, 2011): A woman can talk to men other than her husband  A woman can participate in community activities if she wishes to  The status of women is lower than that of men A woman should finish all the household work before taking rest |  | X |  |
| Relations | Three items from the Brief Family Relationship Scale (Response options: Not at all; A little; Somewhat; Quite a bit; A lot; Number 9 reverse scored for analysis) (Fok, Allen, Henry, & Team, 2014): 7. In our family there is a feeling of togetherness 8. In our family we sometimes tell each other about our personal problems 9. In our family we lose our tempers a lot |  | X |  |

Cohen, M. (2000). Learning from Clients: Assessment Tools for Microfinance Practitioners Draft Manual.

Dahlem, N. W., Zimet, G. D., & Walker, R. R. (1991). The multidimensional scale of perceived social support: a confirmation study. *Journal of clinical psychology, 47*(6), 756-761.

Fok, C. C. T., Allen, J., Henry, D., & Team, P. A. (2014). The Brief Family Relationship Scale: A brief measure of the relationship dimension in family functioning. *Assessment, 21*(1), 67-72.

ICF. (2021). DHS Model Questionnaire - Phase 8. Retrieved from <http://www.dhsprogram.com>

Kostick, K. M., Schensul, S. L., Singh, R., Pelto, P., & Saggurti, N. (2011). A methodology for building culture and gender norms into intervention: An example from Mumbai, India. *Social science & medicine, 72*(10), 1630-1638.

Kroenke, K., Spitzer, R. L., Williams, J. B., & Löwe, B. (2009). An ultra-brief screening scale for anxiety and depression: the PHQ-4. *Psychosomatics, 50*(6), 613-621. doi:10.1176/appi.psy.50.6.613

Peterson, N. A., Lowe, J. B., Hughey, J., Reid, R. J., Zimmerman, M. A., & Speer, P. W. (2006). Measuring the intrapersonal component of psychological empowerment: Confirmatory factor analysis of the sociopolitical control scale. *American journal of community psychology, 38*(3), 287-297.

Pilkonis, P. A., Yu, L., Dodds, N. E., Johnston, K. L., Maihoefer, C. C., & Lawrence, S. M. (2014). Validation of the depression item bank from the Patient-Reported Outcomes Measurement Information System (PROMIS) in a three-month observational study. *J Psychiatr Res, 56*, 112-119. doi:10.1016/j.jpsychires.2014.05.010

Romppel, M., Herrmann-Lingen, C., Wachter, R., Edelmann, F., Düngen, H.-D., Pieske, B., & Grande, G. (2013). A short form of the General Self-Efficacy Scale (GSE-6): Development, psychometric properties and validity in an intercultural non-clinical sample and a sample of patients at risk for heart failure. *GMS Psycho-Social-Medicine, 10*.

Topp, C. W., Østergaard, S. D., Søndergaard, S., & Bech, P. (2015). The WHO-5 Well-Being Index: a systematic review of the literature. *Psychotherapy and Psychosomatics, 84*(3), 167-176. doi:10.1159/000376585

**Supplemental Table B: Latrine attributes for latrine used for daytime defecation by participants in Kampala, Uganda and Tiruchirappalli, India**

| **Characteristics** | **Kampala** | **Trichy** |
| --- | --- | --- |
| **Total Surveyed** | 1024 | 996 |
| **Location** |  |  |
| In own dwelling | 68 (6.6%) | 560 (56.2%) |
| In own yard/plot | 789 (77.1%) | 149 (15.0%) |
| Elsewhere | 165 (16.1%) | 285 (28.6%) |
| **Type of sanitation location** |  |  |
| Flush toilet | 175 (17.1%) | 277 (37.9%) |
| Pit latrine | 842 (82.%) | 400 (40.2%) |
| Buckets/hanging toilets/flying toilets | 0 (0%) | 155 (15.6%) |
| Open defecation | 2 (0.2%) | 36 (3.6%) |
| Composting toilets | 4 (0.4%) | 3 (0.3%) |
| Other | 0 (0%) | 21 (2.1%) |
| **Sharing** |  |  |
| Shares their facility | 860 (84.0%) | 264 (26.5%) |
| Shared with known households | 736 (71.9%) | 86 (8.6%) |
| Shared with general public | 131 (12.8%) | 253 (25.4%) |
| Average number of households using the shared sanitation location, mean (sd) | 11.2 (16.0) | 42.7 (38.5) |
| **Resources** |  |  |
| Money is paid to use sanitation location | 81 (7.9%) | 176 (17.7%) |
| Water is carried to use sanitation location | 632 (61.7%) | 187 (18.8%) |
| Would prefer to use another location but cannot afford to | 162 (15.8%) | 42 (4.2%) |
| **Time** |  |  |
| Time to walk to sanitation location, mean (sd) | 2.7 (3.5) | 5.6 (4.8) |
| Must wait to use sanitation location | 565 (55.2%) | 134 (13.5%) |
| **Privacy** |  |  |
| Sanitation location lockable from inside | 768 (75.0%) | 651 (65.4%) |
| Others can see you use this sanitation location | 173 (16.9%) | 77 (7.7%) |
| Sanitation location is located in a private place | 756 (73.8%) | 384 (38.6%) |
| **Lighting** |  |  |
| Sufficient lighting inside sanitation location | 579 (56.6%) | 694 (69.7%) |
| Sufficient lighting on the way to location | 727 (71.0%) | 846 (84.9%) |
| **Would use different location at night** | 153 (14.9%) | 94 (9.4%) |

**Supplemental Table C:** **Latrine attributes for latrine used for daytime urination by participants in Kampala, Uganda and Tiruchirappalli, India**

| **Characteristics** | **Kampala** | **Trichy** |
| --- | --- | --- |
| **Total Surveyed** | 1024 | 996 |
| **Location** |  |  |
| In own dwelling | 107 (10.4%) | 682 (68.5%) |
| In own yard/plot | 748 (73.0%) | 217 (21.8%) |
| Elsewhere | 166 (16.2%) | 96 (9.6%) |
| **Type of sanitation location** |  |  |
| Flush toilet | 167 (15.8%) | 297 (29.8%) |
| Pit latrine | 750 (73.2%) | 355 (35.6%) |
| Buckets/hanging toilets/flying toilets | 4 (0.4%) | 3 (0.3%) |
| Open defecation | 72 (7.0%) | 184 (18.5%) |
| Composting toilets | 3 (0.3%) | 92 (9.2%) |
| Other | 31 (3.0%) | 60 (6.0%) |
| **Sharing** |  |  |
| Shares their facility | 801 (78.2%) | 160 (16.1%) |
| Shared with known households | 694 (67.8%) | 107 (10.7%) |
| Shared with general public | 111 (10.8%) | 78 (7.8%) |
| Average number of households using the shared sanitation location, mean (sd) | 10.5 (14.4) | 24.7 (33.4) |
| **Resources** |  |  |
| Money is paid to use sanitation location | 66 (6.4%) | 55 (5.5%) |
| Water is carried to use sanitation location | 612 (59.8%) | 213 (21.4%) |
| **Time** |  |  |
| Time to walk to sanitation location, mean (sd) | 2.5 (3.2) | 3.8 (3.6) |
| Must wait to use sanitation location | 525 (51.3%) | 86 (8.6%) |
| **Privacy** |  |  |
| Sanitation location lockable from inside | 708 (69.1%) | 584 (58.6%) |
| Others can see you use this sanitation location | 166 (16.2%) | 80 (8.0%) |
| Sanitation location is located in a private place | 758 (74%) | 357 (35.8%) |
| **Lighting** |  |  |
| Sufficient lighting inside sanitation location | 545 (53.2%) | 613 (61.5%) |
| Sufficient lighting on the way to location | 713 (69.6%) | 837 (84.0%) |
| **Would use different location at night** | 576 (56.3%) | 133 (13.4%) |

**Supplemental Table D:** **Attributes for location used for menstruation by participants in Kampala, Uganda and Tiruchirappalli, India**

| **Characteristics** | **Kampala** | **Trichy** |
| --- | --- | --- |
| **Total Surveyed** | **1024** | **996** |
| **Materials most often used in past 3 cycles** |  |  |
| Reusable cloth | 97 (9.5%) | 61 (6.1%) |
| Disposable pad | 505 (49.3%) | 490 (49.2%) |
| Both pad and cloth | 32 (3.1%) | 32 (3.2%) |
| Nothing | 3 (0.3%) | 3 (0.3%) |
| **Where absorbent materials are changed at home during the day** |  |  |
| Private toilet in home | 17 (1.7%) | 254 (25.5%) |
| Bathroom in home | 47 (4.6%) | 175 (17.6%) |
| Other room in home | 379 (37.0%) | 36 (3.6%) |
| Private toilet outside of home | 120 (11.7%) | 68 (6.8%) |
| Shared or public toilet | 50 (4.9%) | 50 (5.0%) |
| **Where absorbent materials are changed at home at night** |  |  |
| Private toilet in home | 17 (1.7%) | 252 (25.3%) |
| Bathroom in home | 44 (4.3%) | 180 (18.1%) |
| Other room in home | 423 (41.3%) | 40 (4.0%) |
| Private toilet outside of home | 101 (9.9%) | 69 (6.9%) |
| Shared or public toilet | 31 (3.0%) | 40 (4.0%) |
| **Where absorbent materials are disposed** |  |  |
| Flushed down toilet/put in pit latrine | 235 (22.9%) | 13 (1.3%) |
| Rubbish bin inside/outside the home | 255 (24.9%) | 345 (34.6%) |
| Open drain | 6 (0.6%) | 22 (2.2%) |
| Elsewhere outside in the open | 12 (1.2%) | 61 (6.1%) |
| Burn it | 35 (3.4%) | 136 (13.7%) |
| Bury outside | 3 (0.3%) | 4 (0.4%) |
| Not disposed | 90 (8.8%) | 3 (0.3%) |

**Supplemental Table E: Descriptive statistics by sub-domain of Resources**

**Supplemental Table E.1: Response frequencies for all Health items among women in Kampala, Uganda and Tiruchirappalli, India, organized by factor analysis results and including items removed from scales (N=2,020)**

|  | **Full Sample (%)** | | | | |
| --- | --- | --- | --- | --- | --- |
|  | Never | Sometimes | Often | Always | Missing |
| ***Factor 1: Sanitation-related illness*** | | | | |  |
| Used a sanitation location that I believed might make me ill | 1284 (64) | 416 (21) | 161 (8) | 104 (5) | 55 (3) |
| Got sick as a result of using my sanitation location | 1461 (72) | 290 (14) | 152 (8) | 68 (3) | 49 (2) |
| Got sick as a result of cleaning my sanitation location | 1725 (85) | 147 (7) | 46 (2) | 8 (0) | 94 (5) |
| ***Factor 2: Illness due to suppression and withholding*** | | | | |  |
| Became ill because I had to suppress the urge to urinate or defecate | 1736 (86) | 155 (8) | 60 (3) | 13 (1) | 56 (3) |
| Withholding water to avoid urination made me feel unwell | 1715 (85) | 177 (9) | 61 (3) | 13 (1) | 54 (3) |
| Withholding food to avoid defecation made me feel unwell | 1800 (89) | 109 (5) | 46 (2) | 7 (0) | 58 (3) |
| ***Factor 3: Fear of injury*** | | | | |  |
| Feared being harassed or injured by men, boys, or other people when accessing my sanitation location | 1724 (85) | 128 (6) | 54 (3) | 21 (1) | 93 (5) |
| Feared being injured by animals or insects when accessing my sanitation location | 1653 (82) | 174 (9) | 78 (4) | 34 (2) | 81 (4) |
| Feared being injured because of the physical conditions - such as slippery conditions, rocks or thorns, uneven pathways, obstacles, sharp doors, or floors, etc. - when accessing my sanitation location | 1640 (81) | 186 (9) | 79 (4) | 35 (2) | 80 (4) |
| ***Factor 4: Sanitation-related anxiety, embarrassment, and shame*** | | | | |  |
| Experienced embarrassment or shame when accessing a sanitation location during the day | 1754 (87) | 136 (7) | 49 (2) | 27 (1) | 54 (3) |
| Experienced embarrassment or shame when accessing a sanitation location at night | 1775 (87) | 130 (6) | 36 (2) | 25 (1) | 54 (3) |
| Felt anxiety, stress, or tension when I needed to access a sanitation location during the day when at home | 1619 (80) | 187 (9) | 94 (5) | 63 (3) | 57 (3) |
| ***Factor 5: Sanitation-related stress and fear*** | | | | |  |
| Been too afraid to use a sanitation location because it is dark | 1406 (70) | 243 (12) | 159 (8) | 159 (8) | 53 (3) |
| Felt stress or frustration related to the sanitation conditions in my community | 1287 (64) | 275 (14) | 169 (8) | 225 (11) | 64 (3) |
| Felt stress or frustration related to the sanitation conditions in my household | 1540 (76) | 241 (12) | 90 (4) | 91 (5) | 58 (3) |
| Feared for the safety of women or children going to sanitation locations | 1348 (67) | 324 (16) | 132 (7) | 150 (7) | 66 (3) |
| ***Items removed from scales*** | | | | |  |
| Been injured when going to a sanitation location | 1768 (88) | 133 (7) | 51 (3) | 11 (1) | 57 (3) |
| Been bitten by an animal - such as a dog, snake, or insect - when going to a sanitation location | 1809 (90) | 85 (4) | 35 (2) | 14 (1) | 77 (4) |
| Felt anxiety, stress, or tension when I needed to access a sanitation location while away from home | 1338 (66) | 389 (19) | 143 (7) | 76 (4) | 74 (4) |
| Felt anxiety, stress, or tension when I needed to access a sanitation location at night when at home | 1469 (73) | 275 (14) | 127 (6) | 94 (5) | 55 (3) |
| Experienced stress or tension when I needed to access a location to change my menstrual materials/manage my menstruation | 947 (47) | 157 (8) | 53 (3) | 39 (2) | 824 (41) |
| Felt stress or tension when changing my menstrual materials/managing my menstruation | 962 (48) | 145 (7) | 55 (3) | 40 (2) | 818 (40) |
| Felt scared when changing my menstrual materials/managing my menstruation | 1017 (50) | 112 (6) | 46 (2) | 27 (1) | 818 (40) |

**Supplemental Table E.2: Response frequencies for all Bodily Integrity items among women in Kampala, Uganda and Tiruchirappalli, India, organized by factor analysis results and including items removed from scales (N=2,020)**

|  | **Full Sample (%)** | | | | |
| --- | --- | --- | --- | --- | --- |
|  | Never | Sometimes | Often | Always | Missing |
| ***Factor 1: Satisfaction with sanitation location*** | | | | |  |
| Felt satisfied with the sanitation location I used most often | 322 (16) | 284 (14) | 350 (17) | 1010 (50) | 54 (3) |
| Felt comfortable with the sanitation location I used most often | 319 (16) | 297 (15) | 355 (18) | 1000 (50) | 49 (2) |
| The sanitation location I used most often was clean enough | 229 (11) | 370 (18) | 423 (21) | 950 (47) | 48 (2) |
| ***Factor 2: Withholding and suppression*** | | | | |  |
| Withheld water to avoid the urge to urinate when at home during the day or at night | 1699 (84) | 164 (8) | 72 (4) | 40 (2) | 45 (2) |
| Withheld food to avoid the urge to defecate when at home either during the day or at night | 1794 (89) | 110 (5) | 49 (2) | 20 (1) | 47 (2) |
| Had to suppress the urge to urinate or defecate during the daytime when at home | 1689 (84) | 196 (10) | 76 (4) | 14 (1) | 45 (2) |
| Had to suppress the urge to urinate or defecate when I am away from home | 1442 (71) | 378 (19) | 105 (5) | 34 (2) | 61 (3) |
| Had to suppress the urge to urinate or defecate at night when at home | 1664 (82) | 212 (10) | 76 (4) | 20 (1) | 48 (2) |
| ***Items removed from scales*** | | | | |  |
| Found the sanitation location I used most often to be dirty/disgusting | 1174 (58) | 409 (20) | 183 (9) | 201 (10) | 53 (3) |
| Was able to access a satisfactory sanitation location when away from home | 398 (20) | 648 (32) | 329 (16) | 521 (26) | 124 (6) |
| Was typically able to access the resources I needed, like water, toilet paper, or soap, to clean myself after defecation (including practicing anal cleansing) | 141 (7) | 191 (9) | 433 (21) | 1202 (60) | 53 (3) |
| Withheld water to avoid the urge to urinate when I knew that I would be away from home | 1488 (74) | 308 (15) | 103 (5) | 65 (3) | 56 (3) |
| Withheld food to avoid the urge to defecate when I knew that I would be away from home | 1658 (82) | 188 (9) | 75 (4) | 40 (2) | 59 (3) |
| Was able to access a satisfactory location when going to change my menstrual materials/manage my menstruation when I am away from home | 266 (13) | 328 (16) | 165 (8) | 403 (20) | 858 (42) |
| Felt satisfied with the location I used most often to change my menstrual materials/manage my menstruation | 128 (6) | 136 (7) | 214 (11) | 720 (36) | 822 (41) |
| Was able to access a satisfactory location when going to change my menstrual materials/manage my menstruation when I am at home | 108 (5) | 148 (7) | 234 (12) | 706 (35) | 824 (41) |
| Was typically able to access the resources I needed, like water or soap, to clean myself when using a sanitation location during menstruation | 56 (3) | 135 (7) | 257 (13) | 750 (37) | 822 (41) |
| Had to delay changing my menstrual materials when I was away from home because I did not have access to a satisfactory sanitation location | 700 (35) | 291 (14) | 92 (5) | 95 (5) | 842 (42) |
| Had to delay changing my menstrual material during the daytime when at home because I did not have access to a satisfactory sanitation location | 899 (45) | 169 (8) | 53 (3) | 78 (4) | 821(41) |
| Had to delay changing my menstrual materials at night when at home because I did not have access to a satisfactory sanitation location | 941(47) | 160 (8) | 50 (2) | 47 (2) | 822 (41) |

**Supplemental Table E.3: Response frequencies for all Safety and Security items among women in Kampala, Uganda and Tiruchirappalli, India, organized by factor analysis results and including items removed from scales (N=2,020)**

|  | **Full Sample (%)** | | | | |
| --- | --- | --- | --- | --- | --- |
|  | Strongly disagree | Disagree | Agree | Strongly agree | Missing |
| ***Factor 1: Perceptions of women's risk of harm when going for sanitation*** | | | | |  |
| Women in my community face the risk of being physically harmed by men or boys when going to sanitation locations | 665 (33) | 858 (42) | 281 (14) | 74 (4) | 142 (7) |
| Women in my community face the risk of sexual assault when going to sanitation locations | 639 (32) | 946 (47) | 249 (12) | 55 (3) | 131 (6) |
| Women in my community face the risk of someone making sexual comments or saying obscene things to them when they go to sanitation locations | 676 (33) | 874 (43) | 279 (14) | 61 (3) | 130 (6) |
| ***Factor 2: Perceptions of women's risk of harm when going to sanitation-related meetings*** | | | | |  |
| Women in my community face the risk of someone making sexual comments or saying obscene things to them when they go to a sanitation-related meeting | 668 (33) | 953 (47) | 201 (10) | 58 (3) | 140 (7) |
| Women in my community face the risk of experiencing harassment, such as being called by rude names, yelling, or shaming, if they go to a sanitation-related meeting | 657 (33) | 956 (47) | 207 (10) | 67 (3) | 133 (7) |
| Women in my community face the risk of experiencing harassment, such as being called by rude names, yelling, or shaming, if they speak up in a sanitation-related meeting | 672 (33) | 950 (47) | 198 (10) | 61 (3) | 139 (7) |
| ***Factor 3: Perceptions of women's risk of domestic violence related to sanitation*** | | | | |  |
| Women in my community face the risk of being hit by their husbands or other family members if they go for sanitation without telling someone or stay out too long | 519 (26) | 848 (42) | 426 (21) | 98 (5) | 129 (7) |
| Women in my community face the risk of being hit by their husbands or other family members if they fail to complete sanitation-related chores | 461 (23) | 885 (44) | 429 (21) | 97 (5) | 148 (7) |
| Women in my community face the risk of being hit by their husbands or other family members if they argue with the head of their households / another family member about sanitation issues | 523 (26) | 916 (45) | 380 (19) | 63 (3) | 138 (7) |
| Women in my community face the risk of being hit by their husbands or other family members if they argue with neighbors or other people in the community about sanitation issues | 492 (24) | 954 (47) | 362 (18) | 75 (4) | 137 (7) |
|  | Never | Sometimes | Often | Always | Missing |
| ***Factor 4: Perceptions of own risk of harm when going for sanitation*** | | | | |  |
| Feared I would be physically harmed by someone when I went to a sanitation location when away from home | 1390 (69) | 341 (17) | 125 (6) | 70 (3) | 94 (5) |
| Feared I would be sexually assaulted when I went to a sanitation location when away from home | 1509 (75) | 272 (13) | 101 (5) | 44 (2) | 94 (5) |
| Feared someone would make sexual comments or say obscene things to me when I went to a sanitation location when away from home | 1540 (76) | 264 (13) | 88 (4) | 38 (2) | 90 (4) |
| Feared someone would expose himself or spy/peep on me when I went to a sanitation location when away from home | 1528 (76) | 260 (13) | 97 (5) | 43 (2) | 92 (5) |
| Felt unsafe when using a sanitation location outside the home that was not for women only | 1289 (64) | 331 (16) | 165 (8) | 150 (7) | 85 (4) |
| ***Factor 5: Perceptions of general personal safety related to sanitation*** | | | | |  |
| Felt unsafe in the place where I typically go for sanitation during the day | 1501 (74) | 186 (9) | 124 (6) | 134 (7) | 75 (4) |
| Felt unsafe in a place where I have gone for sanitation when away from home | 1132 (56) | 523 (26) | 150 (7) | 120 (6) | 95 (5) |
| Felt unsafe in the place where I typically go for sanitation at night | 1339 (66) | 299 (15) | 173 (9) | 144 (7) | 65 (3) |
| ***Items removed from scales*** | | | | |  |
|  | Strongly disagree | Disagree | Agree | Strongly agree | Missing |
| Women in my community face the risk of being physically harmed by men or boys if they go to a sanitation-related meeting | 675 (33) | 983 (49) | 155 (8) | 63 (3) | 144 (7) |
| Women in my community face the risk of being physically harmed by men or boys if they speak up in a sanitation-related meeting | 689 (34) | 986 (49) | 161 (8) | 47 (2) | 137 (7) |
| Women in my community face the risk of sexual assault when going to a sanitation-related meeting | 693 (34) | 1008 (50) | 146 (7) | 45 (2) | 128 (6) |
| Women in my community face the risk of someone exposing himself or spying/peeping on them when they go to sanitation locations | 625 (31) | 900 (45) | 266 (13) | 99 (5) | 130 (6) |
| Women in my community consider the public toilets to be unsafe | 298 (15) | 563 (28) | 543 (27) | 410 (20) | 206 (10) |
|  | Never | Sometimes | Often | Always | Missing |
| Chose to use a different sanitation location at night for safety reasons | 1521 (75) | 222 (11) | 116 (6) | 98 (5) | 63 (3) |
| Feared I would be physically harmed by someone when I went to a sanitation location at night | 1410 (70) | 313 (15) | 126 (6) | 90 (4) | 81 (4) |
| Feared I would be sexually assaulted when I went to a sanitation location at night | 1505 (75) | 264 (13) | 98 (5) | 63 (3) | 90 (4) |
| Feared someone would make sexual comments or say obscene things to me when I went to a sanitation location at night | 1646 (81) | 189 (9) | 64 (3) | 33 (2) | 88 (4) |
| Feared someone would expose himself or spy/peep on me when I went to a sanitation location at night | 1608 (80) | 219 (11) | 75 (4) | 33 (2) | 85 (4) |
| Feared someone would make sexual comments or say obscene things to me when I went to change my menstrual materials/manage my menstruation | 943 (47) | 94 (5) | 40 (2) | 115 (6) | 828 (41) |
| Felt safe in the place where I typically go to change my menstrual materials/manage my menstruation when I am at home | 217 (11) | 115 (6) | 187 (9) | 678 (34) | 823 (41) |
| Felt safe in the place where I typically go to change my menstrual materials/manage my menstruation when I am away from home | 271 (13) | 252 (12) | 151 (7) | 496 (25) | 850 (42) |
| The location I use to change my menstrual materials/manage my menstruation during the day is safe at night | 196 (10) | 124 (6) | 197 (10) | 675 (33) | 828 (41) |

**Supplemental Table E.4: Response frequencies for all Privacy items among women in Kampala, Uganda and Tiruchirappalli, India, organized by factor analysis results and including items removed from scales (N=2,020)**

|  | **Full Sample (%)** | | | | |
| --- | --- | --- | --- | --- | --- |
|  | Never | Sometimes | Often | Always | Missing |
| ***Factor 1: Privacy for sanitation*** | | | | |  |
| Worried that someone would see me urinating or defecating | 1607 (80) | 195 (10) | 97 (5) | 57 (3) | 64 (3) |
| Had to use a sanitation location that was not private enough for me when I was at home | 1583 (78) | 210 (10) | 86 (4) | 77 (4) | 64 (3) |
| Had to use a sanitation location that was not private enough for me when I was away from home | 1347 (67) | 405 (20) | 101 (5) | 76 (4) | 91 (5) |
| While at home, had to stop urinating or defecating because someone came near me and I no longer had privacy | 1504 (74) | 282 (14) | 94 (5) | 54 (3) | 86 (4) |
| While away from home, I had to stop urinating or defecating because someone came near me and I no longer had privacy | 1592 (79) | 237 (12) | 89 (4) | 45 (2) | 57 (3) |
| ***Items removed from scales*** | | | | |  |
| Felt that my sanitation location lacked privacy because men also use this location | 1485 (74) | 218 (11) | 133 (7) | 118 (6) | 66 (3) |
| Worried that someone would see me changing my menstrual materials/managing my menstruation while using a sanitation location | 988 (40) | 134 (7) | 32 (2) | 32 (2) | 834 (41) |
| Worried that someone would see me carrying menstrual materials on my way to a sanitation location | 964 (48) | 132 (7) | 54 (3) | 37 (2) | 833 (41) |
| Had to use locations to change my menstrual materials/manage my menstruation that were not private enough for me | 942 (47) | 170 (8) | 41 (2) | 40 (2) | 827 (41) |
| Had difficulty finding a private place to change my menstrual materials/manage my menstruation | 948 (47) | 173 (9) | 40 (2) | 36 (2) | 823 (41) |
| Worried that someone will see my menstrual blood in the sanitation location that I use to change my menstrual material | 927 (46) | 142 (7) | 74 (4) | 51 (3) | 826 (41) |
| Worried that someone would see me washing my menstrual materials in a sanitation location | 949 (47) | 132 (7) | 55 (3) | 32 (2) | 852 (42) |

**Supplemental Table E.5: Response frequencies for all Financial and Productive Assets items among women in Kampala, Uganda and Tiruchirappalli, India, organized by factor analysis results and including items removed from scales (N=2,020)**

|  | **Full Sample (%)** | | | | |
| --- | --- | --- | --- | --- | --- |
|  | Strongly disagree | Disagree | Agree | Strongly agree | Missing |
| ***Factor 1: Control over money for sanitation*** | | | | |  |
| Have control over money that I could use to contribute to a community sanitation project | 239 (12) | 736 (36) | 705 (35) | 258 (13) | 82 (4) |
| Have control over money that I could use to pay for household latrine/toilet construction | 208 (10) | 629 (31) | 799 (40) | 278 (14) | 106 (5) |
| I have control over money that I could use to pay for household latrine/toilet improvements or repairs |  |  |  |  |  |
| ***Factor 2: Ability to acquire money for sanitation*** | | | | |  |
| Could acquire money to build a household latrine/toilet by selling or renting something I own or by earning money through work | 234 (12) | 689 (34) | 737 (36) | 263 (13) | 97 (5) |
| Could acquire money to improve or repair a household latrine/toilet by selling or renting something I own or by earning money through work | 210 (10) | 657 (33) | 792 (39) | 256 (13) | 105 (5) |
| Could acquire money to build a household latrine/toilet by accessing credit or participating in a savings group | 247 (12) | 709 (35) | 725 (36) | 231 (11) | 108 (5) |
| ***Factor 4: Inadequate finances to meet basic sanitation needs*** | | | | | |
| Would need to ask permission before spending household money on small sanitation-related expenses, such as toilet paper, soap, or pay-per-use latrines | 289 (14) | 523 (26) | 870 (43) | 252 (4) | 86 (4) |
| Depend on someone else to pay for small sanitation-related expenses, such as toilet paper, soap, or pay-per-use latrines | 260 (13) | 607 (30) | 731 (36) | 339 (17) | 83 (4) |
| ***Items removed from scales*** | | | | |  |
| Have control over assets, like land, livestock, or jewelry, that I could sell to contribute to pay for household latrine/toilet construction | 300 (15) | 850 (42) | 576 (29) | 187 (9) | 107 (5) |
| Do not have an income or way of acquiring money to contribute to sanitation improvements | 210 (10) | 790 (39) | 638 (32) | 307 (15) | 307 (4) |
| Someone in my household might take money I am saving for sanitation projects or materials for their own use without first asking my permission | 473 (23) | 919 (45) | 389 (19) | 119 (6) | 120 (6) |
| Someone in my household might take my money to spend on construction, improvement, or repair of a latrine/toilet without my permission | 509 (25) | 885 (44) | 391 (19) | 110 (5) | 125 (6) |
| Often have to choose between paying for sanitation needs and other needs of my household | 118 (6) | 450 (22) | 1062 (53) | 306 (15) | 260 (41) |
| If I were to try to earn an income, I would not work in a place without access to a sanitation location | 120 (6) | 206 (10) | 805 (40) | 790 (39) | 99 (5) |
| Have control over money that I could use to meet my menstruation needs | 241 (12) | 670 (33) | 756 (37) | 255 (13) | 98 (4) |
| Have control over money that I could use to meet my menstruation needs | 142 (7) | 204 (10) | 580 (29) | 260 (13) | 824 (41) |
| Often have to choose between paying for menstruation-related needs and other needs of my household | 131 (6) | 313 (15) | 559 (28) | 187 (9) | 830 (41) |
| Would need to ask permission before spending household money on menstruation-related expenses | 275 (14) | 535 (26) | 268 (13) | 112 (6) | 830 (41) |
| Someone in my household might take money I am saving for menstruation needs for their own use | 251 (12) | 495 (25) | 322 (16) | 109 (5) | 843 (42) |
| Depend on someone else to pay for menstruation-related expenses | 197 (10) | 394 (20) | 406 (20) | 192 (10) | 831 (41) |
|  |  |  |  |  |  |

**Supplemental Table E.6: Response frequencies for all Time items among women in Kampala, Uganda and Tiruchirappalli, India, organized by factor analysis results and including items removed from scales (N=2,020)**

|  | **Full Sample (%)** | | | | |
| --- | --- | --- | --- | --- | --- |
|  | Strongly disagree | Disagree | Agree | Strongly agree | Missing |
| ***Factor 1: Time on sanitation-related responsibilities*** | | | | |  |
| The sanitation-related needs and responsibilities of my household prevent me from completing other household work | 608 (30) | 1042 (52) | 198 (10) | 65 (3) | 107 (5) |
| The sanitation-related needs and responsibilities of my household require that I often have to wake up earlier than I want | 581 (29) | 926 (45) | 327 (16) | 94 (5) | 102 (5) |
| The sanitation-related needs and responsibilities of my household often make me miss out on other activities that I would like to do | 635 (31) | 1045 (52) | 179 (9) | 57 (3) | 104 (4) |
| ***Factor 2: Time meeting personal sanitation needs*** | | | | |  |
| It often takes too much time to access and use my sanitation location | 638 (32) | 1011 (50) | 227 (11) | 65 (3) | 79 (4) |
| Often have to wake up earlier than I want to access a sanitation location | 633 (31) | 957 (47) | 261 (13) | 88 (4) | 81 (4) |
| Often have to rush when I am using my sanitation location | 612 (30) | 919 (45) | 296 (15) | 109 (5) | 84 (4) |
| ***Items removed from scales*** | | | | |  |
| Spend more time than I want to each day on sanitation-related chores, such as cleaning the latrine/toilet or assisting others in my household with their sanitation-related needs | 505 (25) | 889 (44) | 391 (19) | 121 (6) | 114 (6) |
| The sanitation-related needs and responsibilities of my household make me miss out on income-generating activities that I would like to do | 603 (30) | 1062 (53) | 187 (9) | 60 (3) | 108 (5) |
| Would like to have more time to meet my menstruation-related needs | 238 (12) | 486 (24) | 310 (15) | 152 (8) | 834 )41) |
| Often have to wake up earlier than I want to access a location to manage my menstruation | 281 (14) | 522 (26) | 271 (13) | 113 (6) | 832 (41) |
| Often miss out on activities I would like to do because of time spent taking care of my menstruation-related needs | 356 (18) | 604 (30) | 158 (8) | 72 (4) | 830 (41) |

**Supplemental Table E.7: Response frequencies for all Social Capital items among women in Kampala, Uganda and Tiruchirappalli, India, organized by factor analysis results and including items removed from scales (N=2,020)**

|  | **Full Sample (%)** | | | | |
| --- | --- | --- | --- | --- | --- |
|  | Strongly disagree | Disagree | Agree | Strongly agree | Missing |
| ***Factor 1: Social capital to improve sanitation conditions and practices*** | | | | |  |
| Have family members who I can talk to about problems related to my sanitation location | 89 (4) | 235 (12) | 1233 (61) | 362 (18) | 101 (5) |
| Have someone in my household who would help with chores, like cooking or providing childcare, so I could tend to my sanitation needs | 154 (8) | 495 (25) | 953 (47) | 321 (16) | 97 (5) |
| Have family members who would help me with sanitation-related chores | 115 (6) | 379 (19) | 1007 (50) | 417 (21) | 102 (5) |
| Have someone in my household who could get small sanitation-related items if I asked them to, such as if I were too busy | 108 (5) | 235 (12) | 1185 (59) | 395 (20) | 97 (5) |
| ***Factor 2: Social capital within the community*** | | | | |  |
| Know leaders in the community who I can talk to about problems related to my sanitation location | 208 (10) | 522 (26) | 848 (42) | 299 (15) | 143 (7) |
| Know other members of my community who I would feel comfortable asking to help me address a sanitation-related problem in the community | 183 (9) | 558 (28) | 912 (45) | 256 (13) | 111 (5) |
| Have connections to someone with the power to improve sanitation conditions for my household or community | 249 (12) | 762 (38) | 647 (32) | 242 (12) | 120 (6) |
| Know someone who can give me information about better sanitation practices | 141 (7) | 586 (29) | 842 (42) | 320 (16) | 131 (6) |
| ***Items removed from scales*** | | | | |  |
| Have friends or neighbors who I can talk to about problems related to my sanitation location | 114 (6) | 400 (20) | 1067 (53) | 344 (17) | 95 (5) |
| Have a friend or neighbor who would help with chores, like cooking or providing childcare, so I could tend to my sanitation needs | 219 (11) | 673 (33) | 791 (39) | 240 (12) | 97 (5) |
| Have friends or neighbors who would help me with sanitation-related chores | 167 (8) | 619 (31) | 869 (43) | 268 (13) | 97 (%) |
| Have a friend or neighbor who could get small sanitation-related items if I asked them to, such as if I were too busy | 159 (8) | 534 (26) | 944 (47) | 290 (14) | 93 (5) |
| Have friends who could help me complete a sanitation-related project, such as repairing or improving my latrine/toilet | 182 (9) | 675 (33) | 768 (38) | 266 (13) | 129 (6) |
| Have friends or neighbors who would encourage or help me organize an initiative to improve sanitation conditions in the community | 174 (9) | 586 (28) | 839 (42) | 308 (15) | 113 (6) |
| Have friends or neighbors who make me aware of and/or encourage me to attend community meetings/events related to sanitation | 164 (8) | 521 (26) | 945 (47) | 280 (14) | 110 (5) |
| Have a friend or family member who would help me with household chores, such as cooking or childcare, so that I could attend a sanitation-related meeting | 174 (9) | 633 (31) | 844 (42) | 259 (13) | 110 (5) |
| Have a friend or family member who will accompany me to a sanitation-related meeting if I ask them to | 138 (7) | 505 (25) | 906 (45) | 364 (18) | 107 (5) |
| Have someone in my household who could get menstruation-related items if I asked them to, such as if I were too busy | 121 (6) | 256 (13) | 580 (29) | 221 (11) | 842 (42) |
| Have a friend or neighbor who could get menstruation-related items if I asked them to, such as if I were too busy | 176 (9) | 391 (19) | 441 (22) | 168 (8) | 844 (42) |

**Supplemental Table E.8: Response frequencies for all Knowledge and Skills items among women in Kampala, Uganda and Tiruchirappalli, India, organized by factor analysis results and including items removed from scales (N=2,020)**

|  | **Full Sample (%)** | | | | |
| --- | --- | --- | --- | --- | --- |
|  | Strongly disagree | Disagree | Agree | Strongly agree | Missing |
| ***Factor 1: Knowledge and skills related to community sanitation*** | | | | |  |
| Have skills - such as budgeting, organization, or mobilizing people in my community - to contribute to sanitation-related projects in my community | 281 (14) | 863 (43) | 599 (30) | 164 (8) | 113 (6) |
| Know how decisions about sanitation are made in my community | 229 (11) | 824 (41) | 594 (29) | 191 (9) | 182 (9) |
| Know how to manage money for a sanitation project in my community | 281 (14) | 974 (48) | 469 (23) | 133 (7) | 163 (8) |
| ***Factor 2: Knowledge related to personal sanitation repairs and maintenance*** | | | | |  |
| If my toilet/latrine needed repairs, I would know how to get it fixed | 99 (5) | 544 (27) | 908 (45) | 324 (16) | 145 (7) |
| Know how to make minor repairs or improvements to a latrine/toilet, like unclogging, replacing a lightbulb, or fixing a door | 175 (9) | 599 (30) | 852 (42) | 255 (13) | 139 (7) |
| ***Factor 3: Knowledge to influence community-level sanitation decisions*** | | | | |  |
| Have enough knowledge to contribute to decisions on sanitation in my community | 103 (5) | 462 (23) | 980 (49) | 359 (18) | 116 (6) |
| Am knowledgeable about my community's sanitation issues | 107 (5) | 416 (21) | 987 (49) | 382 (19) | 128 (6) |
| If my community had the opportunity to improve our sanitation conditions, I would have ideas about what the community needs most | 121 (6) | 431 (21) | 1054 (52) | 300 (15) | 114 (6) |
| ***Factor 4: Knowledge and skills to influence household-level sanitation decisions*** | | | | |  |
| Have skills - such as budgeting and organization - to contribute to sanitation-related projects in my household | 87 (4) | 377 (19) | 1132 (56) | 321 (16) | 103 (5) |
| Know how to manage money for a sanitation project in my household | 85 (4) | 357 (18) | 1048 (52) | 411 (20) | 119 (6) |
| ***Items removed from scales*** | | | | |  |
| Know who to contact to report or resolve a sanitation-related problem in my community | 156 (8) | 537 (37) | 862 (43) | 328 (16) | 137 (7) |
| Know of an individual or company that provides sanitation-related services in this community, such as latrine emptying or toilet repair | 203 (10) | 693 (34) | 733 (36) | 253 (13) | 138 (7) |
| Know of a current project or initiative to improve sanitation conditions in my community | 315 (16) | 819 (41) | 567 (28) | 137 (7) | 182 (9) |
| Know of an organization or group that works to improve sanitation conditions in my community | 283 (14) | 764 (38) | 609 (30) | 197 (10) | 167 (8) |
| Feel that my knowledge helps to influence sanitation-related decisions in my community | 160 (8) | 614 (30) | 894 (44) | 227 (11) | 125 (6) |
| Am knowledgeable about health issues related to sanitation | 38 (2) | 210 (10) | 1231 (61) | 441 (22) | 100 (5) |
| Feel that my knowledge helps to influence sanitation-related decisions in my household | 32 (2) | 217 (11) | 1285 (64) | 393 (19) | 93 (5) |
| If our family had the opportunity to improve our sanitation conditions, I would know what our family needs most | 52 (3) | 197 (10) | 1232 (61) | 433 (21) | 106 (5) |
| Know what should and should not be put in the latrine/toilet to make sure it still works | 43 (2) | 216 (11) | 1090 (54) | 563 (28) | 108 (5) |
| Know about at least three types of toilets or sanitation options | 90 (4) | 656 (32) | 758 (38) | 372 (18) | 144 (7) |

**Supplemental Table E.9: Response frequencies for all Critical Consciousness items among women in Kampala, Uganda and Tiruchirappalli, India, organized by factor analysis results and including items removed from scales (n=2020)**

|  | **Full Sample (%)** | | | | |
| --- | --- | --- | --- | --- | --- |
|  | Strongly disagree | Disagree | Agree | Strongly agree | Missing |
| ***Scale 1: Self-Efficacy*** | | | | | |
| ***Factor 1: Ability to create change in the community*** | | | | |  |
| Feel I can change sanitation conditions in my community if I want to | 353 (17) | 764 (38) | 633 (31) | 174 (9) | 96 (5) |
| Feel like I can influence sanitation behaviors of others in my community, such as encouraging proper latrine use and maintenance, proper disposal of sanitary napkins, etc. | 264 (13) | 530 (26) | 886 (44) | 237 (12) | 103 (5) |
| ***Factor 2: Ability to create change at home*** | | | | |  |
| Feel I can change sanitation conditions in my household or compound if I want to | 65 (3) | 242 (12) | 1097 (54) | 540 (27) | 76 (4) |
| Feel like I can influence sanitation behaviors of members of my household | 47 (2) | 155 (8) | 1204 (60) | 541 (27) | 73 (4) |
| If I had a problem related to sanitation, I could probably think of a solution | 28 (1) | 138 (7) | 1290 (64) | 491 (24) | 71 (4) |
| If I had a problem related to sanitation, I believe I could solve it | 21 (1) | 163 (8) | 1264 (63) | 501 (25) | 71 (4) |
| ***Scale 2: Identifying and Questioning Inequalities*** | | | | | |
| ***Factor 1: Inequalities in sanitation access*** | | | | |  |
| Women have problems that men do not have when accessing or using a sanitation location | 31 (2) | 77 (4) | 1104 (55) | 739 (37) | 69 (3) |
| It is unfair when women have problems accessing or using a sanitation location but men do not have those problems | 133 (7) | 392 (19) | 917 (45) | 497 (25) | 81 (4) |
| Women typically have to delay going to a sanitation location more often than men because of their responsibilities | 135 (7) | 484 (24) | 828 (41) | 495 (25) | 78 (4) |
| ***Factor 2: Inequalities in sanitation-related decision-making*** | | | | |  |
| Women are less involved than men in making decisions about sanitation in the community | 193 (10) | 531 (26) | 853 (42) | 336 (17) | 107 (5) |
| It is fair for men to have the final say when making sanitation-related decisions in the household | 268 (13) | 668 (33) | 762 (38) | 237 (12) | 85 (4) |
| It is fair for men to have the final say when making sanitation-related decisions for the community | 268 (13) | 679 (34) | 744 (37) | 235 (12) | 94 (5) |
| ***Items removed from scales*** | | | | |  |
| The sanitation location I typically use makes me feel undignified | 424 (21) | 854 (42) | 422 (21) | 251 (12) | 69 (3) |
| The sanitation conditions in my community makes me feel undignified and ashamed | 217 (11) | 623 (31) | 636 (31) | 432 (21) | 112 (6) |
| The location I typically use for changing my menstrual materials/managing my menstruation makes me feel undignified | 297 (15) | 535 (26) | 798 (40) | 269 (13) | 121 (6) |
| If a man needs to urinate when away from home, he can find a place to do so more easily than a woman | 31 (2) | 56 (3) | 1071 (53) | 798 (40) | 64 (3) |
| If a man needs to defecate when away from home, he can find a place to do so more easily than a woman | 192 (10) | 426 (21) | 810 (40) | 481 (24) | 111 (5) |
| Men typically have to delay going to a sanitation location more often than women because of their responsibilities | 320 (16) | 926 (46) | 465 (23) | 197 (10) | 112 (6) |
| Women typically need to wait a longer time than men to use a sanitation location because there are not enough facilities to meet their needs | 64 (3) | 453 (22) | 982 (49) | 430 (21) | 91 (5) |
| Women have more sanitation-related responsibilities than men, such as cleaning the latrine/toilet | 30 (1) | 122 (6) | 947 (47) | 854 (42) | 67 (3) |
| In this community, those in power - such as landlords or city authorities - typically address sanitation-related problems adequately and in a timely manner | 297 (15) | 535 (26) | 798 (40) | 269 (13) | 121 (6) |

**Supplemental Table F: Descriptive statistics by sub-domain of Agency**

**Supplemental Table F.1: Response frequencies for all Decision-Making items among women in Kampala, Uganda and Tiruchirappalli, India, organized by factor analysis results and including items removed from scales (N=2,020)**

|  | **Full Sample (%)** | | | | |
| --- | --- | --- | --- | --- | --- |
|  | Strongly disagree | Disagree | Agree | Strongly agree | Missing |
| ***Factor 1: Ability to participate in household-level decision-making*** | | | | |  |
| If my household was making a decision about sanitation-related issues, I could be present for the discussion | 20 (1) | 101 (5) | 1254 (62) | 537 (27) | 108 (5) |
| If my household was making a decision about sanitation-related issues, I would be welcome to participate in the discussion | 23 (1) | 99 (5) | 1227 (57) | 559 (28) | 112 (6) |
| I would feel comfortable expressing my opinion about sanitation issues in household discussions | 12 (1) | 89 (4) | 1159 (57) | 654 (32) | 106 (5) |
| ***Factor 2: Ability to make small household-level decisions*** | | | | |  |
| If my household had decisions to make about small sanitation-related purchases, like soap, toilet paper, etc., I could independently make those decisions | 56 (3) | 248 (12) | 1077 (53) | 519 (26) | 120 (6) |
| I can independently make decisions about how my household will clean and maintain the sanitation environment/facility | 52 (3) | 200 (10) | 1062 (53) | 590 (29) | 116 (6) |
| ***Factor 3: Ability to make large household-level decisions*** | | | | |  |
| If my household had a major decision to make about sanitation, such as construction or large repairs, I could independently make that decision | 155 (8) | 609 (30) | 773 (38) | 337 (17) | 146 (7) |
| If my household had decisions to make about latrine/toilet repairs or enhancements, like new floor tiles, doors, locks, or lights, I could independently make that decision | 165 (8) | 609 (30) | 798 (40) | 306 (15) | 142 (7) |
| ***Factor 4: Ability to speak up in community-level decision-making*** | | | | |  |
| I would feel comfortable expressing my opinion about sanitation issues at a community meeting when men are present | 115 (6) | 375 (19) | 1016 (50) | 400 (20) | 114 (6) |
| If I spoke up in a community meeting about sanitation issues, it is likely that some others would listen | 59 (3) | 336 (17) | 1059 (52) | 366 (18) | 200 (10) |
| If I shared my opinion about sanitation issues with local leaders, NGOs, or government officials, it is likely that they would listen | 103 (5) | 414 (20) | 1000 (50) | 272 (13) | 231 |
| ***Factor 5: Ability to influence community-level decision-making*** | | | | |  |
| If my community had a major decision to make about sanitation, such as constructing public toilets, I could influence that decision | 145 (7) | 645 (32) | 810 (40) | 233 (12) | 187 (9) |
| If my community had decisions to make about latrine/toilet repairs or enhancements, like new floor tiles, doors, locks, or lights, I could influence those decisions | 163 (8) | 698 (35) | 778 (39) | 194 (10) | 187 (9) |
| If my community had decisions to make about maintenance or cleaning of latrines/toilets, I could influence those decisions | 151 (7) | 608 (30) | 813 (40) | 268 (13) | 180 (9) |
| ***Items removed from scales*** |  |  |  |  |  |
| If I wanted to go to a community meeting about sanitation, I would be welcomed | 70 (3) | 295 (15) | 1015 (50) | 484 (24) | 156 (8) |
| If there was a community meeting about sanitation issues in my neighborhood, I could attend | 81 (4) | 275 (14) | 1069 (53) | 485 (24) | 110 (5) |
| I would feel comfortable expressing my opinion about sanitation issues at a community meeting when only women are present | 50 (3) | 134 (7) | 982 (49) | 739 (37) | 115 (6) |
| If I spoke up in a household discussion about sanitation issues, it is likely that members of my household would listen | 18 (1) | 102 (5) | 1238 (61) | 554 (27) | 108 (5) |
| If my household had a major decision to make about sanitation, such as construction or large repairs, I could influence that decision | 52 (3) | 401 (20) | 1014 (50) | 403 (20) | 150 (7) |
| If my household had decisions to make about latrine/toilet repairs or enhancements, like new floor tiles, doors, locks, or lights, I could influence that decision | 71 (4) | 406 (20) | 1018 (50) | 370 (18) | 155 (8) |
| If my household had decisions to make about small sanitation-related purchases, like soap, toilet paper, etc., I could influence that decision | 32 (2) | 195 (10) | 1106 (55) | 565 (28) | 122 (6) |
| I can influence how my household will clean and maintain the sanitation environment/facility | 24 (1) | 163 (8) | 1080 (53) | 634 (31) | 119 (6) |
| I would feel comfortable expressing my opinion about menstruation-related sanitation issues at a community meeting when men are present | 428 (21) | 728 (36) | 515 (25) | 233 (12) | 116 (6) |
| I would feel comfortable expressing my opinion about menstruation-related sanitation issues at a community meeting when only women are present | 72 (4) | 95 (7) | 964 (5) | 775 (38) | 114 (6) |
| If my community had decisions to make about making toilets more female-friendly, such as installing rubbish bins for menstrual materials, I could influence those decisions | 139 (7) | 465 (23) | 942 (46) | 299 (15) | 175 (9) |
| I would feel comfortable expressing my opinion about menstruation-related sanitation issues in household discussions | 75 (4) | 303 (15) | 1044 (52) | 450 (22) | 148 (7) |
| If my household had decisions to make about making the toilet more female-friendly, such as buying a rubbish bin for menstrual materials, I could influence that decision | 86 (4) | 290 (14) | 1118 (55) | 366 (18) | 160 (8) |
| If my household had decisions to make about making the toilet more female-friendly, such as buying a rubbish bin for menstrual materials, I could have the final say on that decision | 164 (8) | 401 (20) | 972 (48) | 321 (16) | 162 (8) |

**Supplemental Table F.2: Response frequencies for all Leadership items among women in Kampala, Uganda and Tiruchirappalli, India, organized by factor analysis results and including items removed from scales (N=2,020)**

|  | **Full Sample (%)** | | | | |
| --- | --- | --- | --- | --- | --- |
|  | Strongly disagree | Disagree | Agree | Strongly agree | Missing |
| ***Factor 1: Support for women’s leadership*** | | | | |  |
| Believe that only men should be leaders in sanitation initiatives in my community | 279 (14) | 728 (36) | 278 (14) | 82 (4) | 653 (32) |
| Men in my community should support women's leadership in sanitation organizations | 13 (1) | 53 (3) | 823 (41) | 489 (24) | 642 (32) |
| Women in my community should support women's leadership in sanitation organizations | 5 (0) | 23 (1) | 784 (39) | 567 (28) | 641 (32) |
| ***Factor 2: Belief in women’s ability to lead*** |  |  |  |  |  |
| Generally trust sanitation groups/organizations with men as the head of the organization/group more than those with women as the head | 135 (7) | 489 (24) | 490 (24) | 257 (13) | 649 (32) |
| Trust sanitation groups/organizations with women as the treasurers/money-keepers more than those with men as the treasurers/money-keepers | 36 (2) | 146 (7) | 744 (37) | 444 (22) | 650 (32) |
| Women are as capable as men to take on official leadership roles in sanitation initiatives | 7 (0) | 30 (1) | 776 (38) | 444 (28) | 642 (32) |
| Women are as capable as men to take on informal leadership roles for sanitation-related issues in this community, such as sanitation education, latrine promotion, or solving disputes | 4 (0) | 22 (1) | 747 (37) | 604 (30) | 643 (32) |
| ***Items removed from scales*** |  |  |  |  |  |
| Local leaders should support women's leadership in sanitation organizations | 10 (0) | 24 (1) | 805 (40) | 533 (26) | 648 (32) |
| Would listen to a knowledgeable woman's advice about sanitation-related issues even if she were not in an official leadership role | 4 (0) | 12 (1) | 821 (41) | 540 (27) | 643 (32) |

**Supplemental Table F.3: Response frequencies for all Collective Action items among women in Kampala, Uganda and Tiruchirappalli, India, organized by factor analysis results and including items removed from scales (N=2,020)**

|  | **Full Sample (%)** | | | | |
| --- | --- | --- | --- | --- | --- |
|  | Strongly disagree | Disagree | Agree | Strongly agree | Missing |
| ***Factor 1: Shared goals and interests*** | | | | |  |
| Community members and I share common goals for improving sanitation in this community | 211 (11) | 536 (27) | 868 (43) | 243 (12) | 158 (8) |
| The sanitation-related goals I share with my community motivate me to work with others, even when that is challenging | 216 (11) | 528 (26) | 901 (45) | 218 (11) | 157 (8) |
| ***Factor 2: Sense of trust and community*** | | | | |  |
| I am confident that members of my community will work with one another to achieve sanitation-related goals | 88 (4) | 383 (19) | 1065 (53) | 350 (17) | 124 (7) |
| People in this community would be willing to contribute time/labor, money, or other resources toward common sanitation goals | 93 (5) | 469 (23) | 992 (49) | 295 (15) | 171 (8) |
| If I need help with a sanitation-related project, there are people in this community who I could trust to help me | 105 (5) | 513 (25) | 972 (48) | 257 (13) | 173 (9) |
| ***Factor 3: Sense of mutual support*** | | | | |  |
| If there was a sanitation problem in this community, it is likely that people would cooperate to try to solve the problem | 63 (3) | 335 (17) | 1170 (58) | 315 (16) | 127 (7) |
| If there is a problem that affects the entire community, people in this community would help each other to address the issue | 65 (3) | 300 (15) | 1157 (57) | 363 (18) | 135 (7) |
| Most people in this community would be willing to help each other with sanitation-related projects | 71 (4) | 351 (17) | 1069 (53) | 385 (19) | 144 (7) |
| ***Items removed from scales*** |  |  |  |  |  |
| If a sanitation project does not directly benefit me, but has benefits for many others in the community, I would be likely to contribute time/labor, money, or other resources to the project | 119 (6) | 362 (18) | 1019 (50) | 408 (20) | 112 (6) |
| This community has good leaders for sanitation-related initiatives and projects | 170 (8) | 490 (24) | 827 (49) | 324 (16) | 209 (10) |
| If I have a sanitation-related problem, I could trust that members of my community would not shame me | 92 (5) | 403 (20) | 1055 (52) | 297 (15) | 173 (9) |

**Supplemental Table F.4: Response frequencies for all Freedom of Movement items among women in Kampala, Uganda and Tiruchirappalli, India, organized by factor analysis results and including items removed from scales (N=2,020)**

|  | **Full Sample (%)** | | | | | |
| --- | --- | --- | --- | --- | --- | --- |
|  | Alone without telling anyone | Alone if I tell someone | Alone with permission | Only with accompaniment | Not at all | Missing |
| ***Factor 1: Freedom of movement for meeting personal sanitation needs*** | | | | |  |  |
| If I need to use a sanitation location when at home during the day, I can go | 1801 (89) | 82 (4) | 24 (1) | 3 (0) | 4 (0) | 106 (5) |
| If I need to use a sanitation location when away from home during the day, I can go | 1351 (67) | 279 (14) | 222 (11) | 15 (1) | 35 (2) | 118 (6) |
| If I need to use a sanitation location at night, I can go to my household's daytime sanitation location | 1543 (76) | 207 (10) | 51 (3) | 89 (4) | 18 (1) | 112 (6) |
| ***Factor 2: Freedom of movement for attending sanitation-focused events*** | | | | |  |  |
| If I wanted to go to a sanitation-focused public gathering, community meeting, or training outside of my neighborhood, I could go | 887 (44) | 547 (27) | 355 (18) | 49 (2) | 68 (3) | 114 (6) |
| If I wanted to go to a sanitation-focused public gathering, community meeting, or training near my home, I could go | 870 (43) | 561 (28) | 365 (18) | 52 (3) | 61 (3) | 111 (5) |
| ***Items removed from scales*** |  |  |  |  |  |  |
| If I need to access water for sanitation-related needs, such as water for flushing, washing feces-soiled children's clothing, or managing my menstruation, I can go to the place where my household collects water | 1597 (79) | 102 (5) | 71 (4) | 28 (1) | 90 (4) | 132 (7) |
| If I need to access water for menstruation-related needs, such as washing myself or washing my menstrual materials, I can go to the place where my household collects water | 1005 (50) | 46 (2) | 40 (2) | 16 (1) | 51 (3) | 862 (43) |
| If I need to change my menstrual materials when at home during the day, I can go | 1131 (56) | 29 (1) | 7 (0) | 2 (0) | 4 (0) | 847 (42) |
| If I need to change my menstrual materials when away from home during the day, I can go | 906 (45) | 118 *6) | 105 (5) | 0 (0) | 35 (2) | 856 (42) |
| If I need to change my menstrual materials at night, I can go to my household's daytime sanitation location | 1045 (52) | 63 (3) | 26 (1) | 23 (1) | 6 (0) | 857 (42) |

**Supplemental Table G: Descriptive statistics by sub-domain of Institutional Structure**

**Supplemental Table G.1: Response frequencies for all Norms items among women in Kampala, Uganda and Tiruchirappalli, India, organized by factor analysis results and including items removed from scales (N=2,020)**

|  | **Full Sample (%)** | | | | |
| --- | --- | --- | --- | --- | --- |
|  |  |  |  |  |  |
|  | Strongly disagree | Disagree | Agree | Strongly agree | Missing |
| ***Factor 1: Gendered household roles and responsibilities*** | | | | |  |
| In this community, it is women more often than men who are expected to assume most responsibilities related to maintaining the cleanliness of the sanitation location the family uses | 31 (2) | 70 (3) | 921 (46) | 914 (45) | 84 (4) |
| In this community, it is women more often than men who are expected to accompany or support elderly, sick, or disabled family members who cannot urinate or defecate on their own | 32 (2) | 71 (3) | 1018 (50) | 799 (40) | 100 (5) |
| In this community, it is women more often than men who are expected to accompany or support children in their household when they need to urinate or defecate | 16 (1) | 44 (2) | 938 (46) | 939 (46) | 83 (4) |
| In this community, it is women more often than men who are expected to clean feces in their home or household compound from children or other family members | 16 (1) | 31 (2) | 959 (47) | 932(46) | 82 (4) |
| In this community, it is women more often than men who are expected to wash clothes that become dirty as a result of sanitation conditions or accidents | 16 (1) | 35 (2) | 971 (48) | 915 (45) | 83 (4) |
| In this community, it is women more often than men who are expected to clean their children after defecation if needed | 15 (1) | 31 (2) | 914 (45) | 974 (48) | 86 (4) |
| ***Factor 2: Acceptability of women disagreeing with their husbands*** | | | | |  |
| In this community, it is acceptable for a woman to express a different opinion than her husband in a household discussion about sanitation issues. | 62 (3) | 223 (11) | 1169 (58) | 438 (22) | 128 (6) |
| In this community, it is acceptable for a woman to express a different opinion than her husband about sanitation issues in front of people outside of the family | 170 (8) | 440 (22) | 929 (46) | 344 (17) | 137 (7) |
| ***Factor 3: Acceptability of women’s participation in community-level sanitation-related activities*** | | | | |  |
| In this community, it is socially acceptable for women to have leadership roles in sanitation-focused committees or organizations | 20 (1) | 90 (4) | 1149 (57) | 636 (31) | 125 (6) |
| In this community, it is acceptable for women to bring a complaint about a sanitation-problem to a local leader | 15 (1) | 108 (5) | 1191 (59) | 582 (29) | 124 (6) |
| It would be socially acceptable for women to organize an initiative to improve sanitation conditions in the community | 48 (2) | 118 (6) | 1245 (62) | 478 (24) | 131 (6) |
| If there was a community initiative to improve sanitation, it would be socially acceptable for women to participate | 14 (1) | 61 (3) | 1227 (61) | 598 (30) | 120 (6) |
| ***Factor 4: Acceptability of women’s participation in sanitation-related meetings*** | | | | |  |
| It is appropriate for women to attend sanitation-related meetings where men are present | 47 (2) | 136 (7) | 1163 (58) | 572 (28) | 102 (5) |
| In this community, it is considered appropriate for a woman to express her opinion about sanitation issues at a community meeting when men are present | 55 (3) | 174 (9) | 1158 (57) | 530 (26) | 103 (5) |
| It is appropriate for women to discuss sanitation-related issues in front of men | 83 (4) | 215 (11) | 1089 (54) | 531 (26) | 102 (5) |
| ***Factor 5: Restrictions on women speaking at sanitation-related meetings*** | | | | | |
| At a sanitation-related meeting where both men and women are present, women should only speak when they are asked to do so | 448 (22) | 925 (46) | 398 (20) | 146 (7) | 103 (5) |
| At a sanitation-related meeting where both men and women are present, women should only speak after all the men have shared their opinions | 481 (24) | 976 (48) | 305 (15) | 155 (8) | 103 (5) |
| At a sanitation-related meeting where both men and women are present, women should not speak | 726 (36) | 966 (48) | 144 (7) | 88 (4) | 96 (5) |
| ***Factor 6: Gendered expectations surrounding sanitation value chain work*** | | | | | |
| Even if women were trained, it would be socially unacceptable for women to do construction, repairs, or upgrades for latrines | 141 (7) | 431 (21) | 821 (41) | 517 (26) | 110 (5) |
| Technical work, like latrine construction, repairs, or upgrades, should be done by men not women | 71 (4) | 293 (15) | 983 (49) | 584 (29) | 89 (4) |
| Emptying latrine pits should be done by men not women | 25 (1) | 121 (6) | 834 (41) | 945 (47) | 95 (5) |
| ***Items removed from scales*** |  |  |  |  |  |
| It is socially acceptable for women to be involved in sanitation-focused businesses | 46 (2) | 124 (6) | 1123 (56) | 632 (31) | 95 (5) |
| In this community, it is acceptable for a wife to decide to go to a sanitation-related meeting without first consulting her husband | 317 (16) | 809 (40) | 548 (27) | 222 (11) | 124 (6) |
| In this community, it is acceptable for women to attend sanitation-related meetings | 33 (2) | 128 (6) | 1196 (59) | 552 (27) | 111 (5) |
| In this community, men are expected to make the final decision on major sanitation-related decisions for the household, such as construction, repairs, or upgrades | 187 (9) | 482 (24) | 895 (44) | 331 (16) | 125 (6) |
| In this community, it is acceptable for husbands to make important decisions about the household's sanitation facility without asking their wives' opinions | 301 (15) | 772 (38) | 624 (31) | 205 (10) | 118 (6) |
| In this community, male heads of household are expected to seek women's input when making sanitation-related decisions | 22 (1) | 184 (9) | 1122 (56) | 569 (28) | 123 (6) |
| In this community, it is acceptable for women to go to sanitation-related meetings alone | 105 (5) | 410 (20) | 1056 (52) | 337 (17) | 112 (6) |
| In this community, women are expected to be accompanied when going to certain sanitation locations | 209 (10) | 590 (29) | 766 (38) | 328 (16) | 127 (6) |
| In this community, men are typically expected to be the heads of sanitation initiatives | 257 (13) | 728 (36) | 685 (34) | 213 (11) | 137 (7) |
| In this community, it is women more often than men who are expected to accompany or support girls or other women who need assistance in managing their menstruation | 18 (1) | 63 (3) | 1000 (50) | 811 (40) | 128 (6) |
| In this community, women are expected to be accompanied when going to locations to change their menstrual materials/manage their menstruation | 327 (16) | 668 (33) | 642 (32) | 246 (12) | 137 (7) |
| It is appropriate for women to discuss menstruation-related sanitation issues in front of men | 367 (18) | 699 (35) | 559 (28) | 282 (14) | 113 (6) |
| In this community, it is acceptable for a woman to express menstruation-related concerns when her family is making decisions related to the sanitation location | 104 (5) | 370 (18) | 1006 (50) | 406 (20) | 134 (7) |
| It is not appropriate for women to discuss menstruation-related sanitation issues publicly | 288 (14) | 583 (29) | 715 (35) | 317 (16) | 117 (6) |

**Supplemental Table G.2: Response frequencies for all Relations items among women in Kampala, Uganda and Tiruchirappalli, India, organized by factor analysis results and including items removed from scales (N=2,020)**

|  | **Full Sample (%)** | | | | |
| --- | --- | --- | --- | --- | --- |
|  |  |  |  |  |  |
|  | Strongly disagree | Disagree | Agree | Strongly agree | Missing |
| ***Factor 1: Familial support for community-level participation*** | | | | |  |
| My family would encourage or help me attend a sanitation-related meeting if I wanted to go | 91 (5) | 275 (14) | 1043 (52) | 457 (23) | 154 (8) |
| My family would encourage or help me organize an initiative to improve sanitation conditions in the community | 107 (5) | 366 (18) | 1040 (51) | 340 (17) | 167 (8) |
| My family would encourage or help me to participate in a community initiative to improve sanitation | 105 (5) | 307 (15) | 1044 (52) | 401 (20) | 163 (8) |
| My family would encourage or help me to take on a leadership role in a sanitation-focused committee or organization | 198 (10) | 425 (21) | 912 (45) | 316 (16) | 169 (8) |
| ***Factor 2: Relations with service providers and local leaders*** | | | | |  |
| My interactions with local leaders or authorities about sanitation-related issues are generally free of conflict | 52 (3) | 153 (8) | 1287 (64) | 372 (18) | 156 (8) |
| When a sanitation-related problem arises in my community, I feel comfortable reporting the problem to a local leader or authority | 69 (3) | 238 (12) | 1159 (57) | 417 (21) | 137 (7) |
| When a sanitation-related problem arises, I feel comfortable reporting the problem to a service provider | 85 (4) | 292 (14) | 1071 (53) | 399 (20) | 173 (9) |
| ***Factor 3: Scolding for speaking up about sanitation issues*** | | | | | |
| If I speak up about sanitation-related concerns or problems in my household, I may be scolded or punished | 679 (34) | 1041 (52) | 134 (7) | 48 (2) | 118 (6) |
| If I speak up publicly about sanitation-related concerns or problems I may be scolded or punished by members of my household | 664 (33) | 1035 (51) | 149 (7) | 48 (2) | 124 (6) |
| If I speak up publicly about sanitation-related concerns or problems I may be scolded or punished by members of this community | 674 (33) | 1028 (51) | 137 (7) | 55 (3) | 126 (6) |
| If I speak up publicly about sanitation-related concerns or problems, I may be scolded or punished by local leaders or authorities | 696 (34) | 1029 (51) | 125 (6) | 41 (2( | 129 (6) |
| ***Items removed from scales*** |  |  |  |  |  |
| I often quarrel with members of my household about sanitation-related issues | 433 (21) | 695 (34) | 600 (30) | 177 (9) | 115 (6) |
| I often quarrel with a neighbor or other people in this community about sanitation-related issues | 429 (21) | 763 (38) | 544 (27) | 181 (9) | 103 (5) |
| There is a lot of fighting in my household about sanitation issues | 450 (22) | 987 (49) | 332 (16) | 145 (7) | 106 (5) |
| There is a lot of fighting in this community about sanitation issues | 277 (14) | 574 (28) | 645 (32) | 378 (19) | 146 (7) |
| There are quarrels or disagreements among members of my household if the toilet is not cleaned | 405 (20) | 796 (39) | 483 (24) | 188 (9) | 148 (7) |
| There are unresolved conflicts about sanitation in my household | 480 (24) | 1005 (50) | 278 (14) | 143 (7) | 114 (6) |
| There are unresolved conflicts about sanitation in this community | 255 (13) | 511 (25) | 691 (34) | 409 (20) | 154 (8) |
| I may be scolded or punished for taking too much time when I go for sanitation | 677 (34) | 1021 (51) | 151 (7) | 57 (3) | 114 (6) |
| I may be scolded or punished if the toilet/latrine is not cleaned | 552 (27) | 871 (43) | 299 (15) | 135 (7) | 163 (8) |
| I may be scolded or punished if technical issues related to the latrine/toilet, such as repairs, emptying, or construction, have not been dealt with | 630 (31) | 978 (48) | 154 (8) | 79 (4) | 179 (9) |
| When a problem arises with my sanitation location, I can discuss the problem with my family | 39 (2) | 129 (6) | 1256 (62) | 466 (23) | 130 (6) |
| When a problem arises about sanitation in our household, members of my family may blame, accuse, or criticize each other | 367 (18) | 798 (40) | 574 (28) | 143 (7) | 138 (7) |
| When a problem arises about my sanitation location, I can discuss the problem with friends or neighbors | 139 (7) | 379 (19) | 1029 (51) | 346 (17) | 127 (6) |
| When a problem arises about sanitation in this community, members of this community may blame, accuse, or criticize each other | 277 (14) | 639 (32) | 692 (34) | 261 (13) | 151 (7) |
| My interactions with sanitation-related service providers are generally free of conflict | 65 (3) | 188 (9) | 1261 (62) | 303 (15) | 203 (10) |
| I may be scolded or punished for taking too much time when I go to change my menstrual materials/manage my menstruation | 480 (24) | 617 (31) | 60 (3) | 20 (1) | 843 (42) |
| If I speak up publicly about menstruation-related sanitation concerns or problems I may be scolded or punished by members of this community | 433 (21) | 640 (32) | 77 (4) | 19 (1) | 851 (42) |
| If I speak up about menstruation-related sanitation concerns or problems in my household, I may be scolded or punished | 457 (23) | 632 (31) | 66 (3) | 21 (1) | 844 (42) |
| When I face a problem related to menstruation and my sanitation location, I can discuss the problem with members of my family | 244 (12) | 406 (20) | 386 (19) | 133 (7) | 851 (42) |
| When I face a problem related to menstruation and my sanitation location, I can discuss the problem with friends or neighbors | 270 (13) | 450 (22) | 353 (17) | 99 (5) | 848 (42) |

**Supplemental Table H:** Dropped or revised items with reasons for drop or revision, by domain

| **Dropped Items** | | | | |
| --- | --- | --- | --- | --- |
| **Resources** | | | | |
| **Item No.** | | **Item** | **Stage of Analysis** | **Reason** |
| BI03 | | In the past 30 days, I was able to access a satisfactory sanitation location when away from home. | EFA | Did not load on any factor |
| BI05 | | In the past 30 days, I have found the sanitation location I used most often to be dirty/disgusting. | CFA | Item is converse of BI04; dropping improved model statistics |
| BI06 | | In the past 30 days, I was typically able to access the resources I needed, like water, toilet paper, or soap, to clean myself after defecation (including practicing anal cleansing). | EFA | Loaded with menstruation-specific items, lowest communality (0.44) |
| BI08 | | In the past 30 days, I withheld water to avoid the urge to urinate when I knew that I would be away from home. | IRT | Low discrimination |
| BI10 | | In the past 30 days, I withheld food to avoid the urge to defecate when I knew that I would be away from home. | IRT | Easiest to answer ≥ “often” and ≥ “always” |
| BI16 | | In the past three months, I was able to access a satisfactory location when going to change my menstrual materials/manage my menstruation when I am away from home. | IRT | Low discrimination |
| H07 | | In the past 30 days, I have been injured when going to a sanitation location. | EFA | Cross-loaded on two factors |
| H11 | | In the past 30 days, I have been bitten by an animal - such as a dog, snake, or insect - when going to a sanitation location. | EFA | Cross-loaded on two factors |
| H13 | | In the past 30 days, I have felt anxiety, stress, or tension when I needed to access a sanitation location while away from home. | EFA | Cross-loaded on two factors |
| H14 | | In the past 30 days, I have felt anxiety, stress, or tension when I needed to access a sanitation location at night when at home. | EFA | Cross-loaded on two factors |
| S02 | | Women in my community face the risk of being physically harmed by men or boys if they go to a sanitation-related meeting. | EFA | Cross-loaded on two factors |
| S03 | | Women in my community face the risk of being physically harmed by men or boys if they speak up in a sanitation-related meeting. | EFA | Cross-loaded on two factors |
| S09 | | Women in my community face the risk of sexual assault when going to a sanitation-related meeting. | EFA | Cross-loaded on two factors |
| S12 | | Women in my community face the risk of someone exposing himself or spying/peeping on them when they go to sanitation locations. | EFA | Cross-loaded on two factors |
| S15 | | Women in my community consider the public toilets to be unsafe. | CFA | Substantial correlations with covariance and covariances of other items |
| S25 | | In the past 30 days, I feared I would be physically harmed by someone when I went to a sanitation location at night. | EFA | Cross-loaded on two factors |
| S26 | | In the past 30 days, I have chosen to use a different sanitation location at night for safety reasons. | IRT | Low information (IIC close to horizontal line at 0) |
| S28 | | In the past 30 days, I feared I would be sexually assaulted when I went to a sanitation location at night. | EFA | Cross-loaded on two factors |
| S30 | | In the past 30 days, I feared someone would make sexual comments or say obscene things to me when I went to a sanitation location at night. | EFA | Cross-loaded on two factors |
| S32 | | In the past 30 days, I feared someone would expose himself or spy/peep on me when I went to a sanitation location at night. | EFA | Cross-loaded on two factors |
| S37 | | In the past three months, I feared someone would make sexual comments or say obscene things to me when I went to change my menstrual materials/manage my menstruation. | EFA | Cross-loaded on two factors, low commonality |
| P06 | | In the past 30 days, I felt that my sanitation location lacked privacy because men also use this location. | EFA | Cross-loaded on two factors |
| F04 | | I have control over assets, like land, livestock, or jewelry, that I could sell to contribute to pay for household latrine/toilet construction. | EFA | Cross-loaded on two factors |
| F06 | | I do not have an income or way of acquiring money to contribute to sanitation improvements. | IRT | Revised; negative discrimination – most difficult to answer ≥ “disagree,” easiest to answer ≥ “strongly agree” |
| F10 | | Someone in my household might take money I am saving for sanitation projects or materials for their own use without first asking my permission. | IRT | Negative discrimination |
| F11 | | Someone in my household might take my money to spend on construction, improvement, or repair of a latrine/toilet without my permission. | IRT | Negative discrimination |
| F14 | | I often have to choose between paying for sanitation needs and other needs of my household. | EFA | Loaded on a factor that would become a single-item factor for non-menstruating women |
| F15 | | If I were to try to earn an income, I would not work in a place without access to a sanitation location. | EFA | Did not load on any factor |
| F16 | | I have control over money that I could use to meet my menstruation needs | IRT | Negative discrimination – most difficult to answer ≥ “agree” |
| F17 | | Someone in my household might take money I am saving for menstruation needs for their own use. | IRT | Negative discrimination – most difficult to answer ≥ “agree” |
| F18 | | I would need to ask permission before spending household money on menstruation-related expenses. | EFA | Cross-loaded on two factors |
| F20 | | I often have to choose between paying for menstruation-related needs and other needs of my household. | EFA | Loaded on a factor that would become a single-item factor for non-menstruating women |
| T01 | | I spend more time than I want on sanitation-related chores, such as cleaning latrine/toilet or assisting others in my household with their sanitation-related needs. | CFA | Low loading, substantial correlations with covariance and covariances of other items |
| T04 | | The sanitation-related needs and responsibilities of my household make me miss out on income-generating activities that I would like to do. | CFA | Low loading, substantial correlations with covariance and covariances of other items |
| SC03 | | I have friends or neighbors who I can talk to about problems related to my sanitation location. | CFA | Low loading, dropping improved model statistics |
| SC05 | | I have a friend or neighbor who would help with chores, like cooking or providing childcare, so I could tend to my sanitation needs | CFA | Entire factor dropped; other factor revised to be inclusive of these items |
| SC07 | | I have friends or neighbors who would help me with sanitation-related chores. | CFA | Entire factor dropped; other factor revised to be inclusive of these items |
| SC09 | | I have a friend or neighbor who could get small sanitation-related items if I asked them to, such as if I were too busy. | CFA | Entire factor dropped; other factor revised to be inclusive of these items |
| SC10 | | I have friends who could help me complete a sanitation-related project, such as repairing or improving my latrine/toilet. | EFA | Cross-loaded on two factors |
| SC12 | | I have friends or neighbors who would encourage or help me organize an initiative to improve sanitation conditions in the community | CFA | Low loading; dropping improved model statistics |
| SC15 | | I have friends or neighbors who make me aware of and/or encourage me to attend community meetings/events related to sanitation. | EFA | Cross-loaded on two factors |
| SC16 | | I have a friend or family member who would help me with household chores, such as cooking or childcare, so that I could attend a sanitation-related meeting. | EFA | Cross-loaded on two factors |
| SC17 | | I have a friend or family member who will accompany me to a sanitation-related meeting if I ask them to. | EFA | Cross-loaded on two factors |
| K04 | | I know of a current project or initiative to improve sanitation conditions in my community. | EFA | Cross-loaded on two factors |
| K05 | | I know of an organization or group that works to improve sanitation conditions in my community. | EFA | Cross-loaded on two factors |
| K08 | | I am knowledgeable about health issues related to sanitation | EFA | Cross-loaded on two factors |
| K12 | | I know what should and should not be put in the latrine/toilet to make sure it still works. | CFA | Low loading, dropping improved model statistics |
| K13 | | If our family had the opportunity to improve our sanitation conditions, I would know what our family needs most. | EFA | Cross-loaded on three factors |
| K17 | | I know about at least three types of toilets or sanitation options. | EFA | Did not load on any factor |
| K18 | | I feel that my knowledge helps to influence sanitation-related decisions in my household. | CFA | Low loading; dropping improved model statistics |
| K19 | | I feel that my knowledge helps to influence sanitation-related decisions in my community. | CFA | Low loading; substantial correlations with covariance and covariances of other items |
| K20 | | I know who to contact to report or resolve a sanitation-related problem in my community. | CFA | Moved to index as yes/no question |
| K21 | | I know of an individual or company that provides sanitation-related services in this community, such as latrine emptying or toilet repair. | CFA | Moved to index as yes/no question |
| CC07 | | If a man needs to urinate when away from home, he can find a place to do so more easily than a woman. | CFA | Created statistical impossibilities |
| CC08 | | If a man needs to defecate when away from home, he can find a place to do so more easily than a woman. | EFA | Did not load on any factor |
| CC12 | | Women typically need to wait a longer time than men to use a sanitation location because there are not enough facilities to meet their needs. | EFA | Cross-loaded on two factors |
| CC13 | | Men typically have to delay going to a sanitation location more often than women because of their responsibilities. | EFA | Cross-loaded on two factors |
| CC14 | | Women have more sanitation-related responsibilities than men, such as cleaning the latrine/toilet. | IRT | Created statistical impossibilities |
| CC18 | | The sanitation location I typically use makes me feel undignified. | CFA | Respondent confusion over “undignified” – removing improved model fit statistics |
| CC19 | | The sanitation conditions in my community make me feel undignified and ashamed. | CFA | Respondent confusion over “undignified” – removing improved model fit statistics |
| CC20 | | In this community, those in power - such as landlords or city authorities - typically address sanitation-related problems adequately and in a timely manner. | A priori | Directionality of the question is unclear |
| CC21 | | Sanitation locations are designed to meet the needs of menstruating women. | A priori | Directionality of the question is unclear |
| CC23 | | Sanitation locations should be improved to meet the needs of menstruating women even if this entails additional costs | EFA | Does not make conceptual sense on the factor it loaded with; low factor loading (0.43) and low communality (0.209) |
| CC24 | | The location I typically use for changing my menstrual materials/managing my menstruation makes me feel undignified | CFA | Respondent confusion over “undignified” |
| **Agency** | | | | |
| \| DM23 \| Local leaders should support women's leadership in sanitation organizations \| Did not load on any factor \| \| --- \| --- \| --- \| | | If I wanted to go to a community meeting about sanitation, I would be welcomed | CFA | Low loading; substantial correlations with covariance and covariances of other items |
| DM24 | | If there was a community meeting about sanitation issues in my neighborhood, I could attend | CFA | Low loading; substantial correlations with covariance and covariances of other items |
| DM26 | | I would feel comfortable expressing my opinion about sanitation issues at a community meeting when only women are present | EFA | Cross-loaded on three factors |
| DM38 | | If I spoke up in a household discussion about sanitation issues, it is likely that members of my household would listen. | CFA | Low loading, substantial correlations with covariance and covariances of other items |
| DM39 | | If my household had a major decision to make about sanitation, such as construction or large repairs, I could influence that decision. | EFA | Cross-loaded on two factors |
| DM40 | | If my household had decisions to make about latrine/toilet repairs or enhancements, like new floor tiles, doors, locks, or lights, I could influence that decision | EFA | Cross-loaded on two factors |
| DM41 | | If my household had decisions to make about small sanitation-related purchases, like soap, toilet paper, etc., I could influence that decision | EFA | Cross-loaded on two factors |
| DM42 | | I can influence how my household will clean and maintain the sanitation environment/facility | CFA | Low loading, substantial correlations with covariance and covariances of other items |
| DM47 | | I would feel comfortable expressing my opinion about menstruation-related sanitation issues at a community meeting when men are present. | EFA | Cross-loaded on two factors; decision to drop all community menstruation items |
| DM48 | | I would feel comfortable expressing my opinion about menstruation-related sanitation issues at a community meeting when only women are present. | EFA | Loaded onto household factor; decision to drop all community menstruation items |
| DM49 | | If my community had decisions to make about making toilets more female-friendly, such as installing rubbish bins for menstrual materials, I could influence those decisions. | EFA | Cross-loaded on two factors; decision to drop all community menstruation items |
| L16 | | Local leaders should support women's leadership in sanitation organizations | EFA | Did not load on any factor |
| L20 | | I would listen to a knowledgeable woman's advice about sanitation-related issues even if she were not in an official leadership role | EFA | Too many hypothetical clauses in question |
| CA15 | | If a sanitation project does not directly benefit me, but has benefits for many others in the community, I would be likely to contribute time/labor, money, or other resources to the project | EFA | Did not load on any factor |
| CA18 | | This community has good leaders for sanitation-related initiatives and projects | CFA | Low loading, substantial correlations with covariance and covariances of other items |
| CA21 | | If I have a sanitation-related problem, I could trust that members of my community would not shame me | CFA | Low loading, substantial correlations with covariance and covariances of other items |
| M04 | | If I need to access water for sanitation-related needs, such as water for flushing, washing feces-soiled children's clothing, or managing my menstruation, I can go to the place where my household collects water | EFA | Cross-loaded on two factors |
| M16 | | If I need to access water for menstruation-related needs, such as washing myself or washing my menstrual materials, I can go to the place where my household collects water: | EFA | Cross-loaded on two factors |
|  | **Institutional Structures** | | | |
| \| N23 \| Local leaders should support women's leadership in sanitation organizations \| Did not load on any factor \| \| --- \| --- \| --- \| | | In this community, it is acceptable for a wife to decide to go to a sanitation-related meeting without first consulting her husband | EFA | Low communality and loading; did not fit conceptually in factor |
| N25 | | In this community, it is acceptable for women to attend sanitation-related meetings | EFA | Cross-loaded on two factors |
| N26 | | In this community, men are expected to make the final decision on major sanitation-related decisions for the household, such as construction, repairs, or upgrades | EFA | Did not load on any factor |
| N27 | | In this community, it is acceptable for husbands to make important decisions about the household's sanitation facility without asking their wives' opinions | EFA | Did not load on any factor |
| N28 | | In this community, male heads of household are expected to seek women's input when making sanitation-related decisions | EFA | Loaded on factor where it does not make conceptual sense |
| N31 | | In this community, it is acceptable for women to go to sanitation-related meetings alone | EFA | Did not load on any factor |
| N32 | | In this community, women are expected to be accompanied when going to certain sanitation locations | IRT | Negative discrimination |
| N33 | | In this community, men are typically expected to be the heads of sanitation initiatives | EFA | Low commonality; loaded on factor where it does not make conceptual sense |
| N38 | | In this community, it is women more often than men who are expected to accompany or support girls or other women who need assistance in managing their menstruation. | IRT | Negative discrimination |
| R01 | | I often quarrel with members of my household about sanitation-related issues | EFA | Cross-loaded on two factors |
| R02 | | I often quarrel with a neighbor or other people in this community about sanitation-related issues | EFA | Cross-loaded on two factors |
| R03 | | There is a lot of fighting in my household about sanitation issues | EFA | Cross-loaded on two factors |
| R04 | | There is a lot of fighting in this community about sanitation issues | CFA | Dropped entire factor; did not correlate/fit with other factors |
| R05 | | There are quarrels or disagreements among members of my household if the toilet is not cleaned. | EFA | Cross-loaded on two factors |
| R06 | | There are unresolved conflicts about sanitation in my household | EFA | Cross-loaded on two factors |
| R07 | | There are unresolved conflicts about sanitation in this community. | CFA | Dropped entire factor; did not correlate/fit with other factors |
| R08 | | I may be scolded or punished for taking too much time when I go for sanitation. | EFA | Cross-loaded on two factors |
| R09 | | I may be scolded or punished if the toilet/latrine is not cleaned | EFA | Loaded cleanly but on inappropriate factor in Round 1; cross-loaded in Round 2; likely to be irrelevant for women using public toilets |
| R10 | | I may be scolded or punished if technical issues related to the latrine/toilet, such as repairs, emptying, or construction, have not been dealt with. | EFA | Loaded cleanly but on inappropriate factor in Round 1; cross-loaded in Round 2; likely to be irrelevant for women using public toilets |
| R19 | | When a problem arises with my sanitation location, I can discuss the problem with my family | EFA | Cross-loaded on two factors |
| R20 | | When a problem arises about sanitation in our household, members of my family may blame, accuse, or criticize each other | EFA | Cross-loaded on two factors |
| R21 | | When a problem arises about my sanitation location, I can discuss the problem with friends or neighbors | EFA | Cross-loaded on two factors |
| R22 | | When a problem arises about sanitation in this community, members of this community may blame, accuse, or criticize each other | CFA | Dropped entire factor; did not correlate/fit with other factors |
| R23 | | My interactions with sanitation-related service providers are generally free of conflict | CFA | Low loading |
| R27 | | I may be scolded or punished for taking too much time when I go to change my menstrual materials/manage my menstruation | EFA | Cross-loaded on two factors |
| R29 | | If I speak up publicly about menstruation-related sanitation concerns or problems I may be scolded or punished by members of this community | EFA | Cross-loaded on two factors |
| **Revised Items** | | | | |
| **Item No.** | | **Item** | **Stage of Analysis** | **Reason** |
| L17 | | I generally trust sanitation groups/organizations with men as the head of the organization/group more than those with women as the head | IRT | Revised; negative discrimination; most difficult to answer ≥ "disagree" and ≥ "agree" |
| N40 | | It is not appropriate for women to discuss menstruation-related sanitation issues publicly. | IRT | Negative discrimination |
| R30 | | When I face a problem related to menstruation and my sanitation location, I can discuss the problem with members of my family | IRT | Negative discrimination; most difficult to answer ≥ "disagree"; easiest to answer ≥ "strongly agree" |
| R31 | | When I face a problem related to menstruation and my sanitation location, I can discuss the problem with friends or neighbors | IRT | Negative discrimination; most difficult to answer ≥ "disagree"; easiest to answer ≥ "strongly agree" |
|  | |  |  |  |

**Supplemental Table I.** Results of EFA and CFA for menstruation-related items

| Sub-domain: Factor Name | EFA* | CFA | | | | |
| --- | --- | --- | --- | --- | --- | --- |
|  | **Pattern Coefficients** | **Pattern Coefficients** | **RMSEA (90% CI)** | **SRMR** | **CFI** | **TLI** |
| Resources | | | | | | |
| Health: Menstruation Related Fear and Stress |  |  | 0.102 (0.044, 0.176) | 0.005 | 1.000 | 0.999 |
| In the past three months, I have experienced stress or tension when I needed to access a location to change my menstrual materials/manage my menstruation. | 0.917 | 0.962 |  |  |  |  |
| In the past three months, I have felt stress or tension when changing my menstrual materials/managing my menstruation. | 0.958 | 1.000 |  |  |  |  |
| In the past three months, I have felt scared when changing my menstrual materials/managing my menstruation. | 0.934 | 0.918 |  |  |  |  |
| Bodily Integrity: Satisfaction with menstrual hygiene management |  |  | 0.288 (0.268, 0.310) | 0.168 | 0.956 | 0.935 |
| In the past three months, I felt satisfied with the location I use most often to change my menstrual materials/manage my menstruation. | 0.918 | 1.000 |  |  |  |  |
| In the past three months, I was able to access a satisfactory location when going to change my menstrual materials/manage my menstruation when I am at home. | 0.883 | 0.916 |  |  |  |  |
| In the past three months, I was typically able to access the resources I needed, like water or soap, to clean myself when using a sanitation location during menstruation. | 0.642 | 0.578 |  |  |  |  |
| In the past three months, I have had to delay changing my menstrual material during the daytime when at home because I did not have access to a satisfactory sanitation location. | 0.879 | 0.770 |  |  |  |  |
| In the past three months, I have had to delay changing my menstrual materials when I was away from home because I did not have access to a satisfactory sanitation location. | 0.722 | 0.625 |  |  |  |  |
| In the past three months, I have had to delay changing my menstrual materials at night when at home because I did not have access to a satisfactory sanitation location. | 0.886 | 0.841 |  |  |  |  |
| Safety and Security: Perceptions of own safety related to menstruation |  |  | 0.149 (0.088, 0.221) | 0.014 | 0.997 | 0.991 |
| In the past three months, I have felt safe in the place where I typically go to change my menstrual materials/manage my menstruation when I am at home. | 0.959 | 1.000 |  |  |  |  |
| In the past three months, I have felt safe in the place where I typically go to change my menstrual materials/manage my menstruation when I am away from home. | 0.784 | 0.817 |  |  |  |  |
| In the past three months, the location I use to change my menstrual materials/manage my menstruation during the day is safe at night. | 0.914 | 0.884 |  |  |  |  |
| Privacy: Privacy for menstrual hygiene management |  |  | 0.160 (0.139, 0.182) | 0.041 | 0.986 | 0.980 |
| In the past three months, I have worried that someone would see me changing my menstrual materials/managing my menstruation while using a sanitation location. | 0.905 | 0.912 |  |  |  |  |
| In the past three months, I have worried that someone would see me carrying menstrual materials on my way to a sanitation location. | 0.952 | 0.902 |  |  |  |  |
| In the past three months, I have had to use locations to change my menstrual materials/manage my menstruation that were not private enough for me. | 0.859 | 0.938 |  |  |  |  |
| In the past three months, I have had difficulty finding a private place to change my menstrual materials/manage my menstruation. | 0.855 | 0.913 |  |  |  |  |
| In the past three months, I have worried that someone will see my menstrual blood in the sanitation location that I use to change my menstrual material. | 0.984 | 1.000 |  |  |  |  |
| In the past three months, I have worried that someone would see me washing my menstrual materials in a sanitation location. | 0.829 | 0.896 |  |  |  |  |
| Social capital: Social capital for meeting menstruation-related needs |  | ** |  |  |  |  |
| I have someone in my household who could get menstruation-related items if I asked them to, such as if I were too busy. | 0.815 |  |  |  |  |  |
| I have a friend or neighbor who could get menstruation-related items if I asked them to, such as if I were too busy. | 0.672 |  |  |  |  |  |
| Time: Time for meeting menstruation-related needs |  |  | 0.151 (0.090, 0.223) | 0.010 | 0.994 | 0.983 |
| I would like to have more time to meet my menstruation-related needs. | 0.668 | 0.758 |  |  |  |  |
| I often have to wake up earlier than I want to access a location to manage my menstruation. | 1.001 | 1.000 |  |  |  |  |
| I often miss out on activities I would like to do because of time spent taking care of my menstruation-related needs. | 0.504 | 0.693 |  |  |  |  |
| Agency | | | | | | |
| Decision-Making: Ability to participate in menstruation-related decisions |  |  | 0.099 (0.051, 0.158) | 0.009 | 0.997 | 0.992 |
| I would feel comfortable expressing my opinion about menstruation-related sanitation issues in household discussions. | 0.605 | 0.661 |  |  |  |  |
| If my household had decisions to make about making the toilet more female-friendly, such as buying a rubbish bin for menstrual materials, I could influence that decision | 0.961 | 1.000 |  |  |  |  |
| If my household had decisions to make about making the toilet more female-friendly, such as buying a rubbish bin for menstrual materials, I could have the final say on that decision. | 0.661 | 0.730 |  |  |  |  |
| Freedom of movement: Freedom of movement for meeting menstruation-related needs |  |  | 0.101 (0.042, 0.175) | 0.025 | 0.994 | 0.981 |
| If I need to change my menstrual materials when at home during the day, I can go: | 0.839 | 0.851 |  |  |  |  |
| If I need to change my menstrual materials when away from home during the day, I can go: | 0.764 | 0.695 |  |  |  |  |
| If I need to change my menstrual materials at night, I can go to my household's daytime sanitation location: | 0.915 | 1.000 |  |  |  |  |
| Institutional Structures | | | | | | |
| Norms: Acceptability of women discussing menstruation-related issues |  |  | 0.466 (0.414, 0.520) | 0.067 | 0.782 | 0.345 |
| It is appropriate for women to discuss menstruation-related sanitation issues in front of men. | 0.596 | -0.569 |  |  |  |  |
| In this community, it is acceptable for a woman to express menstruation-related concerns when her family is making decisions related to the sanitation location. | 0.494 | -0.521 |  |  |  |  |
| It is not appropriate for women to discuss menstruation-related sanitation issues publicly. | -0.640 | 1.000 |  |  |  |  |
| Relations: Ability to discuss menstruation-related items |  |  | 0.027 (0.000, 0.116) | 0.005 | 1.000 | 0.999 |
| If I speak up about menstruation-related sanitation concerns or problems in my household, I may be scolded or punished. | 0.570 | -0.375 |  |  |  |  |
| When I face a problem related to menstruation and my sanitation location, I can discuss the problem with members of my family | 0.801 | 0.780 |  |  |  |  |
| When I face a problem related to menstruation and my sanitation location, I can discuss the problem with friends or neighbors | 0.887 | 1.000 |  |  |  |  |

* Menstruation-related items were included in the EFA models for each scale. Results, including fit statistics (Root Mean Squared Error of Approximation (RMSEA), Comparative Fit Index (CFI), Tucker-Lewis Index (TLI), and Standardized Root Mean Squared Residual (SRMR)), for all EFA models are shown in Table 2.

** Cannot fit a CFA for Social Capital as the factor only has two items; a minimum of three items are required for model convergence.

**Supplemental Table J: Results of assessment of construct, criterion, and known groups validity**

**Supplemental Table J.1:** Construct validity

| Scale Name | Questions / Measures for Validity | Spearman rho | P-value |
| --- | --- | --- | --- |
| Resources | | | |
| Safety and security | Index of "I know a woman in my community who has..." | 0.36363 | <0.0001 |
| Financial and productive assets | Survey questions on asset ownership (split by country due to differences in assets) | | |
|  | India | 0.13786 | <0.0001 |
|  | Uganda | -0.03783 | 0.2579 |
| Time | Survey questions on time spent on tasks related to sanitation: | | |
|  | How many times a week do you have to go to the water source to  collect water for sanitation needs? | 0.04601 | 0.1237 |
|  | How long does it take for you to collect water? | 0.01615 | 0.5891 |
|  | How many minutes do you need to walk to your sanitation  location? | 0.11031 | 0.0001 |
| Knowledge | Survey questions on access and use of media: | | |
|  | Do you read a newspaper or magazine at least once a week, less  than once a week, or not at all? | -0.17823 | <0.0001 |
|  | Do you listen to the radio at least once a week, less than once a  week, or not at all? | -0.11295 | <0.0001 |
|  | Do you watch television at least once a week, less than once a  week, or not at all? | -0.06624 | <0.0001 |
| Agency | | | |
| Leadership | Index of women's assumption of and participation in formal and informal leadership positions | 0.06925 | 0.2697 |
| Decision-making | Index documenting women influencing and making decision about sanitation in the home | 0.39861 | <0.0001 |
|  | Index documenting women influencing and making decision about sanitation outside of the home | 0.40939 | <0.0001 |
|  | Survey questions on general decision making (Response options: No, not at all comfortable; Yes, but with a great deal of difficulty; Yes, but with a little difficulty; Yes, fairly comfortable; Yes, very comfortable): | | |
|  | Do you feel comfortable expressing your opinion at a community  meeting where only women are present? | 0.06617 | 0.0092 |
|  | Do you feel comfortable expressing your opinion at a community  meeting where both men and women are present? | 0.32034 | <0.0001 |
| Collective   action | Index of women's participation in collective action around sanitation | 0.42774 | <0.0001 |
| Freedom of    movement | Index of women's freedom of movement for sanitation | 0.08771 | 0.0001 |

**Supplemental Table J.2:** Criterion validity

| Scale Name | Questions / Measures for Validity | Spearman rho | P-value |
| --- | --- | --- | --- |
| Resources | | | |
| Health | WHO 5 Well-being Index | 0.08351 | 0.0007 |
|  | Patient Health Questionnaire - 4 | 0.0694 | 0.0055 |
| Financial and   productive assets | One question from SEEP Network Assessment Tool for Microfinance Practitioners: When you want or need to buy things like food or clothing for yourself or your family, which of the following answers best describes your situation?  You have your own money so you can usually buy what you need  You occasionally have to get the money from your husband or someone else in the household  You always have to get the money from your husband or someone else in the household | -0.42301 | <0.001 |
| Social capital | Eight items from the 12-item Multidimensional Scale of Perceived Social Support (Response options: Completely disagree, Mildly disagree, Neither agree nor disagree, Mildly agree, Completely agree): 1. My family really tries to help me. 2. I get the emotional help and support I need from my family. 3. My friends really try to help me. 4. I can count on my friends when things go wrong. 5. I can talk about my problems with my family. 6. I have friends with whom I can share my joys and sorrows. 7. My family is willing to help me make decisions. 8. I can talk about my problems with my friends. | 0.2869 | <0.001 |
| Critical consciousness  - Scale 1 | General Self-Efficacy - 6 Scale | 0.2085 | <0.001 |
| Agency | | | |
| Leadership | Empowerment Scale in Community Organizing Emotional empowerment dimension, subscale 1 (Response options: Strongly disagree, Disagree, Neither disagree nor agree, Agree, Strongly agree): 1. I am often a leader in groups. 2. I would prefer to be a leader rather than a follower. 3. I would rather someone else took over the leadership role when I’m involved in a group project. | -0.05283 | 0.0592 |
| Collective action | Community Capacity Instrument, Connections dimension (Response options: Strongly disagree, Disagree, Neither disagree nor agree, Agree, Strongly agree): 1. Most people in the community can be trusted 2. I don't feel welcome to join local groups and activities 3. Residents are friendly and inclusive of newcomers 5. I have little in common with most people who live here 4. This neighborhood is a close-knit community | 0.28532 | <0.001 |
| Freedom of  movement | Mobility measure, first subscale from the India Human Development Survey - 2 (Response options: no, must inform, yes) Please tell us whether you have to ask permission of your husband or a senior family member to go… to the local health center? to the home of relatives or friends [in the village / neighborhood]? a short distance by train or bus? | 0.28352 | <0.001 |
| Institutional structures | | | |
| Norms | Four items from the Gender equity scale (Response options: Strongly disagree, Disagree, Agree, Strongly agree): 1. A woman can talk to men other than her husband 6. A woman can participate in community activities if she wishes to 7. The status of women is lower than that of men 9. A woman should finish all the household work before taking rest | -0.18712 | <0.001 |
| Relations | Three items from the Brief Family Relationship Scale (Response options: Not at all; A little; Somewhat; Quite a bit; A lot): 7. In our family there is a feeling of togetherness 8. In our family we sometimes tell each other about our personal problems 9. In our family we lose our tempers a lot | 0.27603 | <0.001 |

**Supplemental Table J.3:** Known groups validity

| Scale Name | Questions / Measures for Validity | t-value or F Value (Degrees of Freedom) | P-value |
| --- | --- | --- | --- |
| Resources | | | |
| Bodily integrity | Survey questions on privacy of sanitation location: | | |
|  | Is it possible for someone to see you while you are using  this sanitation location? | -5.20 | <0.001 |
|  | Is this sanitation location lockable from the inside? | 3.45 | 0.0013 |
|  | Is there sufficient room inside this sanitation location? | 2.72 | 0.0069 |
|  | Is this sanitation location located in a private place? | -1.52 | 0.1296 |
| Health | One item from the Patient-Reported Outcomes Measurement Information System (PROMIS) global health subscale: In general, how would you rate your physical health? (Response options: Excellent, Very Good, Good, Fair, Poor) | 7.43(4) | <0.001 |
| Privacy | Survey questions on privacy of sanitation location: | | |
|  | Is it possible for someone to see you while you are using  this sanitation location? | 4.39 | <0.001 |
|  | Is this sanitation location lockable from the inside? | -3.04 | 0.0043 |
|  | Is there sufficient room inside this sanitation location? | -3.85 | 0.0003 |
|  | Is this sanitation location located in a private place? | 0.15 | 0.8794 |
| Financial and   productive assets | One question from SEEP Network Assessment Tool for Microfinance Practitioners: When you want or need to buy things like food or clothing for yourself or your family, which of the following answers best describes your situation?  You have your own money so you can usually buy what you need  You occasionally have to get the money from your husband or someone else in the household  You always have to get the money from your husband or someone else in the household | 176.43(2) | <0.001 |
| Knowledge | Survey questions on completed schooling: | | |
|  | Did you ever attend school? | 5.62 | <0.001 |
|  | Have you ever used the internet? | 6.23 | <0.001 |
|  | Questions on access and use of media: | | |
|  | Do you read a newspaper or magazine at least once a week,  less than once a week, or not at all? | 24.03(2) | <0.001 |
|  | Do you listen to the radio at least once a week, less than  once a week, or not at all? | 12.93(2) | <0.001 |
|  | Do you watch television at least once a week, less than  once a week, or not at all? | 6.35(2) | 0.0018 |
| Agency | | | |
| Decision making | Three items from the Demographic and Health Surveys (Response options: Respondent; Husband; Respondent and husband jointly; Someone else; Other): | | |
|  | 1. Who usually makes decisions about health care for   yourself? | 44.77(3) | <0.001 |
|  | 1. Who usually makes decisions about making major   household purchases? | 37.15(3) | <0.001 |
|  | 1. Who usually makes decisions about visits to your family or relatives? | 35.60(3) | <0.001 |
| Freedom of movement | Mobility measure, first subscale from the India Human Development Survey - 2 (Response options: no, must inform, yes) Please tell us whether you have to ask permission of your husband or a senior family member to go… | | |
|  | to the local health center? | 73.55(2) | <0.001 |
|  | to the home of relatives or friends [in the village /  neighborhood]? | 68.50(2) | <0.001 |
|  | a short distance by train or bus? | 74.10(2) | <0.001 |

**Supplementary Table K: Scoring Results from Bifactor Models**

| **Scale Name** | **ω_H_/ω Ratio** |
| --- | --- |
| **Resources** |  |
| Health | 1.00 |
| Bodily integrity | 0.97 |
| Safety and security | 0.98 |
| Privacy | * |
| Financial and productive assets | 1.00 |
| Social capital | 1.01 |
| Time | 1.00 |
| Knowledge | 1.00 |
| Critical consciousness (SCALE 1) | 0.99 |
| Critical consciousness (SCALE 2) | 1.00 |
| **Agency** |  |
| Leadership | 1.01 |
| Decision making | 1.00 |
| Collective action | 1.00 |
| Freedom of movement | 0.96 |
| **Institutional structures** |  |
| Norms | 0.99 |
| Relations | 1.01 |
| * Bifactor model was not fit for the single-factor Privacy scale as bifactor models require at least 2 underlying factors | |

**Supplemental Table L: Correlation matrix for Pearson correlations between scored scales**

|  | Collective Action | Relations | Norms | Social Capital | Knowledge | Time | Financial & Productive Assets | Critical Conscious-ness (1) | Critical Conscious-ness (2) | Privacy | Health | Safety | Decision Making | Bodily Integrity | Leader-ship | Freedom of Movement |
| --- | --- | --- | --- | --- | --- | --- | --- | --- | --- | --- | --- | --- | --- | --- | --- | --- |
| Collective Action | 1.000 | 0.461 | 0.221 | 0.620 | 0.538 | 0.184 | 0.270 | 0.452 | 0.023 | 0.100 | 0.226 | 0.083 | 0.651 | 0.181 | 0.260 | 0.029 |
| Relations | 0.461 | 1.000 | 0.342 | 0.378 | 0.294 | 0.301 | 0.285 | 0.338 | 0.014 | 0.062 | 0.085 | 0.203 | 0.529 | 0.044 | 0.426 | 0.092 |
| Norms | 0.221 | 0.342 | 1.000 | 0.156 | 0.064 | 0.088 | 0.099 | 0.165 | 0.342 | 0.033 | 0.053 | 0.010 | 0.168 | 0.033 | 0.259 | 0.038 |
| Social Capital | 0.620 | 0.378 | 0.156 | 1.000 | 0.526 | 0.184 | 0.252 | 0.494 | 0.060 | 0.134 | 0.201 | 0.108 | 0.623 | 0.131 | 0.212 | 0.024 |
| Knowledge | 0.538 | 0.294 | 0.064 | 0.526 | 1.000 | 0.174 | 0.412 | 0.489 | 0.140 | 0.124 | 0.138 | 0.124 | 0.557 | 0.084 | 0.169 | 0.024 |
| Time | 0.184 | 0.301 | 0.088 | 0.184 | 0.174 | 1.000 | 0.056 | 0.092 | 0.057 | 0.290 | 0.411 | 0.545 | 0.100 | 0.391 | 0.302 | 0.036 |
| Financial & Productive Assets | 0.270 | 0.285 | 0.099 | 0.252 | 0.412 | 0.056 | 1.000 | 0.293 | 0.089 | 0.051 | 0.068 | 0.018 | 0.422 | 0.036 | 0.096 | 0.135 |
| Critical Conscious-ness (1) | 0.452 | 0.338 | 0.165 | 0.494 | 0.489 | 0.092 | 0.293 | 1.000 | 0.010 | 0.143 | 0.153 | 0.071 | 0.513 | 0.059 | 0.198 | 0.013 |
| Critical Conscious-ness (2) | 0.023 | 0.014 | 0.342 | 0.060 | 0.140 | 0.057 | 0.089 | 0.010 | 1.000 | 0.042 | 0.029 | 0.026 | 0.070 | 0.002 | 0.069 | 0.040 |
| Privacy | 0.100 | 0.062 | 0.033 | 0.134 | 0.124 | 0.290 | 0.051 | 0.143 | 0.042 | 1.000 | 0.610 | 0.549 | 0.161 | 0.499 | 0.078 | 0.081 |
| Health | 0.226 | 0.085 | 0.053 | 0.201 | 0.138 | 0.411 | 0.068 | 0.153 | 0.029 | 0.610 | 1.000 | 0.569 | 0.232 | 0.647 | 0.013 | 0.036 |
| Safety | 0.083 | 0.203 | 0.010 | 0.108 | 0.124 | 0.545 | 0.018 | 0.071 | 0.026 | 0.549 | 0.569 | 1.000 | 0.055 | 0.439 | 0.263 | 0.136 |
| Decision Making | 0.651 | 0.529 | 0.168 | 0.623 | 0.557 | 0.100 | 0.422 | 0.513 | 0.070 | 0.161 | 0.232 | 0.055 | 1.000 | 0.190 | 0.328 | 0.050 |
| Bodily Integrity | 0.181 | 0.044 | 0.033 | 0.131 | 0.084 | 0.391 | 0.036 | 0.059 | 0.002 | 0.499 | 0.647 | 0.439 | 0.190 | 1.000 | 0.066 | 0.003 |
| Leadership | 0.260 | 0.426 | 0.259 | 0.212 | 0.169 | 0.302 | 0.096 | 0.198 | 0.069 | 0.078 | 0.013 | 0.263 | 0.328 | 0.066 | 1.000 | 0.138 |
| Freedom of Movement | 0.029 | 0.092 | 0.038 | 0.024 | 0.024 | 0.036 | 0.135 | 0.013 | 0.040 | 0.081 | 0.036 | 0.136 | 0.050 | 0.003 | 0.138 | 1.000 |

**Supplementary Table M: Resources domain scales that have been finalized after Phase 2, by subdomain and factor (5 of 10 scales in Resources domain).**

| **Finalized Resources Scales** | | |
| --- | --- | --- |
| **Subdomain:** Financial and Productive Assets  **Response Options:** Strongly agree; agree; disagree; strongly disagree | | |
| **Factor** | **Factor Name** | **Item** |
| F1 | Control over money for sanitation | I have control over money that I could use to contribute to a community sanitation project. |
| F1 | Control over money for sanitation | I have control over money that I could use to pay for household latrine/toilet improvements or repairs. |
| F1 | Control over money for sanitation | I have control over money that I could use to pay for household latrine/toilet construction. |
| F2 | Ability to acquire money for sanitation | I could acquire money to build a household latrine/toilet by selling or renting something I own or by earning money through work. |
| F2 | Ability to acquire money for sanitation | I could acquire money to improve or repair a household latrine/toilet by selling or renting something I own or by earning money through work. |
| F2 | Ability to acquire money for sanitation | I could acquire money to build a household latrine/toilet by accessing credit or participating in a savings group. |
| F3 | Inadequate finances to meet basic sanitation needs | I would need to ask permission before spending household money on small sanitation-related expenses, such as toilet paper, soap, or pay-per-use latrines. |
| F3 | Inadequate finances to meet basic sanitation needs | I depend on someone else to pay for small sanitation-related expenses, such as toilet paper, soap, or pay-per-use latrines. |
| **Subdomain:** Time  **Response Options:** Strongly agree; agree; disagree; strongly disagree | | |
| **Factor** | **Factor Name** | **Item** |
| F1 | Time on sanitation-related responsibilities | The sanitation-related needs and responsibilities of my household prevent me from completing other household work. |
| F1 | Time on sanitation-related responsibilities | The sanitation-related needs and responsibilities of my household require that I often have to wake up early than I want. |
| F1 | Time on sanitation-related responsibilities | The sanitation-related needs and responsibilities of my household often make me mis out on other activities that I would like to do. |
| F2 | Time meeting personal sanitation needs | It often takes too much time to access and use my sanitation location. |
| F2 | Time meeting personal sanitation needs | I often have to rush when I am using my sanitation location. |
| F2 | Time meeting personal sanitation needs | I often have to wake up earlier than I want to access a sanitation location. |
| **Subdomain:** Knowledge  **Response Options:** Strongly agree; agree; disagree; strongly disagree | | |
| **Factor** | **Factor Name** | **Item** |
| F1 | Knowledge and skills related to community sanitation | I have skills – such as budgeting, organization, or mobilizing people in my community – to contribute to sanitation related projects in my community. |
| F1 | Knowledge and skills related to community sanitation | I know how decisions about sanitation are made in my community. |
| F1 | Knowledge and skills related to community sanitation | I know how to manage money for a sanitation project in my community. |
| F2 | Knowledge related to personal sanitation repairs and maintenance | If my toilet/latrine needed repairs, I would know how to get it fixed. |
| F2 | Knowledge related to personal sanitation repairs and maintenance | I know how to make minor repairs or improvements to a latrine/toilet, like unclogging, replacing a lightbulb, or fixing a door. |
| F3 | Knowledge to influence community-level sanitation decisions | I have enough knowledge to contribute to decisions on sanitation in my community. |
| F3 | Knowledge to influence community-level sanitation decisions | I am knowledgeable about my community’s sanitation issues. |
| F3 | Knowledge to influence community-level sanitation decisions | If my community had the opportunity to improve our sanitation conditions, I would have ideas about what the community needs most. |
| F4 | Knowledge and skills to influence household-level sanitation decisions | I have skills – such as budgeting and organization – to contribute to sanitation related projects in my household. |
| F4 | Knowledge and skills to influence household-level sanitation decisions | I know how to manage money for a sanitation project in my household. |
| **Subdomain:** Critical Consciousness Scale 1 (self-efficacy)  **Response Options:** Strongly agree; agree; disagree; strongly disagree | | |
| **Factor** | **Factor Name** | **Item** |
| F1 | Ability to create change in the community | I feel I can change sanitation conditions in my community if I want to. |
| F1 | Ability to create change in the community | I feel like I can influence sanitation behaviors of others in my community, such as encouraging proper latrine use and maintenance, proper disposal of sanitation napkins, etc. |
| F2 | Ability to create change at home | I feel I can change sanitation conditions in my household or compound if I want to. |
| F2 | Ability to create change at home | I feel like I can influence sanitation behaviors of members of my household. |
| F2 | Ability to create change at home | If I had a problem related to sanitation, I could probably think of a solution. |
| F2 | Ability to create change at home | If I had a problem related to sanitation, I believe I could solve it. |
| **Subdomain:** Critical Consciousness Scale 2 (awareness of inequalities)  **Response Options:** Strongly agree; agree; disagree; strongly disagree | | |
| **Factor** | **Factor Name** | **Item** |
| F1 | Inequalities in ability to meet sanitation needs | Women have problems that men do not have when accessing or using a sanitation location. |
| F1 | Inequalities in ability to meet sanitation needs | It is unfair when women have problems accessing or using a sanitation location but men do not have those problems. |
| F1 | Inequalities in ability to meet sanitation needs | Women typically have to delay going to a sanitation location more often than men because of their responsibilities. |
| F2 | Inequalities in sanitation-related decision-making | Women are less involved than men in making decisions about sanitation in the community. |
| F2 | Inequalities in sanitation-related decision-making | It is fair for men to have the final say when making sanitation-related decisions in the household. |
| F2 | Inequalities in sanitation-related decision-making | It is fair for men to have the final say when making sanitation-related decisions for the community. |

**Supplementary Table N: Agency domain scales that have been finalized after Phase 2, by subdomain and factor (3 of 4 scales in Agency domain).**

| **Finalized Agency Scales** | | |
| --- | --- | --- |
| **Subdomain:** Collective Action  **Response Options:** Strongly agree; agree; disagree; strongly disagree | | |
| **Factor** | **Factor Name** | **Item** |
| F1 | Shared goals and interests | Community members and I share common goals for improving sanitation in this community |
| F1 | Shared goals and interests | The sanitation-related goals I share with my community motivate me to work with others, even when that is challenging |
| F2 | Sense of trust and community | I am confident that members of my community will work with one another to achieve sanitation related goals. |
| F2 | Sense of trust and community | People in this community would be willing to contribute time/labor, money, or other resources toward common sanitation goals |
| F2 | Sense of trust and community | If I need help with a sanitation-related project, there are people in this community who I could trust to help me. |
| F3 | Sense of mutual support | If there was a sanitation problem in this community, it is likely that people would cooperate to try to solve the problem. |
| F3 | Sense of mutual support | If there is a problem that affects the entire community, people in this community would help each other to address the issue. |
| F3 | Sense of mutual support | Most people in this community would be willing to help each other with sanitation-related projects |
| **Subdomain:** Decision-making  **Response Options:** Strongly agree; agree; disagree; strongly disagree | | |
| **Factor** | **Factor Name** | **Item** |
| F1 | Ability to speak up in community-level decision-making | I would feel comfortable expressing my opinion about sanitation issues at a community meeting when men are present |
| F1 | Ability to speak up in community-level decision-making | If I spoke up in a community meeting about sanitation issues, it is likely that some others would listen |
| F1 | Ability to speak up in community-level decision-making | If I shared my opinion about sanitation issues with local leaders, NGOs, or government officials, it is likely that they would listen. |
| F2 | Ability to influence community-level decision-making | If my community had a major decision to make about sanitation, such as constructing public toilets, I could influence that decision. |
| F2 | Ability to influence community-level decision-making | If my community had decisions to make about latrine/toilet repairs or enhancements, like new floor tiles, doors, locks, or lights, I could influence these decisions |
| F2 | Ability to influence community-level decision-making | If my community had decisions to make about maintenance or cleaning of latrines/toilets, I could influence those decisions. |
| F3 | Ability to participate in household decision-making | If my household was making a decision about sanitation-related issues, I could be present for the discussion. |
| F3 | Ability to participate in household decision-making | If my household was making a decision about sanitation-related issues, I would be welcome to participate in the discussion. |
| F3 | Ability to participate in household decision-making | I would feel comfortable expressing my opinion about sanitation issues in household discussions. |
| F4 | Ability to make household-level decisions | If my household had a major decision to make about sanitation, such as construction or large repairs, I could independently make that decision. |
| F4 | Ability to make household-level decisions | If my household had decisions to make about latrine/toilet repairs or enhancements, like new floor tiles, doors, locks, or lights, I could independently make that decision. |
| F5 | Ability to make small household-level decisions | If my household had decisions to make about small sanitation-related purchases, like soap toilet paper, etc., I could independently make those decisions. |
| F5 | Ability to make small household-level decisions | I can independently make decisions about how my household will clean and maintain the sanitation environment/facility. |
| **Subdomain:** Freedom of Movement  **Response** Options: Alone without telling anyone; alone if I tell someone; alone with permission; only with accompaniment | | |
| **Factor** | **Factor Name** | **Item** |
| F1 | Freedom of movement for meeting personal sanitation needs | If I need to use a sanitation location when at home during the day, I can go: |
| F1 | Freedom of movement for meeting personal sanitation needs | If I need to use a sanitation location when away from home during the day, I can go: |
| F1 | Freedom of movement for meeting personal sanitation needs | If I need to use a sanitation location at night, I can go to my household’s daytime sanitation location, I can go: |
| F2 | Freedom of movement for attending sanitation-focused events | If I wanted to go a sanitation-focused public gathering, community meeting, or training outside of my neighborhood, I could go: |
| F2 | Freedom of movement for attending sanitation-focused events | If I wanted to go a sanitation-focused public gathering, community meeting, or training near my home, I could go: |

**Supplementary Table O: Institutional Structures domain scales that have been finalized after Phase 2, by subdomain and factor (2 of 2 scales in Institutional Structures domain).**

| **Finalized Institutional Structures Scales** | | |
| --- | --- | --- |
| **Subdomain:** Norms  **Response Options:** Strongly agree; agree; disagree; strongly disagree | | |
| **Factor** | **Factor Name** | **Item** |
| F1 | Gendered household roles and responsibilities | In this community, it is women more often than men who are expected to assume most responsibilities related to maintaining the cleanliness of the sanitation location the family uses. |
| F1 | Gendered household roles and responsibilities | In this community, it is women more often than men who are expected to accompany or support elderly, sick, or disabled family members who cannot urinate or defecate on their own. |
| F1 | Gendered household roles and responsibilities | In this community, it is women more often than men who are expected to accompany or support children in their household when they need to urinate or defecate. |
| F1 | Gendered household roles and responsibilities | In this community, it is women more often than men who are expected to clean feces from their home or household compound from children or other family members. |
| F1 | Gendered household roles and responsibilities | In this community, it is women more often than men who are expected to who are expected to wash clothes that become dirty as a result of sanitation conditions or accidents. |
| F1 | Gendered household roles and responsibilities | In this community, it is women more often than men who are expected to clean their children after defecation if needed. |
| F2 | Gendered expectations surrounding sanitation value chain work | Even if women were trained, it would be socially unacceptable for women to do construction, repairs, or upgrades for latrines. |
| F2 | Gendered expectations surrounding sanitation value chain work | Technical work, like latrine construction, repairs, or upgrades, should be done by men, not women. |
| F2 | Gendered expectations surrounding sanitation value chain work | Emptying latrine pits should be done by men, not women. |
| F3 | Restrictions on women speaking at sanitation-related meetings | At a sanitation-related meeting where both men and women are present, women should only speak when they are asked to do so. |
| F3 | Restrictions on women speaking at sanitation-related meetings | At a sanitation-related meeting where both men and women are present, women should only speak after all the men have shared their opinions. |
| F3 | Restrictions on women speaking at sanitation-related meetings | At a sanitation-related meeting where both men and women are present, women should not speak. |
| F4 | Acceptability of women’s participation in sanitation-related meetings | It is appropriate for women to attend sanitation-related meetings where men are present. |
| F4 | Acceptability of women’s participation in sanitation-related meetings | In this community, it is considered appropriate for a woman to express her opinion about sanitation issues at a community meeting when men are present. |
| F4 | Acceptability of women’s participation in sanitation-related meetings | It is appropriate for women to discuss sanitation-related issues in front of men. |
| F5 | Acceptability of women’s participation in community-level sanitation-related activities | In this community, it is socially acceptable for women to have leadership roles in sanitation-focused committees or organizations. |
| F5 | Acceptability of women’s participation in community-level sanitation-related activities | In this community, it is acceptable for a woman to bring a complaint about a sanitation-problem to a local leader. |
| F5 | Acceptability of women’s participation in community-level sanitation-related activities | It would be socially acceptable for women to organize an initiative to improve sanitation conditions n the community. |
| F5 | Acceptability of women’s participation in community-level sanitation-related activities | If there was a community initiative to improve sanitation, it would be socially acceptable for women to participate. |
| F6 | Acceptability of women disagreeing with their husbands | In this community, it is acceptable for a woman to express a different opinion than her husband in a household discussion about sanitation issues. |
| F6 | Acceptability of women disagreeing with their husbands | In this community, it is acceptable for a woman to express a different opinion than her husband about sanitation issues in front of people outside the family. |
| **Subdomain:** Relations  **Response Options:** Strongly agree; agree; disagree; strongly disagree | | |
| **Factor** | **Factor Name** | **Item** |
| F1 | Scolding for speaking up about sanitation issues | If I speak up about sanitation-related concerns or problems in my household, I may be scolded or punished. |
| F1 | Scolding for speaking up about sanitation issues | If I speak up publicly about sanitation-related concerns or problems, I may be scolded or punished by members of my household. |
| F1 | Scolding for speaking up about sanitation issues | If I speak up publicly about sanitation-related concerns or problems, I may be scolded or punished by members of this community. |
| F1 | Scolding for speaking up about sanitation issues | If I speak up publicly about sanitation-related concerns or problems, I may be scolded or punished by local leaders or authorities. |
| F2 | Familial support for community-level participation | My family would encourage or help me attend a sanitation-related meeting if I wanted to go. |
| F2 | Familial support for community-level participation | My family would encourage or help me to organize an initiative to improve sanitation. |
| F2 | Familial support for community-level participation | My family would encourage or help me to participate in a community initiative to improve sanitation. |
| F2 | Familial support for community-level participation | My family would encourage or help me to take on a leadership role in a sanitation-focused committee or organization. |
| F3 | Relations with service providers and local leaders | My interactions with local leaders or authorities about sanitation-related issues are generally free of conflict. |
| F3 | Relations with service providers and local leaders | When a sanitation-related problem arises in my community, I feel comfortable reporting the problem to a local leader or authority. |
| F3 | Relations with service providers and local leaders | When a sanitation-related problem arises, I feel comfortable reporting the problem to a service provider. |

**Supplemental Figures A-P: Item information curves by sub-domain of empowerment**

**Supplemental Figure A. Item information curve for Bodily Integrity**


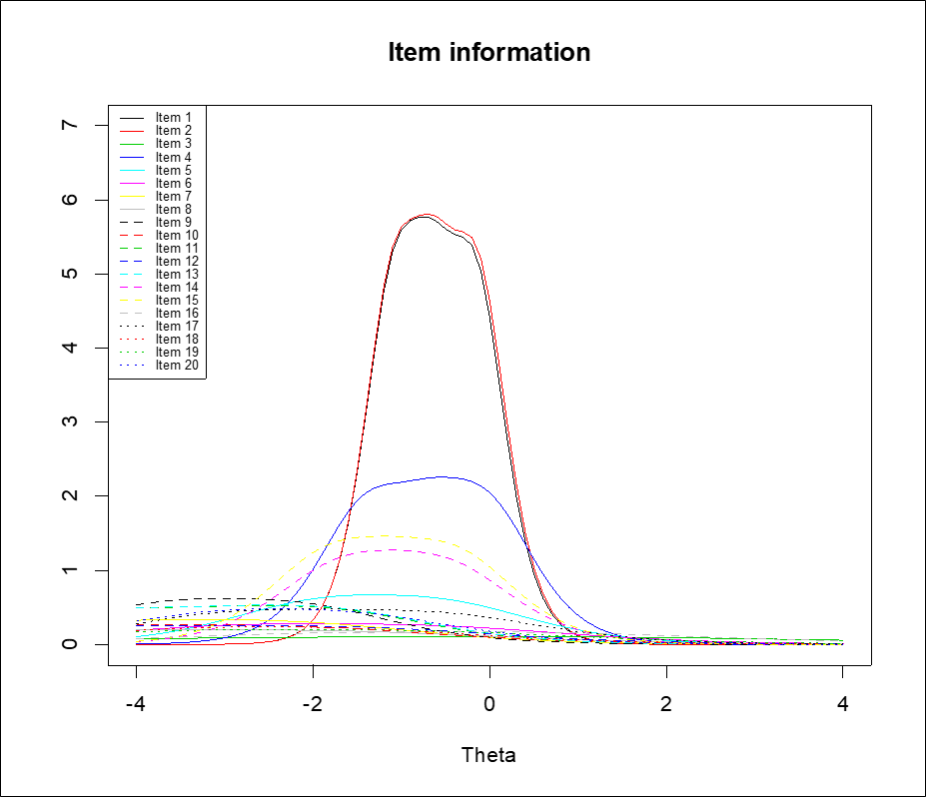


**Supplemental Figure B. Item information curve for Health**

**
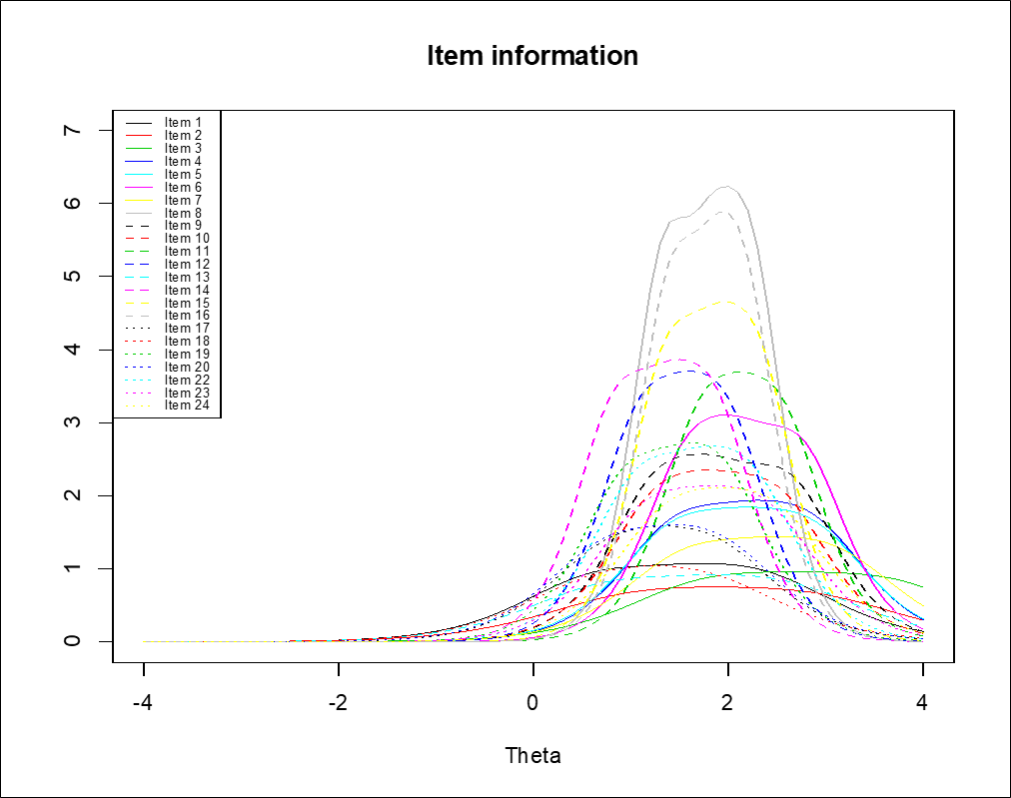
**

**Supplemental Figure C. Item information curve for Safety and Security**

**
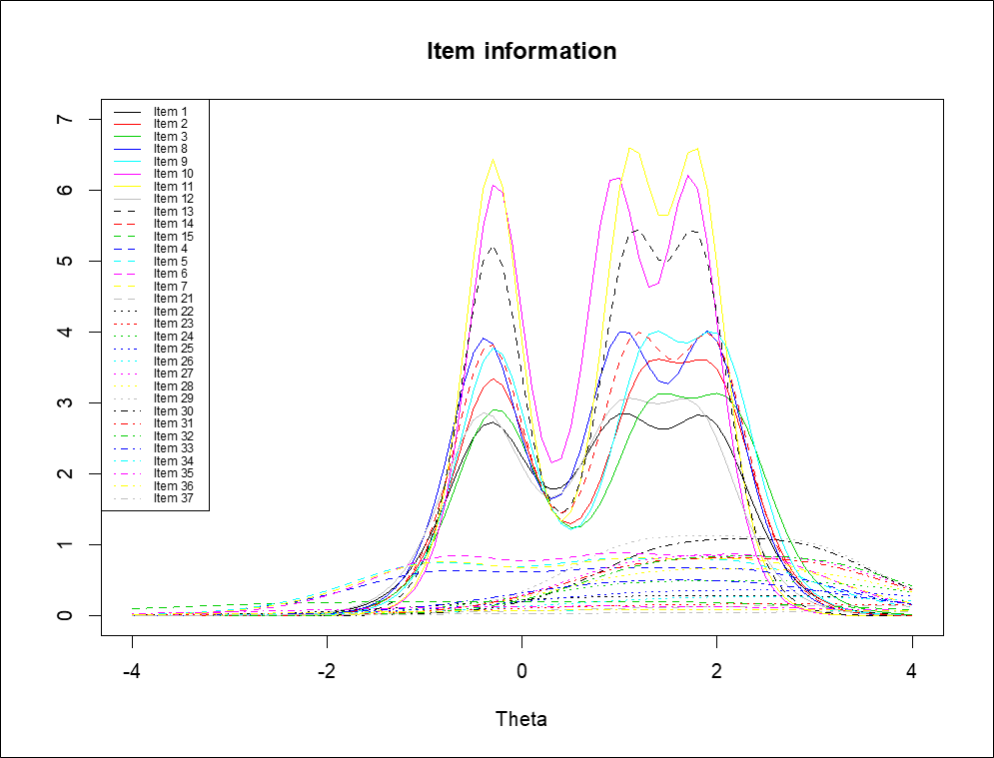
**

**Supplemental Figure D. Item information curve for Privacy**

**
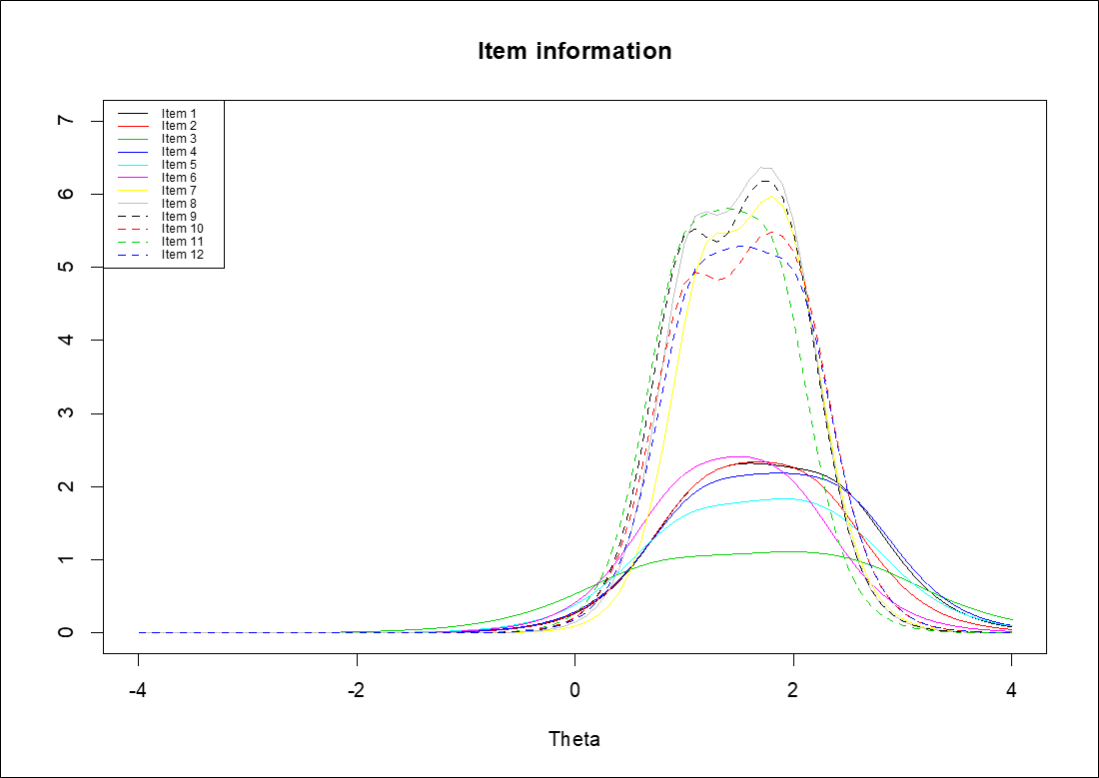
**

**Supplemental Figure E. Item information curve for Financial and Productive Assets**

**
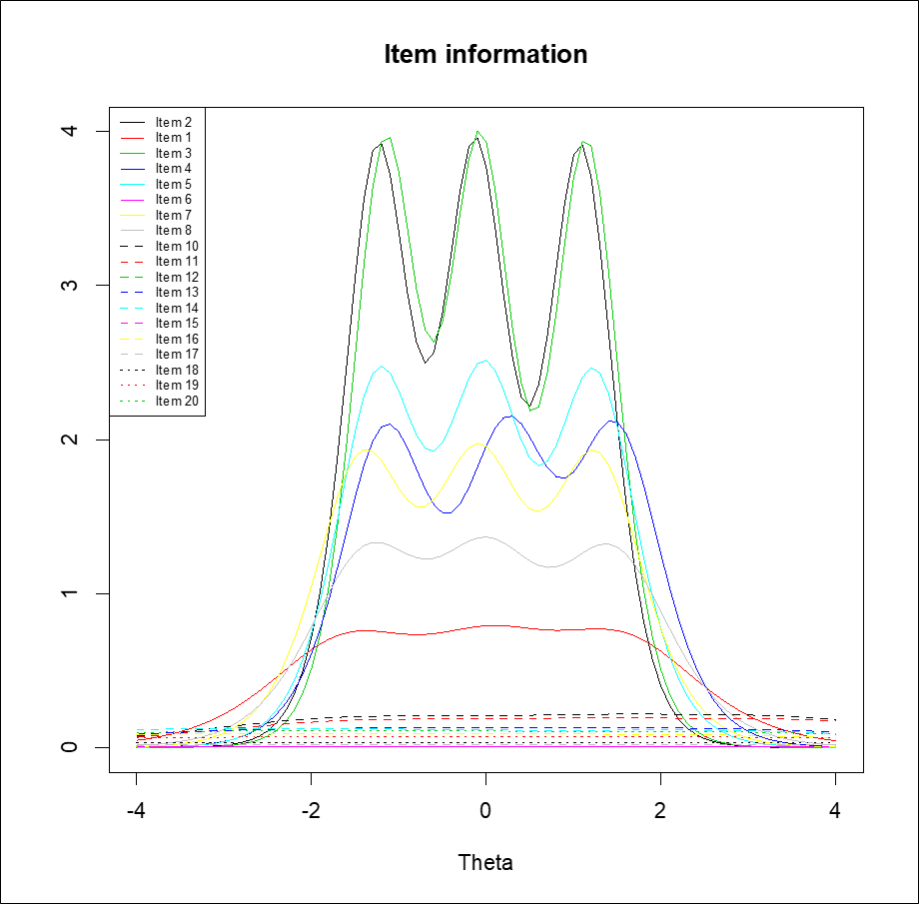
**

**Supplemental Figure F. Item information curve for Social Capital**

**
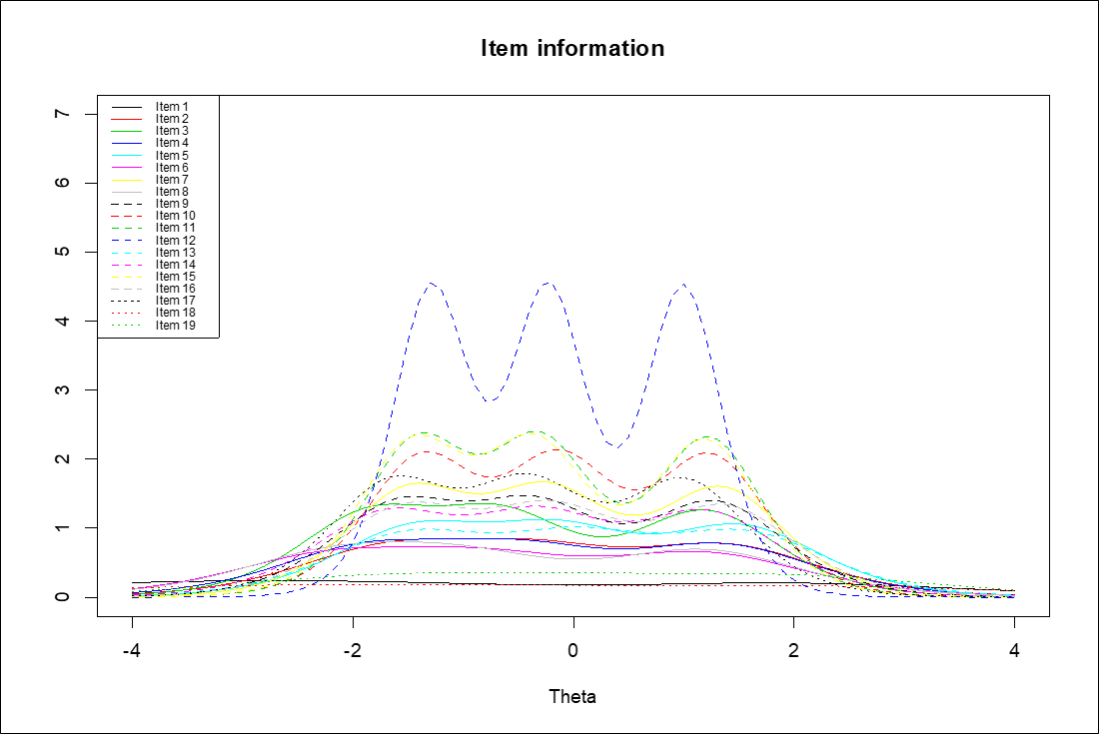
**

**Supplemental Figure G. Item information curve for Time**

**
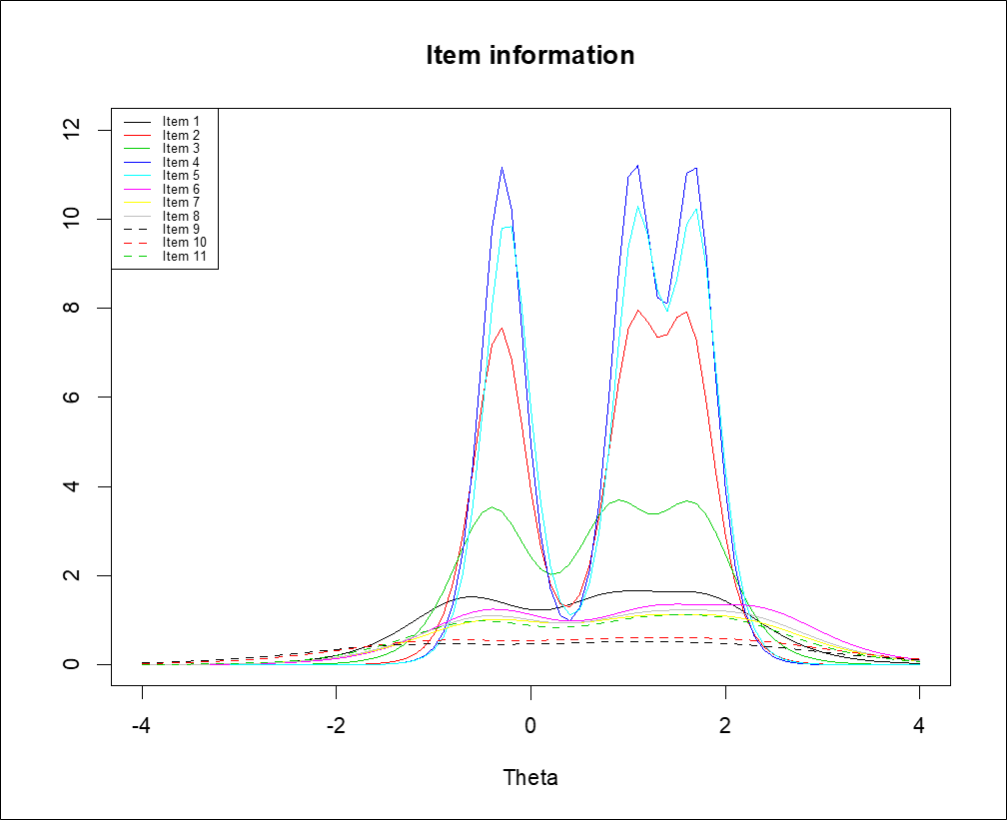
**

**Supplemental Figure H. Item information curve for Knowledge**

**
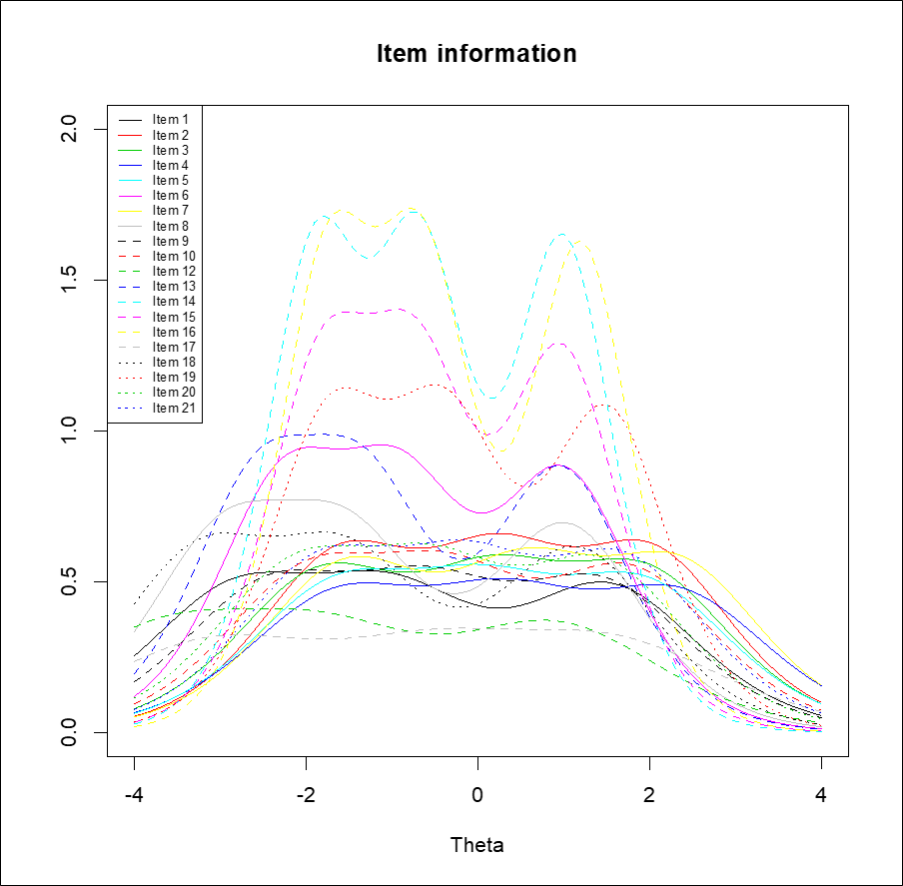
**

**Supplemental Figure I. Item information curve for Critical Consciousness – Scale 1**

**
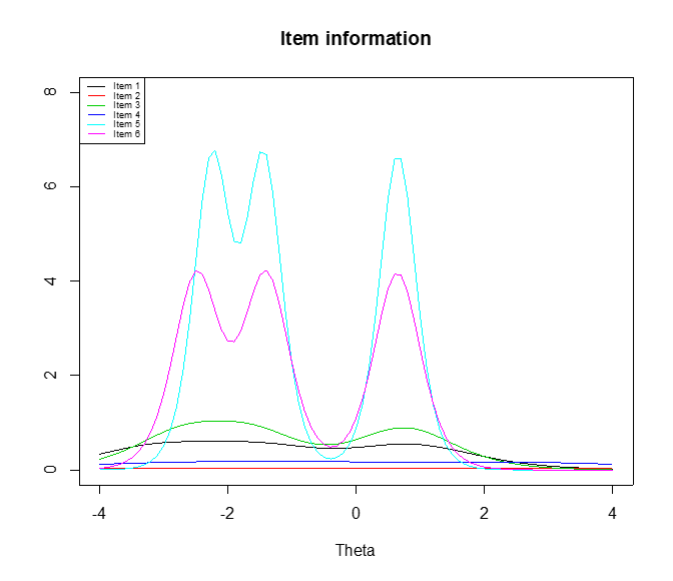
**

**Supplemental Figure J. Item information curve for Critical Consciousness – Scale 2**

**
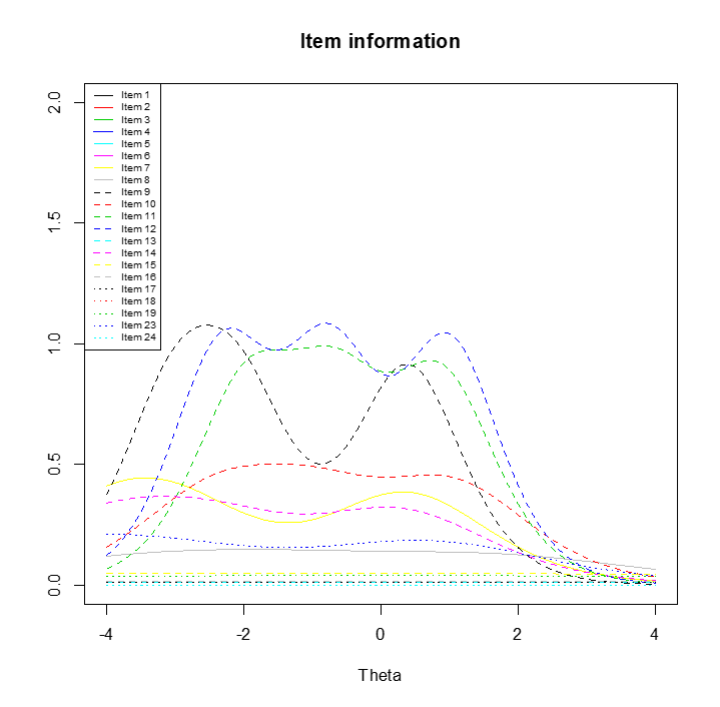
**

**Supplemental Figure K. Item information curve for Leadership**

**
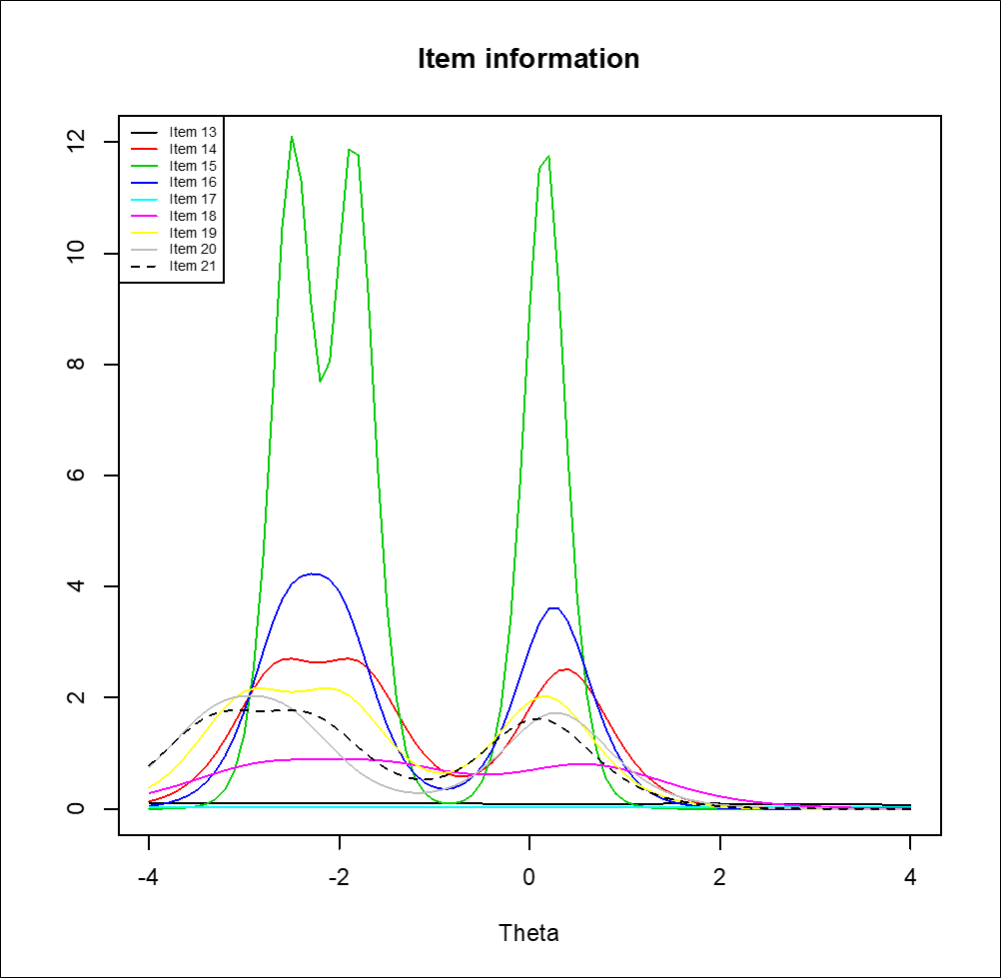
**

**Supplemental Figure L. Item information curve for Decision-Making**

**
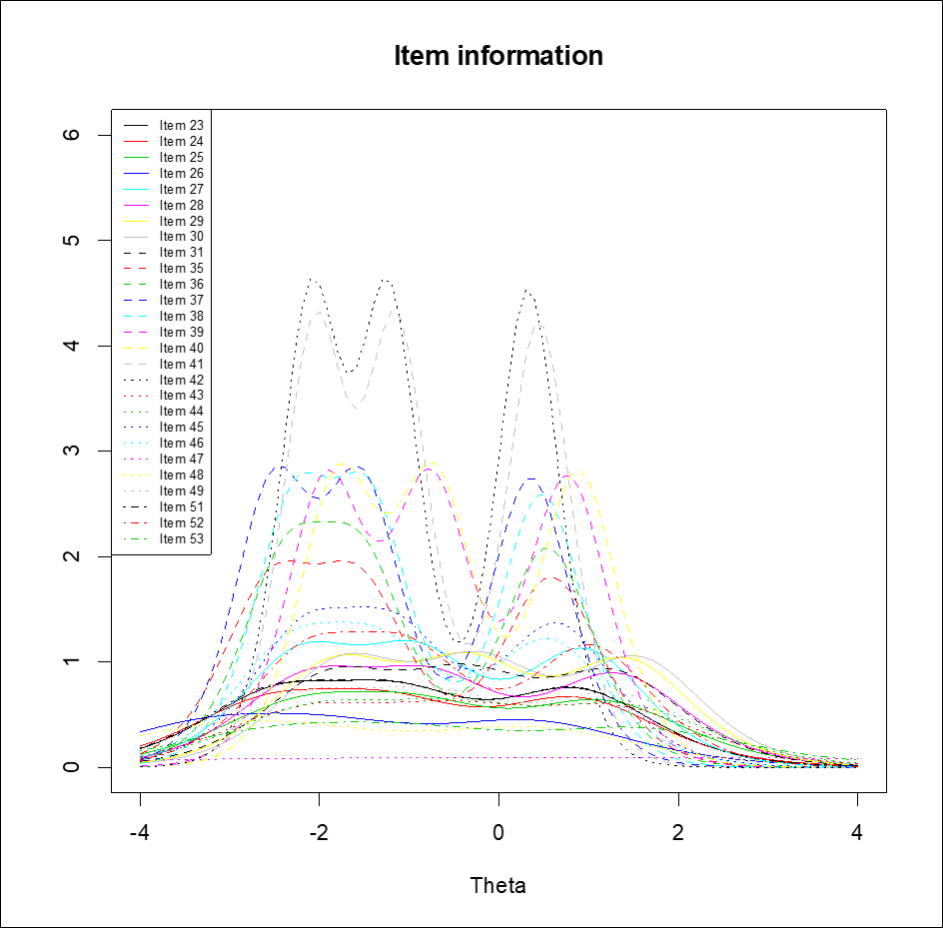
**

**Supplemental Figure M. Item information curve for Collective Action**

**
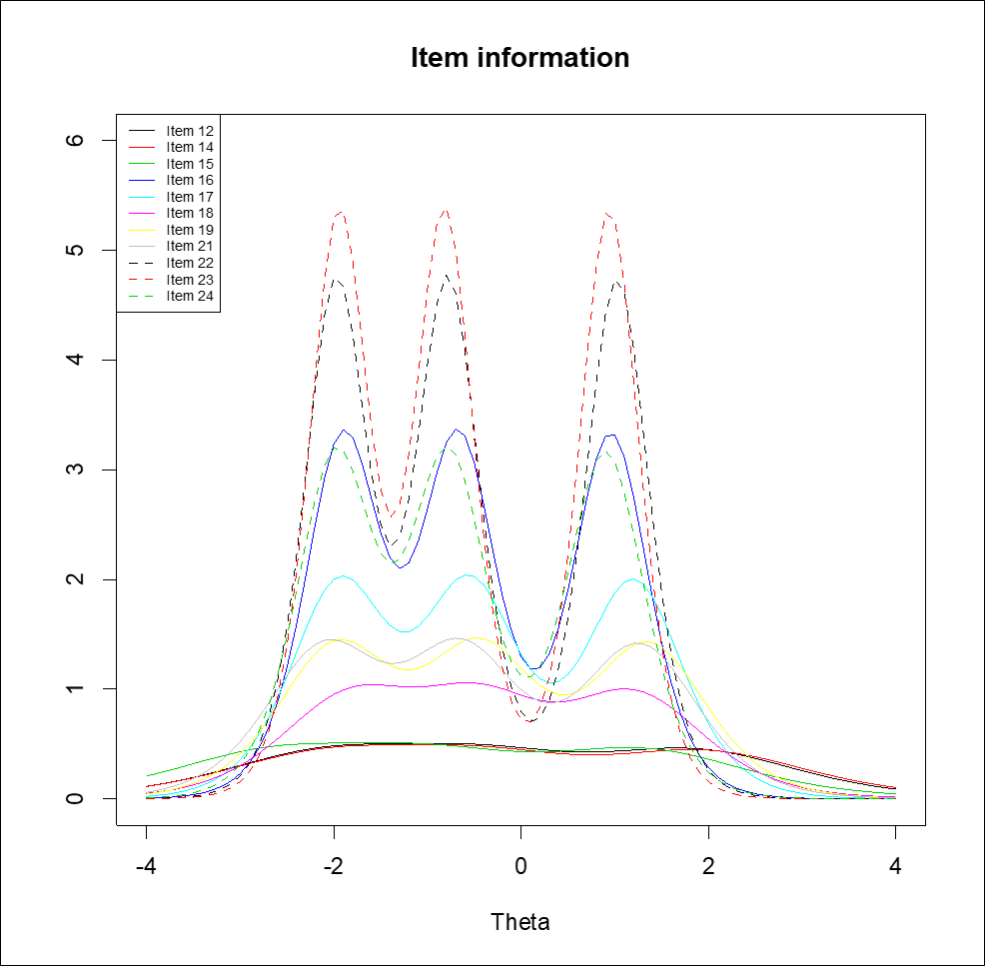
**

**Supplemental Figure N. Item information curve for Freedom of Movement**

**
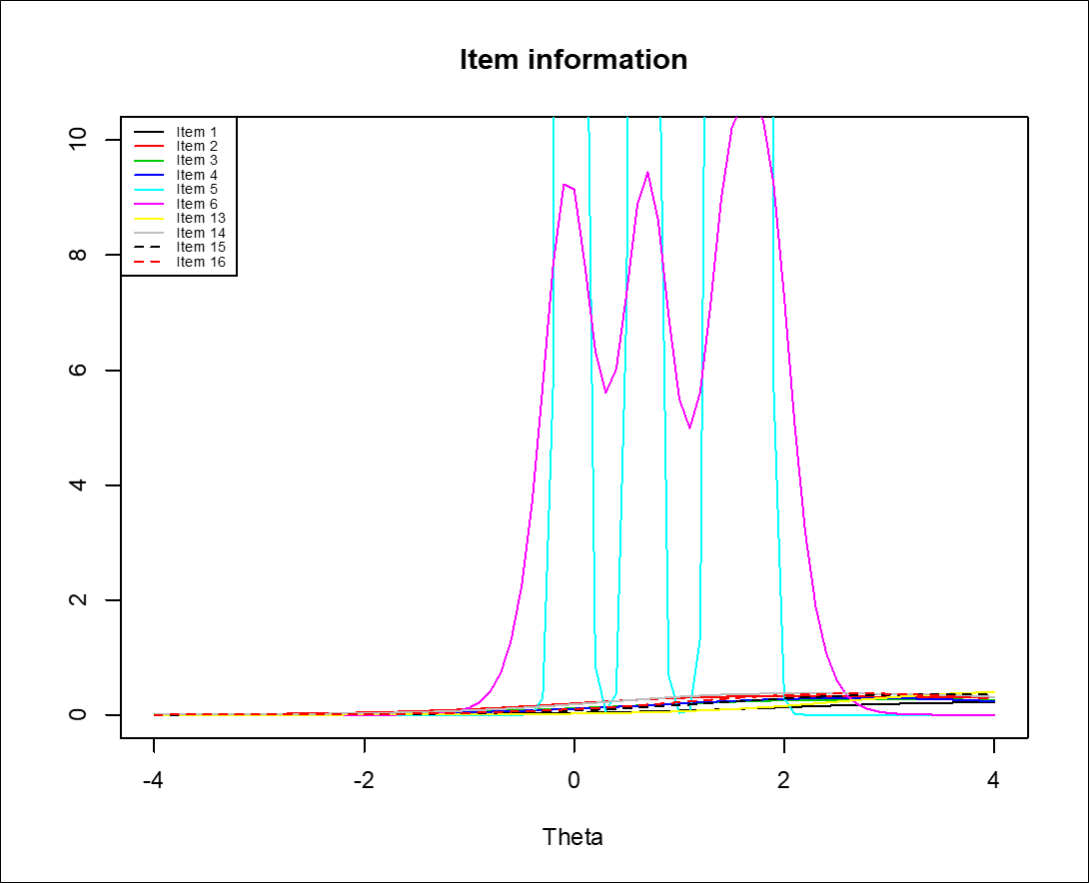
**

**Supplemental Figure O. Item information curve for Norms**

**
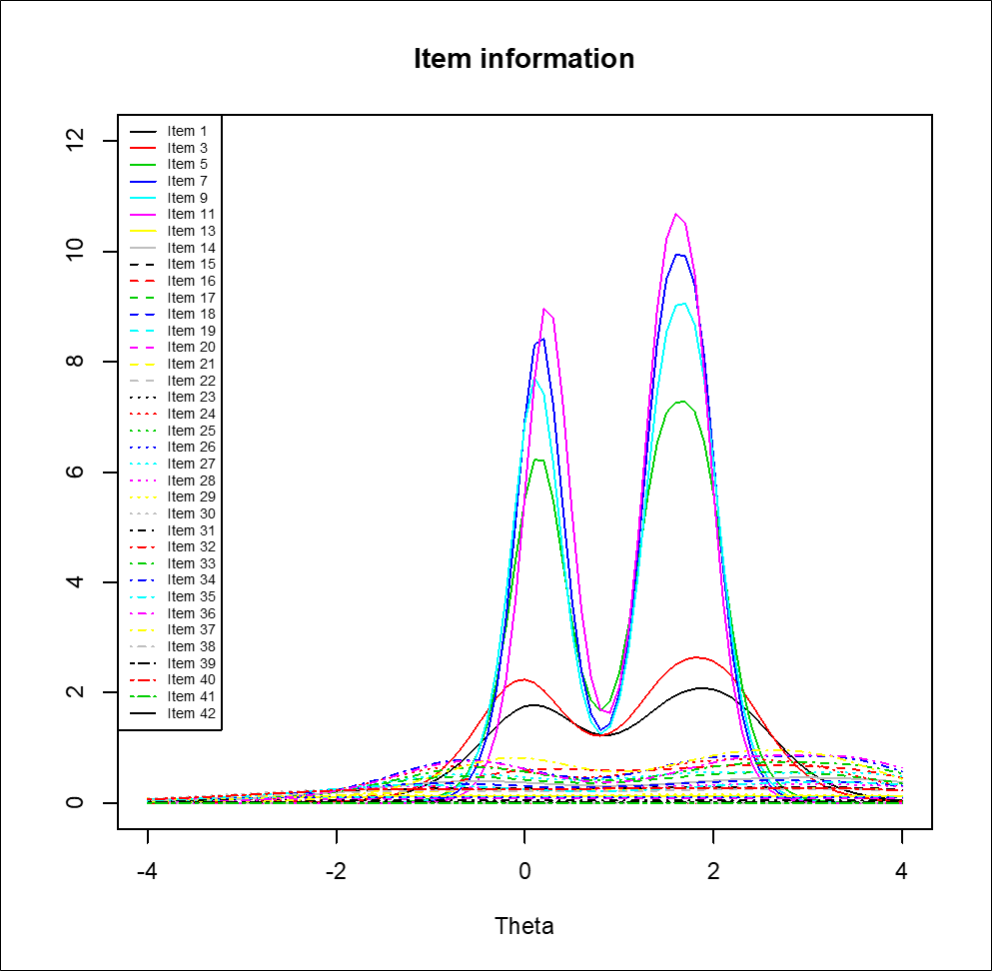
**

**Supplemental Figure P. Item information curve for Relations**

**
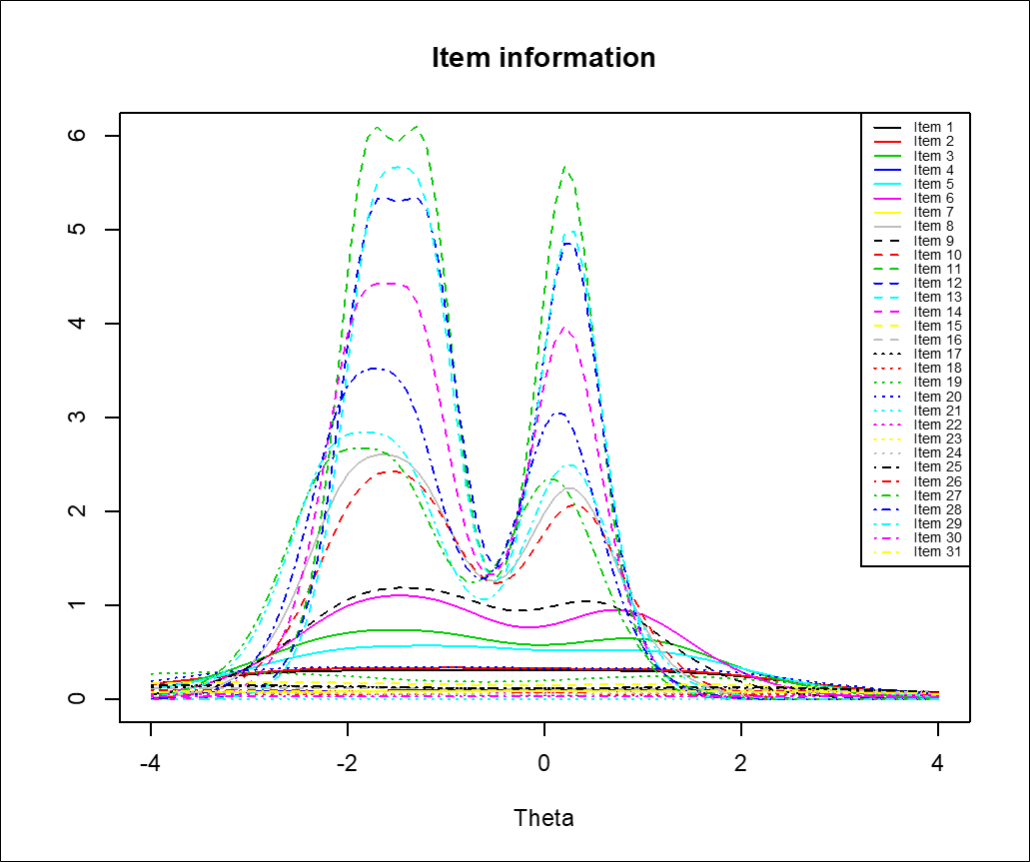
**
